# Time-Dependent c-Myc Transactomes Mapped by Array-Based Nuclear Run-On Reveal Transcriptional Modules in Human B Cells

**DOI:** 10.1371/journal.pone.0009691

**Published:** 2010-03-15

**Authors:** JinShui Fan, Karen Zeller, Yu-Chi Chen, Tonya Watkins, Kathleen C. Barnes, Kevin G. Becker, Chi V. Dang, Chris Cheadle

**Affiliations:** 1 Lowe Family Genomics Core, Division of Allergy and Clinical Immunology, Department of Medicine, The Johns Hopkins University School of Medicine, Baltimore, Maryland, United States of America; 2 Division of Hematology, Department of Medicine, The Johns Hopkins University School of Medicine, Baltimore, Maryland, United States of America; 3 Department of Cell Biology, The Johns Hopkins University School of Medicine, Baltimore, Maryland, United States of America; 4 Department of Oncology, The Johns Hopkins University School of Medicine, Baltimore, Maryland, United States of America; 5 Pathology, School of Medicine, The Johns Hopkins University School of Medicine, Baltimore, Maryland, United States of America; 6 Research Resources Branch, National Institutes on Aging, National Institutes of Health, Baltimore, Maryland, United States of America; Roswell Park Cancer Institute, United States of America

## Abstract

**Background:**

The definition of transcriptional networks through measurements of changes in gene expression profiles and mapping of transcription factor binding sites is limited by the moderate overlap between binding and gene expression changes and the inability to directly measure global nuclear transcription (coined “transactome”).

**Methodology/Principal Findings:**

We developed a method to measure nascent nuclear gene transcription with an Array-based Nuclear Run-On (ANRO) assay using commercial microarray platforms. This strategy provides the missing component, the transactome, to fully map transcriptional networks. ANRO measurements in an inducible c-Myc expressing human P493-6 B cell model reveals time-dependent waves of transcription, with a transactome early after c-Myc induction that does not persist at a late, steady-state phase, when genes that are regulated by c-Myc and E2F predominate. Gene set matrix analysis further uncovers functionally related groups of genes putatively regulated by waves of transcription factor motifs following Myc induction, starting with AP1 and CREB that are followed by EGR1, NFkB and STAT, and ending with E2F, Myc and ARNT/HIF motifs.

**Conclusions/Significance:**

By coupling ANRO with previous global mapping of c-Myc binding sites by chromatin immunoprecipitation (ChIP) in P493-6 cells, we define a set of transcriptionally regulated direct c-Myc target genes and pave the way for the use of ANRO to comprehensively map any transcriptional network.

## Introduction

Deregulated expression of the MYC oncogene, which encodes c-Myc, occurs in about 30% of human cancers, including a substantial fraction of many commonly occurring cancers such as colon, prostate, liver and breast carcinomas [1]. c-Myc (herein termed Myc) is a helix-loop-helix leucine zipper transcription factor that heterodimerizes with a partner protein Max, which is a hub in the network of protein-protein interactions with MAD proteins[2], [3]. Upon heterodimerization, Myc–Max binds specific DNA sites termed E-boxes to activate or repress transcription as well as modulate chromatin structure. Myc competes with Mad proteins via mass action and hetero-dimerizes with MAX. Hence, upon serum stimulation of starved cells, the induction of MYC results in a rapid rise in level of Myc that dimerizes with Max and transregulate target genes. With limiting nutrients or high cellular density, Myc levels in normal cells decrease, resulting in the cessation of cell proliferation. By contrast, cancer cells with deregulated MYC enforce a transcriptional growth response that is independent of external cues.

The composition of the Myc–Max target gene network is likely to be cell-type specific; however, a core set of MYC target genes appears to exist [4]. Genes involved in ribosomal biogenesis have been linked genetically in *Drosophila* to regulation by dMyc and recently shown by genomic analysis to comprise a conserved set of Myc target genes [5]. Myc also directly activates genes involved in nucleolar and ribosomal proteins as well as those regulated by RNA polymerases I and III, including ribosomal RNA genes [2]. Myc may have acquired an expanded role in regulating the cell cycle and energy metabolism later in evolution through affecting the expression of genes involved in glucose metabolism and mitochondrial biogenesis. In higher organisms, Myc has been implicated in the regulation of mRNAs and microRNAs involved in angiogenesis[6]. Many of the conclusions reached to date regarding the Myc target gene networks rely on gene expression changes coupled with Myc binding to target genes as determined by chromatin immunoprecipitation (ChIP)[7], [8].

Numerous ChIP studies have been performed with Myc, and our own study revealed many more binding sites (∼3000 genes) than there are corresponding changes in mRNA levels (∼700) of genes that Myc bound [7], [9], [10]. This observation has been corroborated by Kim et al., who further found in mouse embryonic stem cells that gene expression changes best correlate with multiple transcription factor binding, such that binding of a single transcription factor to any gene loci is infrequently associated with changes at the mRNA level[9]. Hence, the identification of direct target genes for any transcription factor performed to date is limited by assuming that transcription factor binding to target genes results in their altered transcriptional rates. The fact that Myc could also regulate microRNAs suggests that Myc could influence RNA stability as well as translation through its regulation of miRNAs [2], [3] ^,^ [11]. In this regard, it is critical that a tractable approach is developed for the measurement of global changes in nuclear transcription so that regulation of mRNAs at the transcriptional level (the transactome) could be distinguished from post-transcriptional regulation.

Here, we report the development and implementation of an Array-based Nuclear Run-On (ANRO) assay using commercial microarray platforms to further define direct Myc target genes in the P493-6 model of human Burkitt's lymphoma. The human P-493 B cell line has been engineered with an Epstein-Barr Virus (EBV) genome together with a tetracycline-repressible human MYC gene, such that tetracycline-treated resting cells could be recruited into proliferation by the removal of tetracycline, which induces Myc protein expression within three hours[12], [13]. This induction is followed by a coordinated entry of P493-6 cells into the cell cycle with subsequent cell division[14]. Using this system, we performed ANRO at different time points after the induction of Myc and found waves of transcriptional changes (transactome) followed by altered total mRNA levels (transcriptome). Through Gene Set Matrix Analysis (GSMA), we found specific transcription factor motifs enriched in promoters of the different waves of genes following Myc induction. These dynamic transcriptional changes, which heretofore had not been accessible at a global scale, reveal early transcriptional changes that are silenced at steady state when Myc-Max and E2F binding motifs are abundantly found in promoters of transcribed genes. This system is ideal for the identification of early versus delayed early responses to Myc at the transcriptional level as well as post-transcriptional level. Together with genomic binding data, ANRO will provide the heretofore missing transactome for the comprehensive mapping of any transcriptional network.

## Results

### Determination of the Transactome with Array-based Nuclear Run-On Assay

#### Design of a global nuclear run-on assay

**Figure 1:**
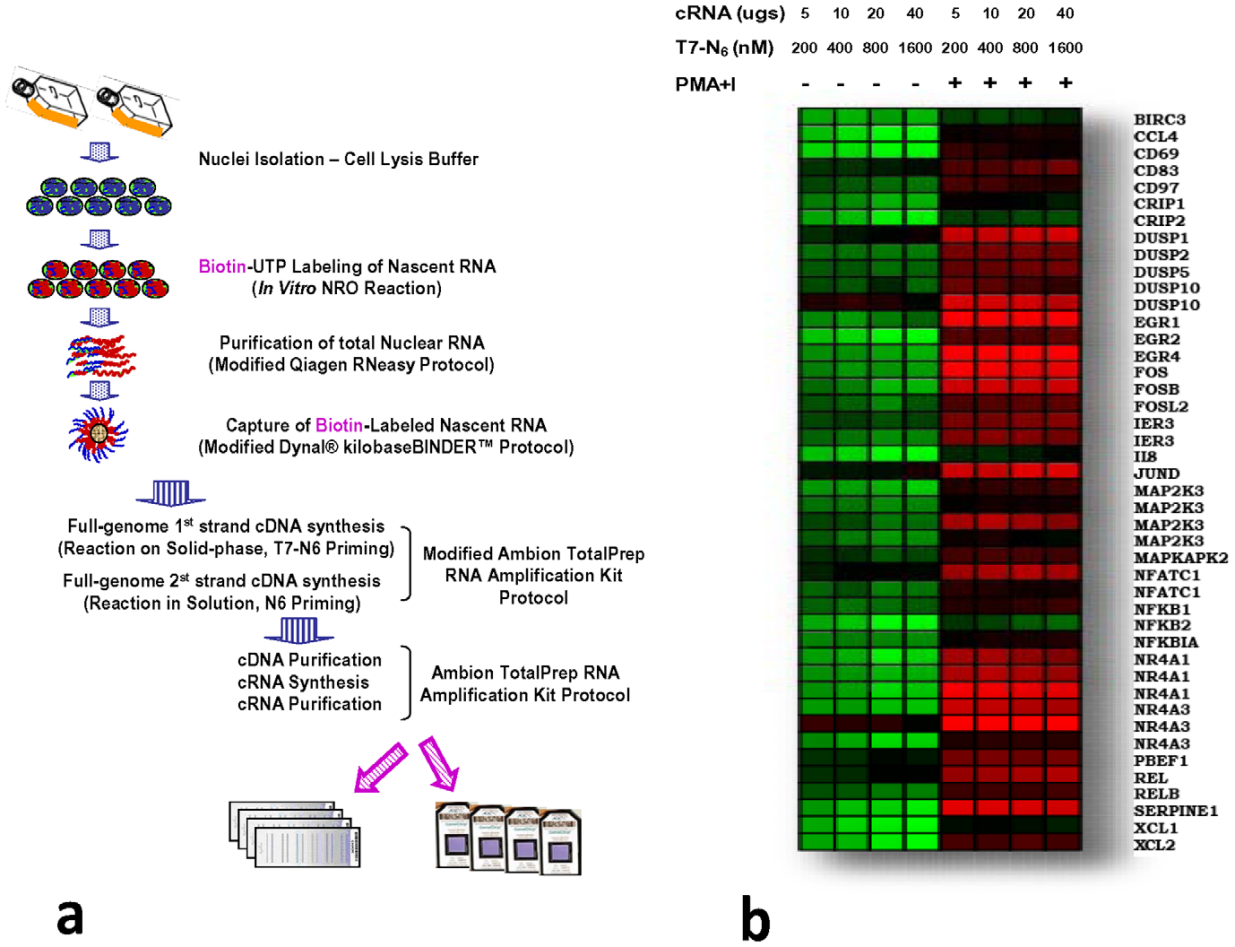
Array- based Nuclear Run-on (ANRO) development and optimization. (**a**) schematic for optimized ANRO protocol. (**b**) heat map of relative gene expression intensities for a panel of test genes activated by PMA+ionomycin induction of human Jurkat T cells. Increasing amounts of T7 random primer used in the first strand cDNA synthesis of Nuclear Run-on RNA accompanied by increasing amounts of the resulting cRNA generated and applied to Illumina microarrays show no increase in signal.

Based upon our previous experience with a spotted filter-array-based nuclear run-on (NRO) using radiolabeled UTP[15], [16], our initial goal (**Figure 1a**) was to substitute a non-radioactive labeled nucleotide (biotin-UTP) during the metabolic labeling of primary nuclear run-on RNA in order to select for newly transcribed (nascent) RNA only. Nascent RNA labeled in this manner could be captured on streptavidin beads and first strand cDNA generated while the RNA remained immobilized on beads [17]. We generated first strand cDNA with bead-immobilized nascent RNAs using random primers coupled with a T7 promoter sequence, released first strand cDNA from beads by RNase H treatment, followed by second strand cDNA synthesis in solution using random primers only. The NRO cRNA was subsequently generated from column-purified NRO cDNA using T7 polymerase and labeled with biotin-UTP. Purified, labeled NRO cRNA can be then hybridized to most commercial microarray platforms (Illumina, Affymetrix, and Agilent were successfully tested).

We used two biological systems for the design of the ANRO protocol. In one system we used phorbol ester activated Jurkat T cells to determine the genomic responses as we described previously[15], [16]. The other system is the inducible MYC human B lymphoma model cell line P493-6 described above.

#### Assay optimization

We found that ribosomal RNA depletion (ribominus) of NRO RNA resulted in blunted signals for genes whose expression levels were clearly altered. The overall loss of gene information without any major commensurate gene enrichment did not appear to warrant the inclusion of ribosomal RNA removal in our protocols.

We determined the best concentration of T7 random hexamer (T7N_6_) in order to ensure complete NRO first strand cDNA synthesis. **Figure 1b** shows an example of optimization results amongst replicate samples demonstrating that further increases in primer and hybridization concentration combinations did not increase overall signal intensities for a panel of previously identified genes regulated by PMA and ionomycin treatment of Jurkat T cells [18]. This observation indicates that we have optimized the conditions and that the primer concentration for NRO is not a limiting factor with the Illumina microarray. We also found that the chronological order of binding of biotin-labeled NRO RNA to streptavidin beads was vital for high efficiency. For example, first strand cDNA synthesis in solution followed by binding to beads was, surprisingly, much less effective than pre-binding of NRO RNA to beads followed by solid-phase-based synthesis. Similarly binding times, temperature and buffer conditions were explored thoroughly and the detail protocol is provided in the Supplementary section. It was empirically determined that the RNAse H step for degrading template RNA following first strand cDNA synthesis was indeed effective for releasing the 1^st^ strand cDNA product from the streptavidin beads, since the fully synthesized double stranded cDNA product remained quantitatively in solution following bead centrifugation and removal.

#### Assay reproducibility and transcript length

**Figure 2:**
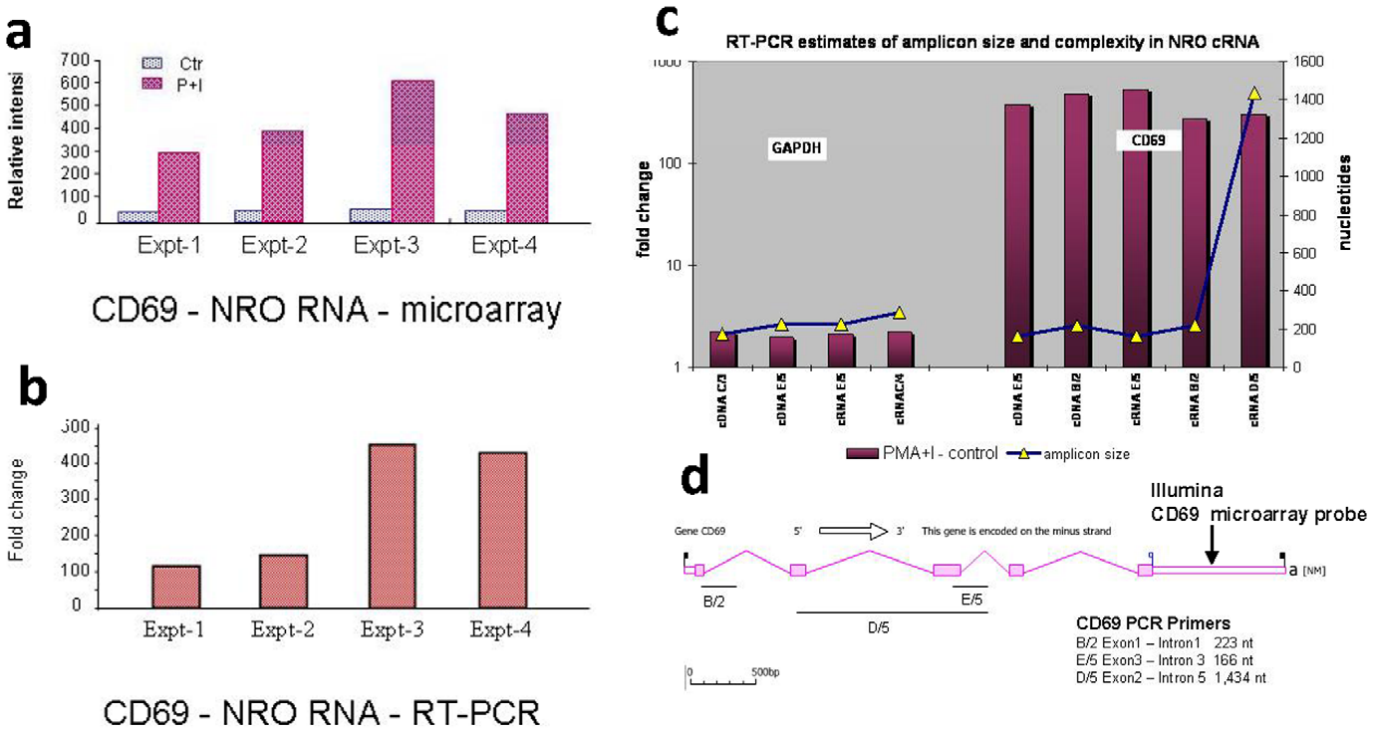
Array- based Nuclear Run-on (ANRO) characterization. (**a, b**) Transcriptional regulation of CD69 in NRO RNA following Jurkat T Cell activation. (**a**) Illumina microarray - CD69 average signal intensity for 4 independent experiments. (**b**) CD 69 Real time PCR validation for the same experiments (fold changes were normalized by GAPDH controls). (**c, d**) RT-PCR estimates of varying amplicon size and complexity for CD69 gene transcription in PMA + I activated Jurkat T cell NRO RNA. (**c**) Fold changes for CD69 in NRO RNA were tested using either first strand cDNA or the final amplified cRNA product (as indicated). Multiple amplicons of GAPDH were similarly tested as an uninduced control. (**d**) CD69 amplicon primer design spans exon-intron genomic sequences of sizes as indicated (primer sequences are reported in Materials and Methods). Note the location of the Illumina microarray probe located at the 3′UTR.

With the optimized conditions, we sought to determine the reproducibility of the techniques involved and to characterize the transcripts produced by these methods. **Figure 2a** shows the increase in transcription of CD69, one of the key genes induced by T cell activation, and that NRO Illumina arrays faithfully reproduced the proportional enrichment of CD69 primary mRNA as measured by quantitative RT-PCR in each sample (**Figure 2b**). In addition, we have validated a number of other genes that were detected by ANRO (Supplemental **Table S1**)

A major concern with the use of conventional microarrays combined with NRO is that the probe sequences on commercial platforms are biased toward the 3′ end of genes, while NRO transcripts could be biased to the 5′ end and therefore remain undetected if full length transcripts were not generated with the NRO procedure. Hence, we tested CD69, which is one of the most highly regulated genes in PMA plus ionomycin activated Jurkat T cells in both the NRO and total RNA [18].

A series of RT-PCR primers were designed to span exon-intron junctions across CD69 (**Figures 2c, d**). The results showed that in each case, a large and dramatic up-regulation of CD69 was detected even with very large amplicon sizes (1400 nt) in both synthesized cDNA and amplified cRNA. Additional characterization of NRO amplified transcripts using Affymetrix Exon arrays demonstrated that ANRO was indeed capable of detecting very long transcripts reproducibly (Supplemental **Figure S1**). This observation is compatible with the ability of the single Illumina microarray probe at the CD69 3′UTR (Figure 2d) to detect NRO cRNAs. Furthermore, we found by substituting T7-Oligo dT primers for T7N_6_ in the first strand synthesis in the ANRO process that about 20% of the biotinylated NRO material was already polyadenylated (data not shown), indicating rapid processing of primary transcripts to mature mRNAs.

#### Cross platform comparisons

**Figure 3:**
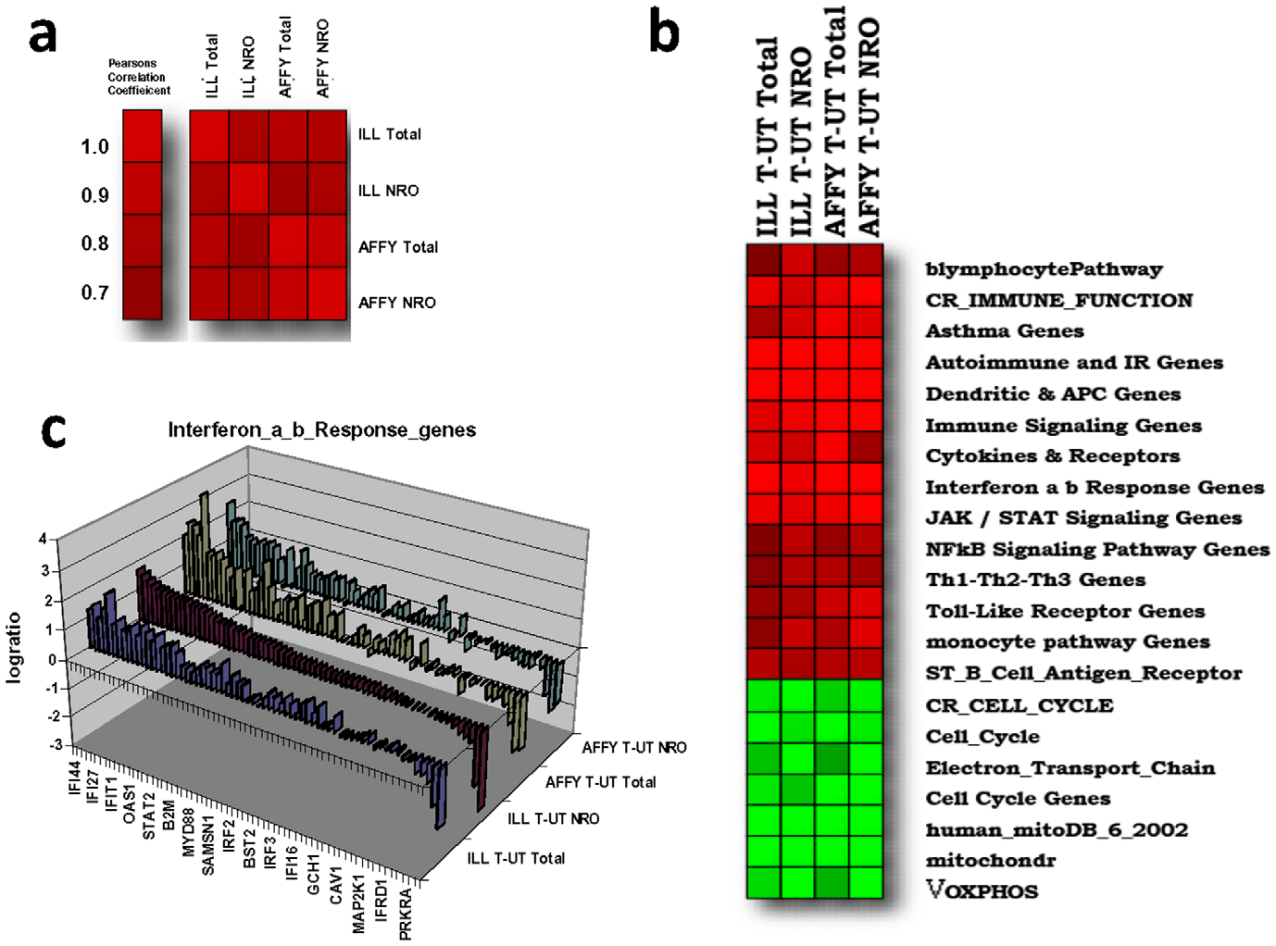
Array- based Nuclear Run-on (ANRO) shows consistent results between Affymetrix exon arrays and Illumina BeadChips. (**a**) Pearson's Correlation Matrix of changes in gene expression (log ratios) between tet treated (T) and untreated (UT) P493-6 human B cells following 48 hr induction for a common pool of approximately 7800 genes as measured on both Illumina BeadArrays and Affymetrix Exon microarray platforms. (**b**) heat map illustrating a selection of the highest scoring pathways using Gene Set Matrix Analysis (GSMA) on the logratios from (**a**). (**c**) Pathway breakout from **b** on a gene by gene basis for the Inteferon a, b Response Genes. Log ratios sorted descending on the NRO as measured on the Illumina platform ((ILL T-UT NRO).

To further establish the applicability of ANRO on different commercial microarray platforms, we calculated statistically significant regulated gene expression determined from a parallel series of experiments using both the Affymetrix exon array and Illumina BeadArrays. Changes in gene expression of a common pool of genes (∼7800) were determined for tetracycline-treated (tet) and untreated (MYC over-expression) human P493-6 B cells for NRO and total RNA using both microarray platforms. The results (**Figure 3a**) demonstrate excellent comparability between platforms (PCC = 0.82), treatments (PCC = 0.88), and methods (PCC = 0.83).

Pathway analysis of the combined Affymetrix and Illumina datasets demonstrates a high degree of consistency between microarray platforms and between NRO and total RNA at the geneset level (**Figure 3b**). This consistency is maintained on a gene-by-gene basis as illustrated for interferon response genes up-regulated by Myc over-expression (**Figure 3c**).

### Definition of the Myc target gene network using ANRO

The availability of ANRO permits the study of transcriptional responses to Myc activation as a function of time. First, we sought to determine transcriptional differences between P493-6 cells proliferating with high MYC expression and P493-6 cells that have been treated with tetracycline for 48 hr when these cells do not proliferate and have very low MYC expression. **Table 1** shows the most highly up-regulated genes at 48 hrs of MYC over-expression in the P493-6 B cell system, including MYC gene itself. Genes highly up-regulated as measured in the total RNA preparations were similarly up-regulated in the NRO RNA and overlapped with many known MYC target genes as shown^7^. Myc target genes, such as *NME1* and Myc induced nuclear antigen (*MINA*) are prominently represented in this group. The study also revealed that many genes involved in nucleotide metabolism such as *ACLY*, *AK3*, *AK5*, *APRT*, *ATP5B*, *ATP5D*, *ATP5I*, *ATP5O*, *GART*, *IMPDH2*, *NME1*, *NME2*, *PAICS*, *PFAS*, and *PPAT* are among mRNAs significantly increased both at the steady state level and at the nuclear run-on level with MYC induction, indicating that they are all transcriptionally up-regulated. Ontology analysis of genes that were transcriptionally up-regulated using the EASE algorithm [19] indicates that Myc-responsive nucleotide metabolic genes are highly statistically over-represented at p<10^−7^. These observations are compatible with our identification of nucleotide metabolic genes as direct Myc target genes [20] .

**Table 1:** Selected results for statistically significant changes in gene expression from either Array-based NRO or total RNA as measured on the Illumina microarray platform.

Symbol	Gene Name	Total RNA (fold change)	NRO (fold change)	known Myc target
MYC	v-myc myelocytomatosis viral oncogene homolog (avian)	13.69	13.09	Y
NME1	non-metastatic cells 1	7.05	10.83	Y
DSCR2	Down syndrome critical region gene 2	7.31	6.69	Y
PAICS	phosphoribosylaminoimidazole carboxylase	6.42	6.54	Y
EXOSC5	exosome component 5	5.23	6.29	Y
DNAJC12	DnaJ (Hsp40) homolog, subfamily C, member 12	6.45	6.15	Y
GEMIN5	gem (nuclear organelle) associated protein 5	4.11	5.66	
SLC7A5	solute carrier family 7 (cationic amino acid transporter, y+ system)	10.84	5.45	
PPAT	phosphoribosyl pyrophosphate amidotransferase	7.32	5.37	Y
IMPDH2	IMP (inosine monophosphate) dehydrogenase 2	5.32	4.99	Y
MCM4	MCM4 minichromosome maintenance deficient 4	4.56	4.70	Y
ACAT1	acetyl-Coenzyme A acetyltransferase 1	4.06	3.85	Y
GEMIN4	gem (nuclear organelle) associated protein 4	3.26	3.54	Y
NME2	non-metastatic cells 2	2.78	3.49	
APRT	adenine phosphoribosyltransferase	1.90	3.24	Y
CDK4	cyclin-dependent kinase 4	3.22	3.16	Y
ATP5D	ATP synthase, H+ transporting, mitochondrial F1 complex, delta subunit	1.77	2.55	Y
DDX1	DEAD (Asp-Glu-Ala-Asp) box polypeptide 1	2.11	2.53	Y
MINA	MYC induced nuclear antigen	2.83	2.52	Y
NME4	non-metastatic cells 4	2.55	2.46	Y
ACLY	ATP citrate lyase	1.96	1.99	
CEBPB	CCAAT/enhancer binding protein	1.62	1.98	Y
BUB3	BUB3 budding uninhibited by benzimidazoles 3 homolog	1.58	1.57	Y

Fold changes are calculated between cells treated with tetracycline for 48 hrs versus untreated cells. Known MYC targets are as indicated (Y = yes).

**Figure 4:**
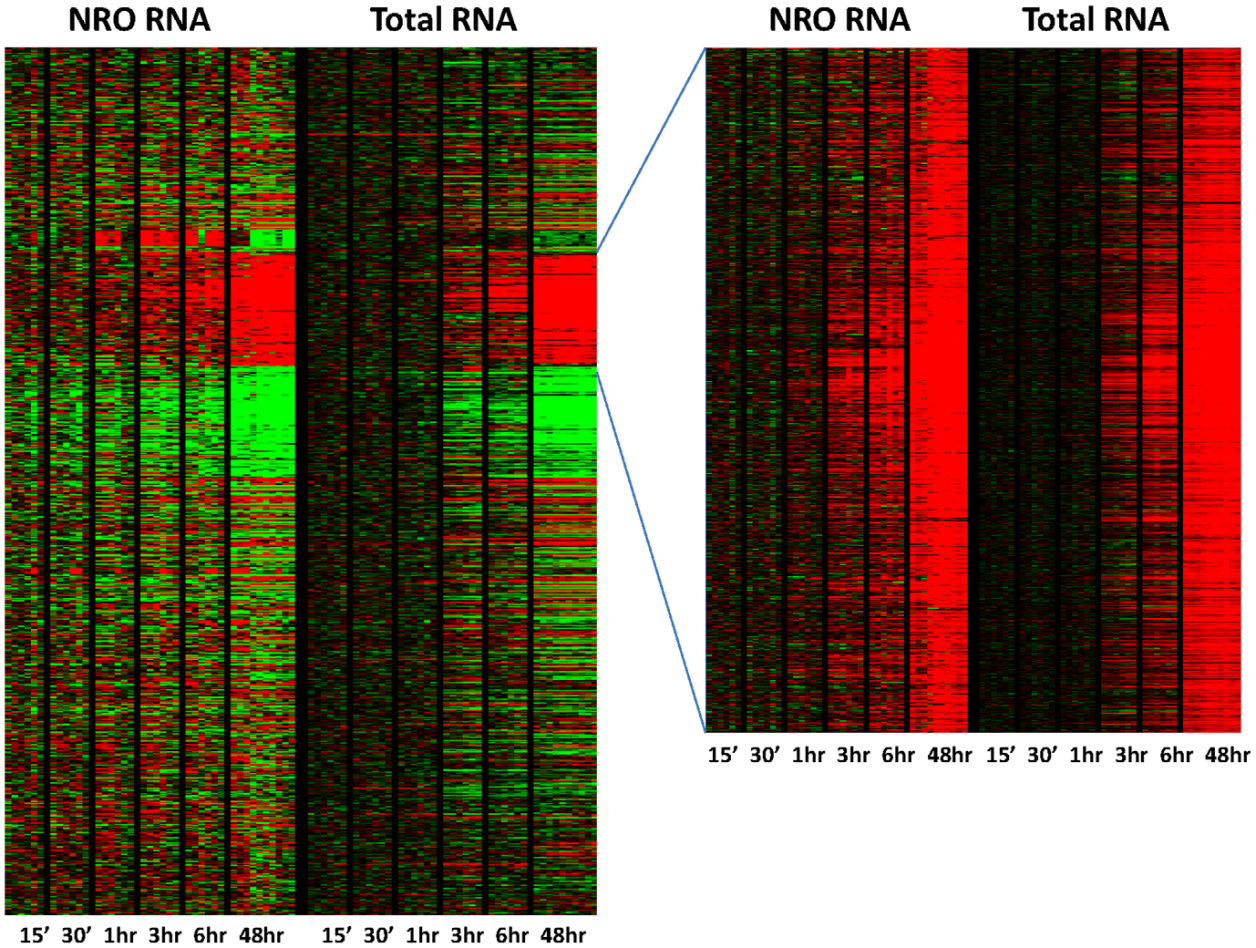
Heatmap of a time course of gene regulation in human P493-6 B cells following Myc gene induction. At each time point, 6 independent experiments were performed, except for the 48 hr time point that has 10 experiments shown. Coordinate increase in gene expression for 970 genes is contrasted between nascent nuclear gene transcription (NRO RNA) and changes in gene expression at the whole cell level (Total RNA) at the timepoints indicated. Changes in gene expression are calculated as logratios relative to 0time. Inset shows all changes measured (10,530).

After having established NRO sample reproducibility and demonstrated comparability between transcription measured at either the nuclear or whole cell level in the MYC model system after 48 hr, we next tested the ANRO method across a time course of MYC overexpression and compared the results with conventional microarray using total RNA. P493-6 cells were grown in the continuous presence of tetracycline (MYC off), briefly washed in PBS, and then incubated without tetracycline. Sequential parallel samples were processed at 15 min, 30 min, 1 hr, 3 hr, and 6 hr for total RNA or NRO RNA isolation and labeling. **Figure 4** illustrates this time course for all calculated changes in gene expression as measured in NRO and total RNA. This study illustrates a very tight concordance between both methods with a significant difference that NRO appears to anticipate changes in gene expression at time points earlier than they appear in total RNA, consistent with altered transcriptional rates followed by changes in total RNA.

**Figure 5:**
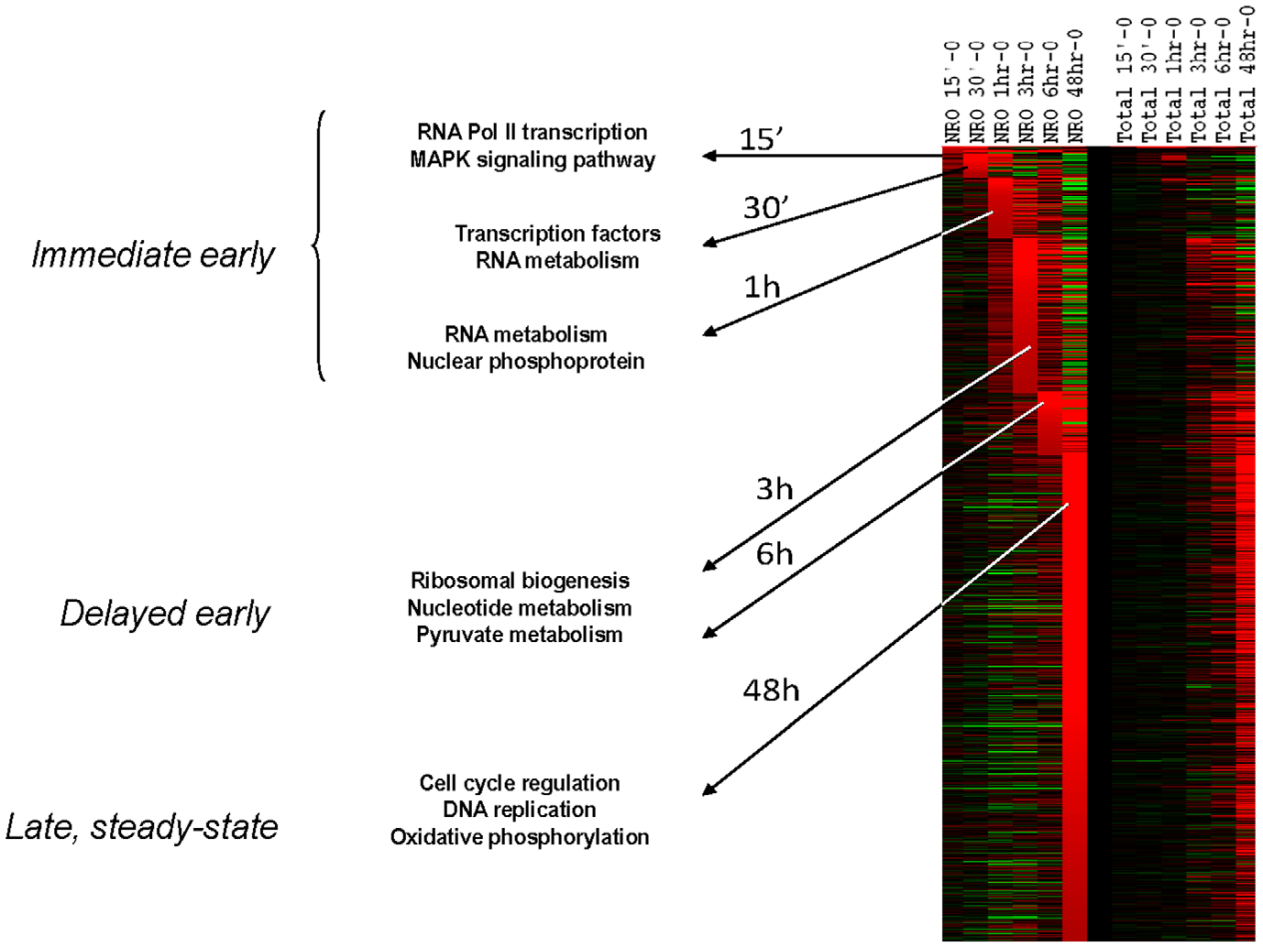
Pathway enrichment analysis of changes in gene expression sorted by their first appearance as upregulated in the NRO RNA. Identical analyses were run for both NRO and total RNA as indicated. Examples of time-dependent up-regulated pathways at early, middle, and late time points.

As seen in **Figure 5** (see Supplemental **Table S2 and S3** for accompanying gene lists), Myc activation is followed by waves of gene expression changes that accompanied transcriptional activation of clusters of genes. These experiments provide the first direct observation of global transcriptional changes as a function of time following Myc activation. We identified clusters of genes that respond rapidly to Myc (within 1 hr), those that have a delayed early response (after 3–6 hr), and those occurring late or at steady-state. These time-dependent waves of transcription following Myc activation are likely to represent direct and indirect Myc effects, whose definition is beyond the scope of the current study. Gene ontology analysis reveals that early Myc-mediated transcription involves early response genes regulating MAPK signaling, RNA metabolism and transcription factors, suggesting a program that prepares the cells for S phase entry. The delayed early genes are enriched with those involved in ribosomal biogenesis, nucleotide metabolism and energy (pyruvate) metabolism, indicating the subsequent preparation of cells to increase biosynthetic capabilities for DNA replication. Finally, the late or steady-state transcription involves genes that regulate energy (oxidative phosphorylation), cell cycle or DNA replication.

**Figure 6:**
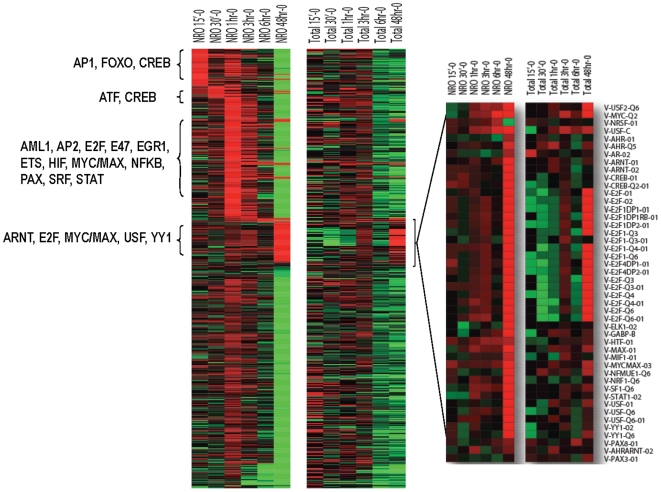
GSMA geneset analysis of coordinate changes in gene expression based on the presence of transcription factor binding sites in selected gene lists. Enrichment values are sorted by their first appearance as upregulated in the NRO RNA. Identical analyses were run for both NRO and total RNA as indicated. Inset highlights genesets up-regulated at 48 hr.

Examination of transcription factor binding motifs by GSMA reveals a stepwise putative involvement of distinct transcription factors following the initial activation of Myc expression in the P493-6 human B cells (**Figure 6**; see Supplemental **Table S4** for accompanying transcription factor motifs lists). Intriguingly, early transcription involves factors that also play a role in immediate early response to serum stimulation such as AP1 and ATF. In the subsequent phases, a wide variety of motifs emerged with Myc/Max, STAT, E2F appearing in the promoters of many genes transcribed at 3–6 hours. Thereafter, at the late steady-state phase, most genes transcribed at the earlier hours are repressed and accompanied by the appearances of genes that are induced putatively by transcription factors Myc/Max, E2F, HIF/ARNT and YY1. These late transcription factors have been implicated in the regulation of nucleotide metabolism and DNA replication. In this regard, earlier waves of transcriptional events would be missed at steady state without ANRO. Many of the earlier phase genes that are later down-regulated are involved in B cell development, indicating that sustained Myc activation leads to the silencing of these differentiation-related genes in favor of those that drive cells into cycle with a sustained proliferative program.

### Global Map of Direct Transcriptional Targets of Myc in Human P493-6 B cells

We superimposed our global Myc binding data from our earlier ChIP-PET studies^7^ with the ANRO results and derived a minimal set of direct transcriptional targets of Myc in the P493-6 system (**Table 1** and Supplemental **Table S5**). Gene set enrichment analysis (GSEA; http://www.broad.mit.edu/gsea/index.jsp) of over 200 direct Myc up-regulated target genes in P493-6 cells reveals a significant overlap with embryonic (p = 3.2e^−16^) or neural (p = 9.4e^−19^) stem cell gene sets. This observation is consistent with the suppression of B cell differentiation genes in the time course ANRO study discussed above. Moreover with GSEA, the set of 340 direct down-regulated target genes most striking overlap with the receptor CD40-upregulated gene set (p = 2.4e^−18^, BASSO GERMINAL CENTER_CD40_UP), which are involved in germinal center development and generation of memory B cells. It is notable that the ChIP-PET studies were incomplete since only 60 percent sequencing saturation of the ChIP-PET library was achieved^7^. Hence, it is critical to note that this is only a minimal set, pending further ChIP-chip studies to re-map Myc binding sites in the P493-6 system. Nonetheless, the ANRO approach we describe here is generally applicable to all transcription factors using commercially available arrays coupled with our protocol.

## Discussion

We report here the global measurement of transcription by adapting the nuclear run-on assay to commercial microarrays. Our approach contrasts with a recently published nuclear run-on assay using direct sequencing of cDNA libraries from immunoaffinity captured Br-UTP labeled nascent RNAs without the accompanying analysis of total RNA levels [21]. ANRO, which relies on the 10^−15^ M Kd of streptavidin-biotin binding, also contrasts with the use of thiouridine-labeled nascent RNAs which were captured on lower binding affinity to organomercurial matrix [22]. In addition, the use of commercially available microarrays as describe in our study makes our method technically and economically more accessible to most researchers. In fact, we found that both Affymetrix and Illumina microarrays were equivalent for ANRO, making ANRO more widely accessible. Specifically, we developed, optimized and applied this approach, termed Array-based Nuclear Run-On (ANRO), to map the Myc transcriptional network in a human B cell lymphoma model bearing an inducible Myc construct. It is notable, however, that ANRO measures transcription with isolated nuclei rather than intact cells. Hence, a more physiological reflection of transcription will require future technical advances that enables incorporation of biotinylated UTP in intact cells. Notwithstanding this caveat, the inducibility of Myc enables a time-course study that provides an unprecedented view of global transcriptional responses in isolated nuclei to Myc induction. In particular, we observe waves of transcriptional events following Myc induction that seems to prepare cells for entry into subsequent phases of the cell cycle. Importantly, the initial synchrony of the cells after Myc activation permits the detection of early transactivation events involved in the regulation of RNA metabolism of genes and later events involving ribosomal biogenesis, nucleotide and energy metabolism. Remarkably, while some of the later events persist at steady-state, many new transcriptional events appear that involve the regulation of the cell cycle, DNA replication and oxidative phosphorylation. The observation that genes involved in DNA replication and cell cycle regulation are actively transcribed at 48 hours is compatible with the entry of Myc activated P493 cells into S-phase by 48 hours [14].

Not only does ANRO reveal waves of transcriptional events associated with specific cellular functions following Myc induction, but the use of GSMA also allows the reconstruction of putative transcription factors that might be involved in regulating these waves of transcriptional events. Specifically, by clustering groups of genes that are enriched in their promoters for specific transcription factor motifs as a function of time after Myc induction, we could re-construct the putative transcriptional networks. The very early transcriptional events mimic those observed after serum stimulation of fibroblasts. A large middle wave of genes (1–6 hr) appears to be regulated by different transcription factors, most notable being Myc/Max, E2F and NFκB. This wave of genes is involved in ribosomal biogenesis, nucleotide metabolism and energy regulation. At a late, steady-state phase, a distinct transcriptionally active group of genes appears to be regulated by Myc-Max and E2F, although motifs for ARNT (HIF), USF (which has the same core consensus site as Myc) and YY1 are also represented in the promoters of these genes. These transcription factors are associated with genes involved in DNA replication and cell cycle regulation.

In addition to the ability to re-construct transcriptional events globally following Myc activation, ANRO also permits the identification of direct Myc target genes which could now be defined more precisely. ANRO adds the transactome or the missing component of evidence for transcriptional regulation through the direct measurement of nuclear run-on. The transactome completes the tripartite components, which include demonstration of physical binding of a transcription factor to its target genes by chromatin immunoprecipitation (ChIP-chip or ChIP-PET) and mRNA level changes (as determined by standard gene expression profiling with microarrays). Coupling these three components of measuring transcriptional regulation, we identified in the human P493-6 B cell model, a set of direct Myc targets that are involved in ribosomal biogenesis, nucleotide metabolism, energy metabolism and cell cycle progression. GSEA reveals a stem cell signature amongst the direct Myc up-regulated genes, suggesting the Myc maintains these cells in an undifferentiated stem cell-like state. These observations taken together further support the notion that Myc is a master transcription factor that participates in the regulation of genes in essentially all phases of the cell cycle to stimulate cell growth and proliferation.

Our studies also underscore the fact that Myc requires other transcription factors to regulate subsets of genes involved in specific cellular functions in different phases of the cell cycle. Early after Myc induction, genes involved in signaling and transcription are abundantly transcribed. Myc regulates subsequent events that induce genes involved in ribosomal biogenesis among other biosynthetic processes. In this regard, it is notable that ribosomal biogenesis has been proposed to be a primordial function of Myc during evolution [5]. While cells prepare for biosynthesis in the earlier cell cycle phases, genes involved in the regulation of cell proliferation persists at the late, steady-state phase with Myc and E2F playing a major role. In addition to new lessons learned about the Myc transcriptional network through the use of ANRO, we provide here a new experimental approach that should provide a better understanding of any transcriptional network downstream of known transcription factors. The application of ANRO to the study of oncogenic or stem cell transcription factors should rapidly enrich our knowledge of these important transcriptional networks.

## Materials and Methods

### Nuclei Isolation for *In Vitro* labeling of Nascent RNA

Collect 20×10^6^ cells by spinning 3–5 min at ∼216×g; Wash once with Cold PBS and spin again at the same speed; Carefully decant PBS, add 10 ml Cell Lysis Buffer (20 mM Tris-HCl, pH 7.5, 5 mM MgCl_2_, 20 mM NaCl, 100 mM Sucrose, 0.25% NP-40) to cell pellet; Sit on ice for ∼5 min with hand-mixing several times in between; Spin at ∼216×g, carefully decant and drain off supernatant; Add100 µl Nuclei Storage Buffer (50.0 mM Tris-HCl, pH 8.0, 5.0 mM MgCl_2_, 0.1 mM EDTA, 45% Glycerol) to resuspend each nuclear pellet.

### Labeling of Nascent RNA (*In Vitro* NRO Reaction

To above 100 µl nuclei, add 100 µl 2× NRO Reaction Buffer (300 mM KCl, 10 mM MgCl_2_, 2 mM of cold rATP, rCTP, rGTP and 1 mM Biotin-modified UTP, ribonuclease inhibitor such as Invitrogen RNaseOUT™ 100 Units); Incubate at 28°C for 25–30 min with constant mixing; (Optional: add cold rUTP to a final 1 mM, continue the incubation for another 5–10 min;) Add RNase-free DNase I (such as 100 Units of Roche DNase I), incubate 10 min at 37°C; Add 15 µl 3∶1 of Proteinase K (20 mg/ml) : 10% SDS; incubate 10 min at 37°C.

### Purification of Total Nuclear RNA

Various protocols can be used, here is a modified Qiagen RNeasy RNA Isolation Kit procedure. Add RLT Buffer (supplemented with 2-BME) and bring the volume up to ∼650 µl; Add 750 µl 100% Ethanol; Mix well and load onto RNeasy Column; Spin at Max Speed and discard flow through; Wash ONCE with 700 µl RW1 Buffer; Wash TWICE with Kit Wash Solution; After last wash, spin additional 2 min at Max Speed to remove trace wash solution; Transfer column to collection tubes, add 50–100 µl RNase-free H2O (prewarmed at 60°C) at the column center, let sit at room temperature for ∼2 min; Spin 2 min at Max speed to elute RNA; Quantify RNA amount.

### Preparation of Avidin-beads

Using magnet stand, sequentially wash Dynabeads® M-280 Streptavidin (30 µl per sample) with equal volume of 1×PBS, RNase-free Solution A (100 mM NaOH, 50 mM NaCl), RNase-free Solution B (100 mM NaCl), twice of each wash; Wash once with Dynal® kilobaseBINDER™ Binding Solution (supplemented with ribonuclease inhibitor such as Invitrogen RNaseOUT™ 100 Units per sample); Resuspend beads in equal volume of Dynal® kilobaseBINDER™ Binding Solution (30 µl per sample, supplemented with ribonuclease inhibitor as above), put on ice.

### Capture of Biotin-labeled RNA

Aliquot equal volume of total nuclear RNA (typically 5–10 µg in 30 µl of RNase-free H_2_O), denatured ∼3 min at 68°C and immediately put on ice; Add carefully into corresponding Beads tubes prepared above; Gently pipette up and down, rotate 3 hrs at ∼6 rpm at room temperature or ∼2 hrs at room temperature, then 4°C over night. Using magnet stand, sequentially wash RNA-bound Dynabeads® sequentially with RNase-free H_2_O six times (twice 600 µl, twice 400 µl, and twice 200 µl).

### 1^st^ and 2^nd^ Strand cDNA Synthesis

Various sources of reagents can be used, here is a modified procedure using Ambion TotalPrep RNA Amplification Kit. Resuspend above washed RNA-bound beads in 24 µl T7N_6_ Primer Solution (22 µl RNase-free H_2_O + 2 µl of 10 pmoles/µl T7N_6_); Denature bound RNA at 68°C 3 min with shaking; Rotate at room temperature 10 min for T7(N)_6_ annealing; Add 16 µl of 1^st^ Strand Mix (4 µl 10×1^st^ Strand Buffer, 8 µl dNTPs, 2 µl RNase Inhibitor, 2 µl ArrayScript); Incubate at 25, 28, 32, 37°C 1 min each, then 42°C 2 hrs (with constant mixing). To the above 40 µl 1^st^ strand reaction on beads, add RNase H Mix (10 µl RNase-free H_2_O, 6 µl of 10X 2^nd^ Strand Buffer, 2 µl of Invitrogen Random Primers at 3 µg/µl stock solution, 2 µl RNase H); Incubate 30 min at 37°C with rotation; 68°C 3 min to separate 1^st^ Strand cDNA completely from beads; Quick spin and immediately put on magnet; Transfer supernatants into new tubes; Let stand 5 min at room temperature for random primer annealing. To the above 60 µl RNase H Reaction, add 2^nd^ Strand Synthesis Mix (30 µl RNase-free H_2_O, 4 µl of 10×2^nd^ Strand Buffer, 4 µl dNTPs, 2 µl DNA Polymerase); Incubate 2 hrs at 37°C.

### cDNA Purification, cRNA Synthesis and cRNA Purification

Various sources of reagents can be used, here is a modified procedure using Ambion TotalPrep RNA Amplification Kit. Add 250 µl cDNA Binding Buffer to each sample, mix well and load onto cDNA Filter Cartridge; Spin 1 min at 10,000×g, discard flow-through; Wash once with 500 µl Wash Buffer, spin additional 2 min to remove residue solution and transfer cartridge to cDNA Elution Tube; Elute cDNA with 20 µl of H_2_O (55°C preheated). Add IVT (in vitro transcription) Mix (T7 10X× Reaction Buffer, T7 Enzyme Mix, Biotin-NTP Mix, 2.5 µl each), incubate 14 hrs at 37°C. Add 350 µl cRNA Binding Buffer and 250 µl of 100% Ethanol, mix well and load onto cRNA Filter Cartridge; Spin ∼1 min at 10,000×g, discard flow-through; Wash once with 500 µl Wash Buffer, spin additional 2 min to remove residue solution and transfer cartridge to cRNA Elution Tube; Elute cRNA with 100 µl of H_2_O .

### Cell Culture and Treatment

Human Jurkat E6-1 T cells and B Lymphoma P493-6 cells were cultured in RPMI-1640 Medium (GIBCO-BRL, Gaithersburg, MD) supplemented with 10% heat-inactivated FCS (Hyclone, Logan, UT) and antibiotics, and incubated in a humidified incubator (95% air and 5% CO_2_) at 37°C. Jurkat cells were stimulated with 40 ng/ml phorbol 12-myristate 13-acetate (PMA) and 1 µM ionomycin (Sigma, St. Louis, MO) (P+I). To induce the expression of Myc in P493 cells, cells were first cultured in 0.1 µg/ml tetracycline (tet) for 48 hr; then withdrew tet by changing new medium and cells were collected at 15 min, 30 min, 1 hr, 3 hr, 6 hr, and 48 hr following tet withdrawal.

### Array analysis

For Affymetrix Exon arrays, 10 µg nuclear run-on cRNA was converted to cDNA and labeled using the Affymetrix Whole Transcript (WT) Sense Target Labeling Assay (cat. no. 900670) according to the manufacturer's recommendations. Total RNA was labeled in a similar fashion using an input of 100 ng RNA without ribosomal RNA reduction. 5.5 ug of fragmented, labeled sense target DNA generated from either NRO or Total cRNA was hybridized to Affymetrix GeneChip Human Exon 1.0 ST Arrays using the Affymetrix FS450_0001 fluidics station protocol and quantitated using an Affymetrix 3000 7G scanner. Data was processed through Affymetrix GeneChip Operating Software (GCOS) Expression Console and further analyzed (for differential gene expression and alternative splicing) using software from JMP (JMP Genomics 3.0) and Exon and Gene Array Expression Analysis from Biotique-XRAY (Reno, NV). For Illumina arrays, biotin-labeled cRNA (750 ng total RNA-cRNA or 2 µg NRO-cRNA) was combined with hybridization buffer + formamide and hybridized (16 hr) at 55°C to Illumina's Sentrix HumanRef-8 Expression BeadChips (Illumina, San Diego, CA, Cat#11201828). After hybridization, the hybridization cartridge was disassembled and the beadchip slide array was placed in a 55°C high temperature wash followed by washing and blocking at room temperature. Bound biotinylated cRNA was stained with streptavidin-Cy3 and further washed. The dry beadchip was stored in a dark box until scanned. The beadchip slide was quantitated using Illumina's BeadStation 500GX Genetic Analysis Systems scanner. Preliminary analysis of the scanned data including monitoring of quality control metrics was performed using the Illumina BeadStudio software.

### Quantitative RT-PCR (QRT-PCR) Analysis

Reverse transcription was performed using either total RNA isolated from cells or cDNA and cRNA from NRO RNA (specific primer design: **GAPDH**, C-GTA AGGAGATGCTGCATTCGCC, 3-GCAACAATATCCACTTTACCAGAGTT, E-CCCTGACAACTCTTTTCATCTTCTAG , 5-GGTCTACATGGCAACTGTGAGGAG ,4-TGGGATTTCCATTGATGACAAGC, **CD69**, B-GCTCCAGCAAAGACTTTCACTGTAGC,2-ATAGAGAGATTACCAGTATATCTTGTATAACTACT, E-ACATGGTGCTACTCTTGCTGTCAT, 5-CTACATCTGAAATAGGTACAATGTTTG, D-AGTTCCTGTCCTGTGTGCTGTAATG) processed with Applied Biosystems (Foster City, CA) High-Capacity cDNA Archive kit first-strand synthesis system for RT-PCR according to the manufacturer's protocol. QRT-PCR amplifications were carried out in duplicate or triplicate on an ABI Prism® 7300 Sequence Detection System, using SybrGreen incorporation for detection. Relative gene expressions are calculated by using the 2^−ΔΔCT^ method. The normalized ΔCt value of each sample is calculated using GAPDH as an endogenous control gene. Fold change values are presented as average fold change = 2^−(average ΔΔCt)^ for genes in treated relative to control samples.

### Array Data Analysis

For Affymetrix Exon arrays, data was normalized by the RMA method [23] For Illumina arrays preliminary analysis of the scanned data was performed using Illumina BeadStudio software as described [24]. Any gene consistently below a background threshold level of D = .98 for all samples was eliminated from further analysis. Z-transformation for normalization was performed on each Illumina sample/array on a stand-alone basis [25]. Significant changes in gene expression for both Affymetrix and Illumina data were calculated by Z test [25]. Significant genelists were calculated by selecting genes which satisfied a significance threshold criteria of Z test p-values less than or equal to 0.001 (10^−3^), a false discovery rate less than or equal to 0.1, and either a fold change ±1.5 or greater, or a Z ratio values greater or less than ±3.0. For direct comparison of results between Affymetrix and Illumina array data sets (logratios) were joined on the basis of HUGO gene names after averaging of duplicate genes.

Hierarchical clustering was performed using the Cluster and TreeView software programs, developed at Stanford University [26]. The clustering algorithm was set to complete linkage clustering using the uncentered Pearson correlation.

### GSMA Gene Set analysis and Functional Annotation

Gene Set Matrix Analysis (GSMA) [24] was performed using the median differences for differentially expressed genes tested against genesets derived from a variety of sources (for example, the Pathway genelists were originally obtained from the Gene Set Enrichment Analysis (GSEA) website maintained by the Broad Institute @ (www.broad.mit.edu/gsea). PAGE calculations [27] were automatically calculated according to the formula: Z = (Sm−µ)*m^1/2^/*σ* where **Sm** is the median of the logratio values of genes for a given gene set and the size of the given gene set is **m**. The median of total logratio values (µ) and standard deviation of total logratio values (***σ***) of a given microarray data set were calculated for all genes between two experimental groups. Functional annotation was performed using the Database for Annotation, Visualization and Integrated Discovery (DAVID), NIAID/NIH [19].

## Supporting Information

Figure S1 ANRO transcripts are both full length and concordant with total RNA. Results from Affymetrix exon arrays illustrate congruent patterns of gene expression for the MYC regulated Protein Kinase, DNA-activated, catalytic polypeptide (PRKDC) gene in either (a) Array based-NRO or, (b) total RNA, at both the individual exon and the whole gene level. The PRKDC gene is encoded by a total of 91 exons and spans approximately 190 Kb on chromosome 22q.
(0.38 MB TIF)

Table S1 (0.03 MB DOC)

Table S2 (0.18 MB DOC)

Table S3 (0.17 MB DOC)

Table S4 (0.40 MB DOC)

Table S5 (0.04 MB DOC)
