# Supplementary figures and images for: Time-Dependent c-Myc Transactomes Mapped by Array-Based Nuclear Run-On Reveal Transcriptional Modules in Human B Cells
Source: PLoS One. 2010 Mar 15;5(3):e9691. doi: 10.1371/journal.pone.0009691 (PMC2837740; doi:10.1371/journal.pone.0009691)

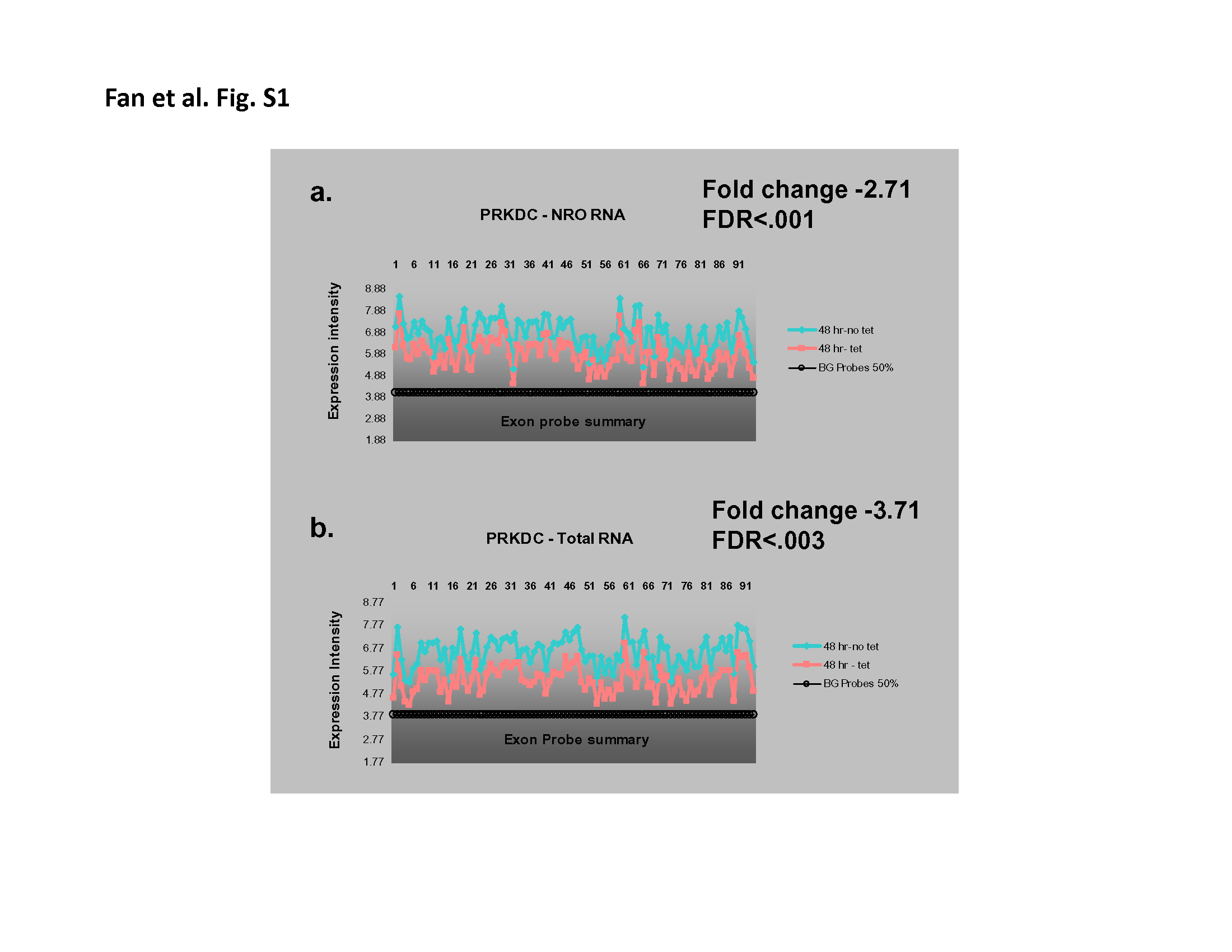

Supplement: Figure S1 — ANRO transcripts are both full length and concordant with total RNA. Results from Affymetrix exon arrays illustrate congruent patterns of gene expression for the MYC regulated Protein Kinase, DNA-activated, catalytic polypeptide (PRKDC) gene in either (a) Array based-NRO or, (b) total RNA, at both the individual exon and the whole gene level. The PRKDC gene is encoded by a total of 91 exons and spans approximately 190 Kb on chromosome 22q. (0.38 MB TIF) [file pone.0009691.s001.tif]
