# Supplementary material for: Time-Dependent c-Myc Transactomes Mapped by Array-Based Nuclear Run-On Reveal Transcriptional Modules in Human B Cells
Source: PLoS One. 2010 Mar 15;5(3):e9691. doi: 10.1371/journal.pone.0009691 (PMC2837740; doi:10.1371/journal.pone.0009691)
Supplement: Table S1 — (0.03 MB DOC) [file pone.0009691.s002.doc]

Primer PCR Product PCR Beads-bound NRO Illumina Affymetrix

Transcript Ensembl Transcript ID position length (bp) Target nascent RNA cRNA Ref8 Array Exon Array

Primer sets Primer Sequence (5' ---- 3') QPCR Fold QPCR Fold Zratio Fold

ANXA1-A/1 CAT GTG CAA TGG AAA GAA TCT GTT ANXA1 ENST00000376912 Intron 3-4: 75nt-99nt 142 ANXA1 262.44 1888.93 3.33 4.98

GAG CCA GTA ATA TCT GCT GTA ACA ATA GAG Intron 3-4: 216nt-187nt

ANXA1-C/3 AAT CTA GTA TAG TAC CTG TCA ATC AGT GG ANXA1 ENST00000376912 Intron 5-6: 80nt-108nt 136 ANXA1 267.55 425.58

TGA CTC ATA TAG TGA CAT GAC ATG GAA G Intron 5-6: 215nt-188nt

DUSP1-A/1 AGG GAG AAT ATA GAA AGT GAC CTG C DUSP1 ENST00000239223 Intron 2-3: 65nt-89nt 132 DUSP1 1.96 3.70 3.21 2.96

CAG CAA GTT CAT TTC CGT AGA GCC T Intron 2-3: 196nt-172nt

DUSP1-B/2 ACA AAG TTG AAT TGA CTG GTC TGA GAT C DUSP1 ENST00000239223 Intron 3-4: 197nt-124nt 175 DUSP1 5.04 3.50

CTG AGA AAG GTC ATG TGT GTC TTG TCT Intron 3-4: 371nt-345nt

DUSP2-A/1 ACA CTT TTA TAC GTG TGT GTG TTG G DUSP2 ENST00000288943 Intron 2-3: 111nt-135nt 146 DUSP2 35.67 25.50 3.00 3.16

TCT CAG GAA TGT GCG GAG GAC AC Intron 2-3: 256nt-234nt

DUSP2-B/2 GCA CAT TCC GTC TGA CAC CAC TC DUSP2 ENST00000288943 Intron 3-4: 77nt-99nt 179 DUSP2 15.75 19.08

CGC TGC TTA ACG AAG TCA AAG GC Exon-4: 139nt-117nt

EGR1-A/1 AGC AGC AGC AGC AGC ACC TTC AAC EGR1 ENST00000239938 Exon-1: 508nt-531nt 168 EGR1 35.82 24.31 4.78 4.84

CGG CTC TCA TTC TAA GAT CCA GGA G Intron 1-2: 98nt-74nt

EGR1-B/2 CAG TAA GAC TTG CCT TGC CTT GCT T EGR1 ENST00000239938 Intron 1-2: 298nt-322nt 205 EGR1 129.06 29.90

AGC TGA CGC AAA TAC GGA GAA TCC Intron 1-2: 502nt-479nt

EGR2-A/1 GGG AAT CGC AGG AGA GGA TTG G EGR2 ENST00000242480 Intron 1-2: 72nt-93nt 141 EGR2 152.36 45.21 3.35 4.82

CGG TTT GCT GGC GAC CTG GCG Intron 1-2: 212nt-192nt

EGR2-B/2 CTT GGC GAT GCT GTG GAA AGT CGT EGR2 ENST00000242480 Intron 1-2: 667nt-690nt 154 EGR2 100.26 110.11

GAT GTA GAG GAA GGA GTC CGT AGG TT Intron 1-2: 820nt-795nt

FOS-A/1 GCT TGC CAT AGT AAG AAT TGG TTC C FOS ENST00000303562 Intron 1-2: 174nt-198nt 135 FOS 16.22 26.86 5.36 4.83

GCT CTA GTT AGC GAG TTT GTG CTT G Intron 1-2: 308nt-284nt

FOSB-A/1 CTC TCA TTA ACC ACT GCG TCA CGG T FOSB ENST00000353609 Intron 1-2: 1724nt-1749nt 172 FOSB 75.11 44.21 5.39 5.01

ACA GGC GTA GAC ACA CAA ACA AAG AC Intron 1-2: 1895nt-1869nt

FOSB-B/2 CAT AAC AGT GGC TCA TGC CTG TGA TC FOSB ENST00000353609 Intron 3-4: 651nt-676nt 125 FOSB 119.12 16.61

TTT TTT CTT GGT AGA GAC AAG TCC TCC Intron 3-4: 775nt-749nt

IL8-B/2 AGT CGT TCA ATG TTG TCA GTT ATG ACT GTT IL8 ENST00000401931 Intron 1-2: 613nt-642nt 164 IL8 1804.21 318.56 2.62 2.60

CAC AGC TCT GCC AGC TAC TTC CTT TCT Intron 1-2: 776nt-750nt

IL8-C/3 CAA AGA ACT GAG AGT GAT TGA GAG TGG AC IL8 ENST00000401931 Exon-2: 83nt-111nt 159 IL8 2578.74 98.92

CAT TTA CTA CTG TAA TCC TAA CAC CTG GAA C Intron 2-3: 105nt-76nt

TNFAIP3-C/3 GCA TCT GAT GGA CTA GGT CAC ATG AAT TNFAIP3 ENST00000237289 Intron 3-4: 84nt-110nt 152 TNFAIP3 24.25 45.90 3.38 4.17

AGA CTC GCT GCT ACA GGA ACG GAT Intron 3-4: 235nt-212nt

TNFAIP3-D/4 CAC ATC TAT AAC TAG ACA CTG AAA CAT CAG G/TNFAIP3 ENST00000237289 Intron 5-6: 21nt-51nt 130 TNFAIP3 45.96 80.40

GCA TTA CTC TTA CTA ACC AAG CAA GTC AC Intron 5-6: 150nt-122nt

GAPDH-C/3 GTA AGG AGA TGC TGC ATT CGC C GAPDH ENST00000229239 Intron2-3: 1526nt - 1547nt 173 GAPDH

GCA ACA ATA TCC ACT TTA CCA GAG TT Exon3: 66nt - 41nt

CD69-B/2 GCT CCA GCA AAG ACT TTC ACT GTA GC CD69 ENST00000228434 Exon1: 14nt - 39nt 223 CD69 See Fig. 2

ATA GAG AGA TTA CCA GTA TAT CTT GTA TAA CTA CT Intron1-2: 91nt - 56nt

CD69-E/5 ACA TGG TGC TAC TCT TGC TGT CAT CD69 ENST00000228434 Exon3: 158nt - 181nt 166 CD69 See Fig. 2

CTA CAT CTG AAA TAG GTA CAA TGT TTG Intron3-4: 123nt - 97nt

CD69-D/5 AGT TCC TGT CCT GTG TGC TGT AAT G CD69 ENST00000228434 Exon2: 50nt - 74nt 1434 CD69 See Fig. 2

CTA CAT CTG AAA TAG GTA CAA TGT TTG Intron3-4: 123nt - 97nt
