# Supplementary material for: Time-Dependent c-Myc Transactomes Mapped by Array-Based Nuclear Run-On Reveal Transcriptional Modules in Human B Cells
Source: PLoS One. 2010 Mar 15;5(3):e9691. doi: 10.1371/journal.pone.0009691 (PMC2837740; doi:10.1371/journal.pone.0009691)
Supplement: Table S2 — (0.18 MB DOC) [file pone.0009691.s003.doc]

**TABLE S2. Nuclear Run-on Up-regulated Genes – Time Course**

**15 minutes**

Symbol NRO 15'

MYC 2.56

FOS 2.19

EGR1 2.17

BCL2L11 1.31

CD69 0.81

RGS2 0.67

DUSP1 0.67

DUSP2 0.64

EIF1 0.62

IL17RE 0.61

LIPT1 0.6

GLS 0.56

RDH10 0.55

ABRA 0.55

RPS17 0.53

YWHAB 0.53

CEBPZ 0.52

ATF4 0.51

GARNL3 0.51

AK5 0.51

IGSF4B 0.51

AYP1 0.51

IL24 0.51

CCDC59 0.5

OR4N5 0.5

**30 minutes**

Symbol NRO 30'-0

NR4A3 3.08

BCL2L11 2.56

NR4A3 2.19

NR4A3 2.17

DUSP5 1.31

NR4A1 1.09

FTH1 1.04

NR4A1 0.95

NR4A2 0.89

CD248 0.85

EBI2 0.82

SOCS3 0.81

SNF1LK 0.8

HERPUD1 0.8

HLA-DPB1 0.78

PTP4A1 0.77

FOSB 0.77

SYCE1 0.75

ZFP36 0.75

SYNGAP1 0.75

TBX3 0.71

CD74 0.7

POU6F1 0.7

FMNL3 0.69

CSPG3 0.69

ARRDC3 0.69

SLC2A3 0.68

ALG2 0.67

RAD17 0.67

PIM2 0.67

PLEKHB2 0.67

SLC2A14 0.65

TAF9L 0.65

HAS3 0.65

ZNF2 0.65

USP11 0.64

HMGB1 0.64

PPA2 0.64

TM2D3 0.64

DEDD 0.63

VMD2 0.62

IGFL3 0.62

LSM16 0.6

ARL5B 0.6

SSBP3 0.59

PRKAR1A 0.59

SIRT3 0.57

RPS16 0.56

ZNF92 0.55

JUND 0.54

TRIM4 0.53

MANBAL 0.53

EIF5 0.52

PRIM2A 0.52

SLC30A1 0.52

TAF11 0.52

TFIP11 0.51

RPL36AL 0.51

MID1IP1 0.51

TSC22D3 0.5

OR1Q1 0.5

ZMAT5 0.5

HSA9761 0.5

CDC14B 0.5

LRRIQ2 0.5

RPL14 0.5

**1 hour**

Symbol NRO 1hr

PDE4D 1.38

TA-NFKBH 1.2

SPIRE2 1.19

HPSE2 1.13

KHSRP 0.96

BCL3 0.88

TIMP1 0.85

TGIF 0.85

PDE4B 0.81

KLF10 0.79

HNRPA3 0.79

PERLD1 0.77

GPR132 0.75

CD83 0.74

MYADM 0.74

YBX1 0.72

SKIL 0.72

PTER 0.71

NALP2 0.7

LNK 0.7

ZNF587 0.7

OR52I1 0.7

VPS13B 0.7

CRSP7 0.69

ENO1 0.69

PKM2 0.68

ATAD2 0.68

GSTM2 0.67

ATP2B4 0.67

RASGEF1A 0.66

SH3KBP1 0.66

CLCF1 0.65

HES1 0.64

ELOF1 0.64

KCTD17 0.64

GM2A 0.63

DGUOK 0.63

PRIC285 0.63

KREMEN2 0.63

EXOSC10 0.63

LSP1 0.63

LRP5L 0.62

SKI 0.61

IDS 0.61

CHEK2 0.61

TCEA3 0.6

EFEMP2 0.6

IVD 0.6

F8A1 0.6

GALNAC4S 0.6

MAP4 0.59

PIP5K2A 0.59

DENND1C 0.59

SIPA1L1 0.59

DCTN6 0.59

PDIA6 0.59

LRRC25 0.58

ATP5D 0.58

XCR1 0.58

IDS 0.58

ALDOA 0.58

CUGBP1 0.58

LIMK1 0.58

DDX39 0.58

FOLR1 0.57

SMAD7 0.57

GADD45B 0.57

ZC3H12A 0.57

HLA-DQB1 0.57

PXK 0.57

SRPR 0.57

FCGR3A 0.57

MPHOSPH10 0.57

BBS1 0.57

N4BP3 0.57

MDS1 0.57

TRIM41 0.57

BAT3 0.56

DOT1L 0.56

BCOR 0.56

RGC32 0.56

BCORL2 0.56

PABPC4 0.56

LGALS7 0.56

DDI2 0.55

RAB39 0.55

OXA1L 0.55

STIM1 0.55

MAN2B1 0.55

BRD4 0.55

B4GALT1 0.55

MFHAS1 0.55

OR5H1 0.55

JAK3 0.55

YWHAZ 0.55

CD96 0.55

SLC10A3 0.54

ZBTB5 0.54

RPL4 0.54

TCF2 0.54

M-RIP 0.54

CUTL1 0.54

MICB 0.54

LRRC33 0.54

ZNF554 0.53

EPIM 0.53

PRF1 0.53

NIN 0.53

LELP1 0.53

NR1H2 0.53

BSG 0.53

DCAKD 0.53

UROS 0.53

LRP2BP 0.53

GART 0.53

PTPN14 0.53

RAPGEF1 0.53

ELK4 0.53

TAPBP 0.52

TPR 0.52

XPC 0.52

ALDH16A1 0.52

DIP 0.52

SRRM2 0.52

TRIM54 0.52

HAP1 0.52

SLC7A11 0.52

F3 0.52

CIZ1 0.52

WDR48 0.52

ADD2 0.51

PDLIM7 0.51

SON 0.51

SURF5 0.51

CDC2L2 0.51

S100A10 0.51

BTNL9 0.51

RAB2B 0.51

OR4Q3 0.51

COPB2 0.51

PHF19 0.51

ZNF250 0.51

PIK3R1 0.51

CLN6 0.51

MYST4 0.51

ECH1 0.5

MRPS22 0.5

MYH9 0.5

ZC3HAV1 0.5

PRKCBP1 0.5

HRH4 0.5

MSRA 0.5

PTPN1 0.5

NCAM2 0.5

BACH1 0.5

CD19 0.5

GABPB2 0.5

INVS 0.5

YPEL1 0.5

PILRA 0.5

IFNAR2 0.5

SORT1 0.5

ATAD3A 0.5

GGT1 0.5

**3 hours**

Symbol NRO 3hr

EML5 1.64

SAMSN1 1.6

TM6SF1 1.53

OR5B21 1.51

GREB1 1.46

EIF2C1 1.2

WDR12 1.15

DAB2IP 1.13

MPZL1 1.12

LGMN 1.1

PLXNA1 1.1

MUM1 1.08

SBF1 1.08

SAMD1 1.06

ANKRD41 1.04

SLC2A5 1.04

LIX1L 1.01

ADA 1.01

ABI3 1

SRM 0.99

MGAT4A 0.98

PCBD1 0.97

RUNX2 0.97

ACSL1 0.96

CMTM7 0.96

CUEDC1 0.96

STOM 0.96

RRS1 0.94

FSTL3 0.93

CLIC3 0.93

PLEC1 0.93

LPIN1 0.93

NRP2 0.93

ITGB1 0.92

CTNND1 0.91

TNFSF4 0.91

XTP3TPA 0.9

SLC35F2 0.9

AFMID 0.9

MPP6 0.88

A4GALT 0.87

CD6 0.87

UCK2 0.87

EXOSC5 0.86

ABR 0.85

MARS2 0.84

RAI1 0.84

BCAS4 0.84

FSCN1 0.82

ERN1 0.82

SYT15 0.82

STK17A 0.81

RSL1D1 0.81

DDX10 0.8

XKR5 0.8

SDCCAG3 0.79

BCOR 0.79

ST8SIA5 0.79

GLT1D1 0.79

SPATA2 0.79

SPRED2 0.79

SMAD6 0.78

WNK1 0.78

FMNL3 0.78

MARCKSL1 0.78

ASGR2 0.77

NEFH 0.77

CGI-69 0.77

FSD1CL 0.77

PER2 0.77

BRI3BP 0.77

RUNX3 0.76

ZA20D1 0.76

HSPA8 0.76

CIRH1A 0.76

PKP4 0.76

DDX21 0.75

WDR50 0.75

HEATR1 0.75

TIMM23 0.75

SMG5 0.75

RNF122 0.74

KALRN 0.74

LPIN2 0.73

USP51 0.73

ATPAF2 0.73

OGFRL1 0.73

FAM48A 0.73

KRTHB4 0.73

ATPBD4 0.73

ASCC3 0.73

RFFL 0.72

NOL5A 0.72

GNL3 0.72

HSP90AB1 0.72

KCNV2 0.72

OGG1 0.72

EIF2C3 0.71

FABP5 0.71

TFB2M 0.71

SLC41A2 0.71

TXLNA 0.7

PGLYRP4 0.7

RLTPR 0.7

INPP5D 0.7

FCHO1 0.7

GBP6 0.7

GNA11 0.7

PRMT5 0.7

FBXL19 0.69

FCRL3 0.69

HRIHFB2122 0.69

FCRL6 0.69

SOCS2 0.69

TLE3 0.68

CYLC2 0.68

PTPN9 0.68

PGD 0.68

TSNAXIP1 0.68

LYAR 0.68

HMG1L1 0.68

NXN 0.68

NEU4 0.67

CRB2 0.67

WDR4 0.67

HPS3 0.67

SERPINA10 0.67

NUDC 0.67

PPP1R16B 0.67

NLGN2 0.67

HACE1 0.67

PFDN2 0.66

FOXG1B 0.66

TRIM44 0.66

SLC39A7 0.66

XYLB 0.66

CRELD2 0.66

BXDC1 0.66

NT5DC2 0.66

SPPL2B 0.66

WDR4 0.66

EIF2C3 0.66

TNFRSF4 0.66

FLVCR 0.66

SLC27A6 0.65

PPAT 0.65

ENG 0.65

RAP80 0.65

SLC35D1 0.65

ODZ1 0.65

KIF21A 0.65

MPZL1 0.65

ADMR 0.65

SCD 0.65

SELPLG 0.65

GSPT1 0.64

NFATC2 0.64

XYLT1 0.64

PHF2 0.64

GCNT4 0.64

SLC22A5 0.64

PIAS2 0.64

HSPA8 0.64

AP2A1 0.64

ATAD3B 0.64

SEC8L1 0.64

CECR1 0.64

ACP5 0.64

NUMBL 0.64

BDP1 0.64

NP 0.64

RPS6KA1 0.64

VMD2L3 0.63

ABCA12 0.63

XKR6 0.63

TRIM43 0.63

CD82 0.63

CEP170 0.63

OGG1 0.63

JTB 0.63

CCR7 0.63

DNMT1 0.63

MAPK1 0.63

HSF2 0.63

AHDC1 0.62

ARFGAP3 0.62

DCUN1D5 0.62

ISYNA1 0.62

AGMAT 0.62

AGPAT3 0.62

IBRDC3 0.62

ACCN4 0.62

CENTG3 0.62

WTAP 0.62

FGG 0.62

CCL7 0.62

DLG5 0.62

MAGED2 0.62

DNM2 0.62

SNCB 0.62

SPG21 0.62

AHSA1 0.61

GLIPR1 0.61

TMEM58 0.61

TTF2 0.61

KCNC3 0.61

SSH1 0.61

KRTAP5-1 0.61

GTF2I 0.61

AK2 0.61

SDC2 0.61

BASP1 0.61

ANGPTL6 0.61

ARF3 0.61

NBPF15 0.61

SOX30 0.61

NT5DC3 0.6

OR2T35 0.6

PPM1G 0.6

KRT3 0.6

NOMO1 0.6

PRDM7 0.6

PPARGC1B 0.6

IARS 0.6

COQ4 0.6

KSR1 0.6

ROM1 0.6

XPOT 0.6

CHRNB3 0.6

PIGM 0.59

MAGOH 0.59

ITPR3 0.59

PLXNB1 0.59

AQP12A 0.59

SESN3 0.59

NUP43 0.59

FGF6 0.59

APOC3 0.59

CMIP 0.59

PMM2 0.59

GAS2L1 0.59

CREB5 0.58

GOLGA7 0.58

NARG1L 0.58

EML1 0.58

SERINC5 0.58

TGFBRAP1 0.58

PHF13 0.58

SUPT5H 0.58

DKC1 0.58

FKBP11 0.58

ODF2 0.58

TBL1X 0.58

KTN1 0.58

ANKRD11 0.58

USP13 0.58

FCRL3 0.58

ZNF407 0.58

CDH9 0.58

GTF2A1 0.57

CYP2B6 0.57

PA2G4 0.57

TRPM6 0.57

VDAC1 0.57

ADAMTSL2 0.57

FOSL2 0.57

CTPS 0.57

SSRP1 0.57

SRD5A2L 0.57

TPCN1 0.57

ARHGEF17 0.57

CEECAM1 0.57

C3 0.57

PITRM1 0.57

POLA 0.57

TRIM50B 0.57

ORC2L 0.57

ATP1A1 0.57

PPBP 0.57

ARHGDIG 0.57

IL7R 0.57

LAPTM4A 0.57

ZNF417 0.56

CESK1 0.56

HM13 0.56

HSPD1 0.56

RPIP8 0.56

MUTYH 0.56

SLC3A2 0.56

UCHL3 0.56

TMED10 0.56

PDCD6 0.56

CAPN6 0.56

PCDHGB6 0.56

DGKB 0.56

OR56B1 0.56

GEMIN5 0.56

PEBP1 0.55

ABI2 0.55

PPP2CB 0.55

SDCCAG8 0.55

GLUD1 0.55

ITGB3 0.55

CALML4 0.55

AHCYL1 0.55

MAPK15 0.55

HDGF 0.55

STATIP1 0.55

NUP98 0.55

XRCC6 0.55

CNOT10 0.55

KLHL25 0.55

SLC5A6 0.55

GPATC1 0.55

PHF17 0.55

SQLE 0.55

SLC35B1 0.55

MAPKAPK2 0.55

EFTUD2 0.55

HNRPL 0.55

D15Wsu75e 0.55

KLHL26 0.54

MAPRE2 0.54

HNRPUL1 0.54

EIF2C2 0.54

UBAP2 0.54

AKR1B1 0.54

ANP32A 0.54

MAP4 0.54

DNASE1L1 0.54

EBI3 0.54

RAB5C 0.54

RABEPK 0.54

TAAR2 0.54

HSPC023 0.54

PPP1R16A 0.54

ABCE1 0.54

ANK2 0.54

PHF16 0.54

NUDT15 0.54

ZNF585A 0.53

PHB2 0.53

GCET2 0.53

PDE4DIP 0.53

KCNH3 0.53

RAB27A 0.53

ITGAV 0.53

TEGT 0.53

SURF4 0.53

TGM5 0.53

PPA1 0.53

CBX5 0.53

KBTBD6 0.53

PSMB3 0.53

SCFD2 0.53

NDUFB2 0.52

NARG1L 0.52

KCNK3 0.52

MLLT6 0.52

PDCD11 0.52

CNR1 0.52

YTHDC1 0.52

SEPW1 0.52

COPB 0.52

NT5DC3 0.52

MRPS26 0.52

SYT15 0.52

RASSF3 0.52

ELSPBP1 0.52

ACAD9 0.52

EIF3S4 0.52

CSRP2BP 0.52

FAM40A 0.52

MAX 0.52

OR10G9 0.52

UTP14A 0.52

UNQ846 0.51

DNAH17 0.51

PDZD6 0.51

GRINA 0.51

ATP2A2 0.51

MOSPD3 0.51

SLC39A6 0.51

EHMT1 0.51

TULP2 0.51

ZNF485 0.51

MTRF1 0.51

MKNK2 0.51

PPIL5 0.51

BLVRA 0.51

NEO1 0.51

DIPA 0.5

WDR32 0.5

ZNF297 0.5

ACY3 0.5

VHL 0.5

TRA2A 0.5

RCL1 0.5

CACNG6 0.5

RNMTL1 0.5

SYNGR3 0.5

GLP1R 0.5

ROCK2 0.5

BRP44L 0.5

RAB11FIP4 0.5

ANXA5 0.5

EPM2AIP1 0.5

TSPYL6 0.5

RANGAP1 0.5

CXCL1 0.5

CAPZB 0.5

JAGN1 0.5

SLC19A2 0.5

THRAP4 0.5

ALMS1 0.5

PFAS 0.5

**6 hours**

Symbol NRO 6hr

SLC4A7 1.23

MATR3 1.22

POLR3G 1.05

PAICS 1.05

PRR5 1.03

NME1 1.01

TSEN2 1

CABC1 0.96

GLYATL2 0.93

HSPE1 0.92

ZNF239 0.9

KCNQ5 0.88

BCAT1 0.88

PRNP 0.87

NOLC1 0.85

CNNM1 0.85

DSCR2 0.84

PLXDC2 0.84

DSCR2 0.84

FJX1 0.83

WDR35 0.83

TMEM97 0.81

AICDA 0.81

LEF1 0.81

GUCY1A3 0.81

MTAP 0.8

PUS7 0.8

LRIG3 0.8

FBXO31 0.8

EBNA1BP2 0.8

TIGA1 0.79

IFRD2 0.78

WDR3 0.78

EXOSC2 0.77

TRAP1 0.76

SETD6 0.75

LASS6 0.74

GART 0.72

MRPL3 0.72

USP31 0.72

TRUB2 0.72

NCL 0.7

RCC1 0.7

ADK 0.7

CPXM2 0.7

POLR1C 0.69

CGI-96 0.69

CSE1L 0.69

UBE2G2 0.69

POLR1C 0.69

TMEM48 0.68

DDX18 0.68

DDX31 0.67

NLN 0.67

WDR21A 0.67

FAM86A 0.67

CLEC2D 0.67

SUPV3L1 0.66

RPIA 0.66

LDHB 0.65

DDX31 0.65

NAT10 0.64

IRF8 0.64

JAG2 0.64

ACAT1 0.64

AKAP1 0.63

MKI67IP 0.63

MINA 0.63

TSR1 0.63

TOMM40 0.63

MRPL24 0.63

USP36 0.63

PPRC1 0.62

EIF3S1 0.62

MDN1 0.62

DDN 0.62

GRPEL1 0.62

PDIA5 0.62

SLC7A1 0.62

MRPS25 0.61

ZNF330 0.61

ZNF568 0.61

COL24A1 0.61

TXNDC5 0.6

IMPACT 0.59

MINA 0.59

BRIP1 0.59

CSDA 0.59

BXDC2 0.59

SFRS2 0.59

EIF2S3 0.59

EEF1E1 0.59

NOL6 0.59

NDUFC2 0.58

ATIC 0.58

METAP1 0.58

LRP8 0.58

ELMO1 0.58

AHCY 0.58

LARS 0.58

PTCD2 0.58

HSPC111 0.58

CPNE7 0.57

PRDX1 0.57

GPATC4 0.57

SFXN2 0.57

MRPL36 0.56

SNRPF 0.56

BOP1 0.56

GEMIN4 0.56

DNAJC12 0.56

ME2 0.56

CLDN14 0.56

SRFBP1 0.56

SSB 0.56

NOL8 0.56

LARP2 0.56

DARS 0.55

CSTF3 0.55

LENG9 0.55

REXO2 0.55

SULT1A1 0.55

ZC3H8 0.55

UTP20 0.55

IL12A 0.55

PPP2R3B 0.54

PDSS1 0.54

NUP35 0.54

GLO1 0.54

ZNFN1A2 0.54

EIF1AX 0.54

PHB 0.54

RSAFD1 0.53

TMEM68 0.53

SURF6 0.53

HK2 0.53

TSFM 0.53

C1QBP 0.53

PAPD1 0.53

APEX1 0.53

GTPBP6 0.53

RPS23 0.53

ZNF528 0.52

AARSD1 0.52

YWHAG 0.52

PCDHB16 0.52

RGS16 0.52

IVNS1ABP 0.52

NPDC1 0.52

ALDH1B1 0.52

LARP2 0.52

POLR3H 0.52

DPH2 0.51

TBRG4 0.51

EHBP1 0.51

CHORDC1 0.51

POLRMT 0.51

DNAJC12 0.51

FAM86B1 0.51

UTP11L 0.51

ZNF613 0.51

CANX 0.51

POLD2 0.51

DSCR8 0.51

PRAF1 0.5

CHRM2 0.5

CACYBP 0.5

LAPTM4B 0.5

GNL3 0.5

RAP1GA1 0.5

PAK1IP1 0.5

TRIM61 0.5

FASN 0.5

**48 hours**

Symbol NRO 48hr

GAL 2.3

RNU3IP2 2.26

SLC7A2 2.14

TEAD4 1.96

MATK 1.88

SLC7A5 1.87

RBM9 1.84

BZW2 1.83

CCDC34 1.81

PHGDH 1.8

mimitin 1.76

RANBP1 1.76

NOLA2 1.72

HNRPA1 1.72

OGDHL 1.71

IMPDH2 1.7

MRPL12 1.69

RPS21 1.66

MCM4 1.64

HNRPAB 1.64

CAMKV 1.63

Pfs2 1.62

CAD 1.61

SLC2A4RG 1.61

TXN 1.6

ZMYND19 1.59

RUVBL2 1.59

FBL 1.59

DHX33 1.57

EPRS 1.55

MRPS2 1.54

UHRF1 1.53

ASS 1.52

SRXN1 1.51

GCAT 1.51

MRPL4 1.51

PIGW 1.5

CHTF18 1.5

TEX2 1.49

CENPF 1.48

CKB 1.47

GAJ 1.47

CCT2 1.47

BCS1L 1.46

THOC4 1.45

TUFM 1.45

METRN 1.45

NTHL1 1.43

TKT 1.43

KLHL23 1.43

TIMM8A 1.42

GLRX5 1.41

GLOXD1 1.4

PBK 1.39

CKS1B 1.39

PTRH1 1.38

RANBP5 1.37

CCRN4L 1.37

CDC25A 1.36

RBBP8 1.36

HSPA9B 1.36

RPS15 1.35

SRPK1 1.35

CDC20 1.34

NOLA1 1.33

EBPL 1.33

VARS 1.33

HADHSC 1.33

PDCD5 1.33

RPLP0 1.32

FAH 1.32

PRKAR1B 1.32

SLC43A3 1.31

CHRNA5 1.31

RRM1 1.31

APRT 1.31

MRPS17 1.31

CDC45L 1.31

MCM2 1.31

UBE2C 1.3

ALDH5A1 1.3

GNPNAT1 1.3

FOXN4 1.29

KIF11 1.29

PRDX4 1.29

KISS1R 1.29

GPSM1 1.29

STRA13 1.28

POP7 1.28

DTYMK 1.28

CDCA7 1.28

UIP1 1.28

CTNNAL1 1.28

CKS2 1.28

ALKBH2 1.28

ST7 1.27

SLC9A3R2 1.27

NME2 1.27

NDUFS5 1.27

TMEM38B 1.27

NDUFB6 1.26

JTV1 1.26

H2AFZ 1.26

APP 1.26

PKMYT1 1.26

HNRPA1 1.25

GPR30 1.25

AMD1 1.25

KIF22 1.25

MTHFD1L 1.25

CDK4 1.24

DDX48 1.24

PPP2R3B 1.24

RPL22 1.24

HPRT1 1.24

MRPL2 1.23

HRSP12 1.23

GMPS 1.23

TRMT1 1.23

SOD1 1.22

FXN 1.22

MRPL34 1.22

RPL29 1.22

POLR2I 1.22

NDUFAB1 1.21

HIST1H4L 1.21

ODC1 1.21

MRPL21 1.21

MIPEP 1.21

MFSD3 1.2

GTPBP4 1.2

SCML1 1.2

MAD2L1 1.2

CECR5 1.2

TBRG4 1.2

CHCHD4 1.2

CCT7 1.19

GMNN 1.19

POLR2F 1.19

CGI-115 1.19

CCNB1 1.19

IMP4 1.18

BYSL 1.18

DEPDC1B 1.17

CYC1 1.17

PCCB 1.17

WDR46 1.17

KIF15 1.16

NIFIE14 1.16

CCT6A 1.16

TRIP13 1.16

ADCY3 1.16

CENPE 1.15

LRRC14 1.15

GARS 1.15

BOLA3 1.15

GOT2 1.15

PRSS15 1.14

FH 1.14

PRDX6 1.14

MDH2 1.14

MECR 1.14

QP-C 1.13

TUBG1 1.13

WHSC1 1.13

CCNA2 1.13

RPL13A 1.13

GOLPH2 1.13

AMD1 1.13

RFC3 1.13

HADH2 1.13

CDC2 1.12

RFC3 1.12

NUP155 1.12

MRPS28 1.12

MTHFD2 1.12

POLD1 1.12

HIST1H2BM 1.12

ENTPD6 1.12

GMDS 1.12

MCM10 1.12

TOP1MT 1.12

NME1-NME2 1.11

TUBB3 1.11

STOML2 1.11

SETMAR 1.11

SLC38A5 1.11

UQCRC1 1.11

TRA16 1.11

NSBP1 1.11

TUBA6 1.1

KARS 1.1

LMNB1 1.1

PODXL2 1.1

SDHB 1.1

ACN9 1.09

RUVBL1 1.09

HELLS 1.09

PRPF31 1.09

TIMM44 1.08

DLG7 1.08

PDDC1 1.07

KCNJ12 1.07

HBLD2 1.07

PELP1 1.07

DECR2 1.07

CDCA2 1.07

LRP16 1.07

CDT1 1.07

PMPCA 1.07

GPHN 1.07

MRPS15 1.07

Magmas 1.07

FADS1 1.06

MRPS24 1.06

PRMT1 1.06

NUDCD2 1.06

MASA 1.06

RAD51 1.06

DNAJC11 1.06

APITD1 1.06

DDX11 1.05

WDHD1 1.05

SHFM1 1.05

MGST1 1.05

MCCC2 1.05

CHCHD6 1.05

ANP32B 1.04

MRPL45 1.04

FARSLA 1.04

SNX5 1.04

LSM4 1.04

ATP5J 1.04

CDCA7 1.04

MSH2 1.04

LTA4H 1.04

CFD 1.04

MRPL23 1.04

CDKN3 1.04

TACC3 1.04

EIF3S9 1.03

OAT 1.03

OGFOD1 1.03

ACY1 1.03

HMBS 1.03

GPD1L 1.03

CRY1 1.03

PNPO 1.03

CETN2 1.03

MRPL37 1.02

BM88 1.02

ATP5J2 1.02

ENDOG 1.02

GLDC 1.02

LSMD1 1.02

BTG3 1.02

PGRMC1 1.02

PRPS1 1.02

GPR30 1.02

SLC16A11 1.02

K-ALPHA-1 1.01

SLBP 1.01

PRC1 1.01

ATP5B 1.01

HMMR 1.01

LSS 1.01

FOXRED1 1.01

MCM3 1.01

IL32 1.01

IPO7 1.01

MRPS9 1

POLE2 1

RHBDD3 1

FIGNL1 1

POLR2H 1

E2F1 1

PTRH2 0.99

CREG1 0.99

BUB1 0.99

CHCHD2 0.99

PPIL1 0.99

SHMT2 0.99

SERF1B 0.98

OAF 0.98

LAS1L 0.98

SUPT16H 0.98

SLC39A3 0.98

NME4 0.98

SOD2 0.98

DDX1 0.98

EEF1B2 0.98

PRDX2 0.98

PRMT6 0.98

U2AF1 0.98

WDHD1 0.97

GGH 0.97

CENPA 0.97

RFC4 0.97

MAT2A 0.97

PELO 0.97

ACTL6A 0.97

STEAP3 0.97

RAN 0.97

ENOSF1 0.97

PCOLCE2 0.97

SCRIB 0.97

EIF3S2 0.97

PIGY 0.97

HIST1H2AJ 0.97

POLR3E 0.96

DPH5 0.96

HIST1H2BL 0.96

GTF3A 0.96

MYBBP1A 0.96

LMNB2 0.96

GOLPH4 0.96

CCT3 0.96

RPUSD4 0.96

SHQ1 0.96

QDPR 0.96

MRPL50 0.95

SLC39A3 0.95

MRPS30 0.95

OGFOD1 0.95

CDCA3 0.95

RG9MTD1 0.95

CCDC58 0.95

YIF1B 0.95

RPS7 0.95

ANAPC1 0.95

BMP7 0.95

ADSSL1 0.95

POLE 0.95

PUSL1 0.94

HCP1 0.94

ADSL 0.94

COX5A 0.94

ILF3 0.94

MRPL22 0.94

NFXL1 0.93

PCGF6 0.93

ASPM 0.93

SIP1 0.93

TAF15 0.93

THOC3 0.93

CKAP5 0.93

PSMD14 0.93

EIF4A1 0.93

RPL34 0.93

NDUFB7 0.93

NETO1 0.93

LARP1 0.93

MCM7 0.92

KDELC1 0.92

FEN1 0.92

CDCA5 0.92

CLNS1A 0.92

COPS3 0.92

DDX56 0.92

SAC3D1 0.92

SNRPB 0.92

BUB1B 0.92

ERICH1 0.92

ZBTB9 0.92

SNRPD2 0.92

HCAP-G 0.92

HSD17B8 0.92

ASNSD1 0.91

SF3B14 0.91

KIF23 0.91

ING2 0.91

TOP2A 0.91

TTK 0.91

SEH1L 0.91

PRDX3 0.91

KATNB1 0.91

POLE3 0.91

MTFMT 0.91

LSM7 0.91

TOP3A 0.91

STK6 0.91

SCAMP1 0.91

H2AFX 0.91

HNRPM 0.91

DLL3 0.91

BCCIP 0.9

MRPS11 0.9

ERCC2 0.9

SITPEC 0.9

SMS 0.9

CCDC41 0.9

HIST1H2AH 0.9

QTRT1 0.9

DUS1L 0.9

NUP155 0.9

DDX28 0.9

UCHL5 0.9

PYCR1 0.9

DDX55 0.9

TOMM20 0.9

LSM3 0.9

EEF1B2 0.9

LRP11 0.9

PRPS1L1 0.89

MYOHD1 0.89

PPIB 0.89

KLHL8 0.89

RPL3 0.89

DHCR7 0.89

HIST1H3H 0.89

MRPL28 0.89

ETFB 0.89

UBXD6 0.89

TCP1 0.89

VDAC2 0.89

HIST2H3C 0.88

AMICA1 0.88

RAVER2 0.88

FTSJ1 0.88

PXMP2 0.88

ACBD6 0.88

AKAP1 0.88

TST 0.88

CEP290 0.88

SMARCA3 0.88

DHODH 0.88

ADAM15 0.88

MRPL46 0.88

HEBP2 0.88

NDUFS3 0.88

ZNF259 0.88

HIST1H1B 0.88

ARL6IP2 0.88

SLC25A4 0.88

FAM44B 0.88

TFDP1 0.87

IARS 0.87

CCNB2 0.87

USP1 0.87

TRIM28 0.87

hfl-B5 0.87

FDFT1 0.87

PDXP 0.87

FAM98A 0.87

ZNF232 0.87

NUP37 0.87

RPL26L1 0.87

MRPS12 0.87

CBR1 0.87

MTHFD1 0.86

DTL 0.86

HSP90AA1 0.86

NPM1 0.86

CDCA1 0.86

WDR74 0.86

GCLM 0.86

ILVBL 0.86

LRRC59 0.86

SPBC25 0.86

ATP5J 0.86

LIAS 0.86

RPL10A 0.86

NOL11 0.86

SNRPC 0.86

ARMET 0.86

ATP5D 0.85

DUS3L 0.85

FANCB 0.85

WDR18 0.85

APRT 0.85

DDX19A 0.85

SIVA 0.85

NT5C3L 0.85

ZNF121 0.85

PPP1R14B 0.85

HARS2 0.85

ECHS1 0.85

IMP3 0.85

MMACHC 0.85

ZDHHC11 0.85

GTPBP3 0.84

HAGHL 0.84

MRPL15 0.84

THRA 0.84

EIF4B 0.84

PTGES3 0.84

B3GALT6 0.84

NEK2 0.84

WBSCR22 0.84

PPIF 0.84

SHMT1 0.84

HMGCS1 0.84

MRPL27 0.84

HDAC2 0.84

MULK 0.84

GAMT 0.84

PSMD3 0.84

TIMM9 0.84

SAAL1 0.84

SLC27A5 0.84

MCM6 0.83

BOLA2 0.83

ERH 0.83

PLK4 0.83

ANKRD16 0.83

EXO1 0.83

PYCR1 0.83

KIF20A 0.83

FANCA 0.83

LRRC34 0.83

TBCA 0.83

DGCR6L 0.83

ZWINT 0.83

ERO1L 0.83

NFKBIB 0.83

SLC29A1 0.83

STMN1 0.83

RPS3 0.83

DEPDC1 0.83

ELAC2 0.82

CCT6A 0.82

MT 0.82

PBEF1 0.82

DCXR 0.82

BTF3 0.82

CLEC4C 0.82

NKD2 0.82

PINX1 0.82

CLEC4C 0.82

AFG3L2 0.82

CDK2AP1 0.81

NOL7 0.81

DHX37 0.81

WDR5 0.81

HIST1H3A 0.81

PPIA 0.81

CSE1L 0.81

BLM 0.81

ELF3 0.81

VDAC3 0.81

LSM5 0.81

CSPG6 0.81

GNL2 0.81

MTP18 0.81

ZNF598 0.81

NOL1 0.81

PPIAL4 0.81

NDUFV1 0.81

HSPH1 0.81

RNF26 0.81

MELK 0.81

PUS1 0.81

SLC39A4 0.8

COQ9 0.8

SUCLG1 0.8

RPL27 0.8

ANKRD15 0.8

PPAN 0.8

YWHAE 0.8

TNFRSF8 0.8

UAP1 0.8

CARD9 0.8

PEX5 0.8

ANKRD27 0.8

NFKBIL2 0.8

PCNA 0.8

MRPL48 0.8

CLPP 0.8

TCEB2 0.8

CHAF1A 0.8

MPHOSPH1 0.79

FBXO5 0.79

CHCHD1 0.79

MTX1 0.79

WRNIP1 0.79

PLTP 0.79

PARK7 0.79

EXOSC8 0.79

CEBPB 0.79

IARS2 0.79

NSDHL 0.79

NOC4L 0.79

RDH13 0.79

PDHA1 0.79

ING5 0.79

TH1L 0.79

CHEK1 0.79

TXNRD1 0.78

RNH1 0.78

MRPL32 0.78

IRAK1 0.78

SLC29A2 0.78

POLDIP2 0.78

IPO11 0.78

CHCHD8 0.78

TRIT1 0.78

EME1 0.78

COX5B 0.78

SSBP1 0.78

RSAD1 0.78

HSPA14 0.78

NOLA1 0.77

HIST1H2BJ 0.77

RGMB 0.77

NARS 0.77

MRPL1 0.77

SLCO4A1 0.77

HNRPR 0.77

ZNF519 0.77

ILF2 0.77

MAP1B 0.77

BIVM 0.77

DPY19L2 0.77

SF3A3 0.77

BCAT2 0.77

ATP5A1 0.77

THOP1 0.77

OIP5 0.77

SAE1 0.77

ZNF664 0.77

QARS 0.77

CBS 0.77

RCN2 0.76

ATP5G3 0.76

YWHAH 0.76

FANCD2 0.76

ORC5L 0.76

RPP25 0.76

YARS2 0.76

PLCXD1 0.76

CCT8 0.76

RPL7L1 0.76

E2F8 0.76

CLUAP1 0.75

IDE 0.75

CRI2 0.75

CENPJ 0.75

NUSAP1 0.75

PDXK 0.75

RPL7 0.75

ATP5I 0.75

SARS2 0.75

PPP2R5D 0.75

ITGB3BP 0.75

WDR57 0.75

COX6A1 0.75

RPL7A 0.75

HNRPA0 0.75

HRAS 0.75

MAPKAPK5 0.75

MTERFD1 0.75

RPL6 0.75

PAWR 0.74

GYPC 0.74

E2F2 0.74

WBP5 0.74

MTIF2 0.74

RRAGD 0.74

ZWILCH 0.74

DSTN 0.74

HIST1H3B 0.74

HDLBP 0.74

HMGB2 0.74

MTHFS 0.74

ZNHIT2 0.74

AP2S1 0.74

ALPL 0.74

NDUFS1 0.74

NMD3 0.74

IDH2 0.74

ATP5H 0.74

SMTN 0.74

PHF5A 0.74

ASNS 0.74

MRPS21 0.73

PSMC3 0.73

TARS 0.73

RFC2 0.73

VBP1 0.73

AVEN 0.73

CDC2 0.73

TRIM65 0.73

LETM1 0.73

GADD45GIP1 0.73

2'-PDE 0.73

FKSG14 0.73

NDUFS8 0.73

RPS5 0.73

MANEAL 0.73

UBE2C 0.73

KLHL21 0.73

HNRPD 0.73

METAP2 0.73

CEP55 0.73

RPL9 0.73

UCRC 0.73

VPS13A 0.72

RPL36 0.72

SLC43A1 0.72

HSPBP1 0.72

SMC2L1 0.72

SNAPC4 0.72

TSC22D1 0.72

NOC2L 0.72

CHAF1B 0.72

UQCRFS1 0.72

TIMM10 0.72

AGPS 0.72

RANBP2 0.72

WDR67 0.72

VARSL 0.72

SCO1 0.72

CSTF3 0.72

CLPX 0.71

SLC1A5 0.71

MRPL55 0.71

RBM13 0.71

ARD1A 0.71

PIM3 0.71

DAP3 0.71

ZNF22 0.71

USP10 0.71

CAMKK2 0.71

HIST1H2AE 0.71

RABGGTB 0.71

MYH3 0.71

EIF5A 0.71

RPA3 0.71

PRR6 0.71

BRF1 0.71

CCDC8 0.71

EEF2 0.71

ACAT2 0.71

HIST1H4A 0.7

KNTC1 0.7

MYB 0.7

WRN 0.7

STC2 0.7

VPS26B 0.7

PRPF4 0.7

ATP5C1 0.7

MRPL38 0.7

IMMT 0.7

ABHD6 0.7

SEPHS1 0.7

PSPH 0.7

LCMT2 0.7

DONSON 0.7

UBE2G1 0.7

SYNCRIP 0.69

MDH1 0.69

AURKAIP1 0.69

PSMD1 0.69

ACTR3B 0.69

TTC4 0.69

MRPL51 0.69

FAM72A 0.69

PSMB6 0.69

SLC25A3 0.69

DEAF1 0.69

COX7A2 0.69

IDI2 0.69

TFDP2 0.69

ISOC1 0.69

NUDT5 0.69

PHF10 0.69

SMARCC1 0.69

ADSS 0.69

SERBP1 0.69

LIG1 0.69

EIF4G1 0.69

ACLY 0.69

PAFAH1B3 0.69

NUP107 0.69

RPLP0 0.69

LRPPRC 0.69

RPS14 0.69

ZBED4 0.69

SNRPA 0.69

SUMF2 0.69

TIGD5 0.68

EIF4EBP1 0.68

SKP2 0.68

PGAM4 0.68

GCSH 0.68

GRWD1 0.68

WDR54 0.68

HIST1H2BG 0.68

NUP85 0.68

DCI 0.68

MYH10 0.68

APIP 0.68

DYNLL2 0.68

UBE2S 0.68

NDUFC1 0.68

ZNF593 0.68

NANP 0.68

CDK5 0.68

KCTD3 0.68

POLR2L 0.68

FAHD1 0.68

RPUSD2 0.68

EIF3S6 0.68

POLG2 0.68

ELOVL6 0.68

POLR3B 0.68

AARS 0.67

PTCD1 0.67

NOB1P 0.67

HSPC152 0.67

PTMA 0.67

NDUFA3 0.67

F2R 0.67

PSMB2 0.67

NDUFA12 0.67

LSS 0.67

UBL4A 0.67

DNAJC7 0.67

DLD 0.67

AAMP 0.67

SEC61G 0.67

RASAL1 0.67

AP3M2 0.67

TUBB 0.67

TTC3 0.67

ZP3 0.67

NDUFB8 0.67

CIB1 0.67

ACP1 0.67

CEP152 0.67

THUMPD2 0.67

RPL37 0.66

TMPO 0.66

NUP205 0.66

DHX30 0.66

MRPL18 0.66

HIST1H2AM 0.66

MSH6 0.66

SKP2 0.66

PRPF8 0.66

NDUFS6 0.66

TYSND1 0.66

DFFB 0.66

TOE1 0.66

XRCC3 0.66

SLC19A1 0.66

ACSL3 0.66

TAF9 0.66

PSMB7 0.66

XPO4 0.66

TMEM93 0.66

NDUFA8 0.66

PREP 0.66

STRAP 0.66

PEO1 0.66

PTRH2 0.66

SHCBP1 0.66

MLSTD1 0.66

LGTN 0.66

ABCB10 0.66

ZNF9 0.66

SCAMP1 0.65

HINT2 0.65

TEX10 0.65

ACO2 0.65

CUTC 0.65

PLCG1 0.65

NAGPA 0.65

PFKP 0.65

MSTO1 0.65

PTPLAD1 0.65

hCAP-D3 0.65

HIST1H3E 0.65

15E1.2 0.65

ATP5H 0.65

PTD004 0.65

MRPS5 0.65

HS6ST1 0.65

PKN3 0.65

SMC4L1 0.65

DGCR6 0.65

DYRK3 0.65

METT10D 0.65

HES4 0.65

76P 0.64

LRRC47 0.64

RIOK1 0.64

RPL35A 0.64

DNAJC9 0.64

VRK1 0.64

PMS1 0.64

MRPL20 0.64

AURKB 0.64

SPATA5L1 0.64

RAB15 0.64

TBC1D4 0.64

RPL23A 0.64

Ells1 0.64

NXT1 0.64

UBXD5 0.64

PSARL 0.64

BCL2L12 0.64

HIST1H4I 0.64

IDH3B 0.64

DDX41 0.64

NDUFB9 0.64

LAMC3 0.64

PRR11 0.64

HNRPDL 0.64

NUDT6 0.64

AK2 0.63

NDUFB3 0.63

DHX29 0.63

H2BFS 0.63

G6PD 0.63

GHITM 0.63

USP39 0.63

NSUN2 0.63

TOMM34 0.63

EZH2 0.63

UNG 0.63

BANF1 0.63

RPS9 0.63

MRPS27 0.63

TBL3 0.63

SUV420H2 0.63

UROD 0.63

DRG1 0.63

ETF1 0.63

CDCA4 0.63

ATOX1 0.63

MRPL22 0.63

EIF3S7 0.63

BPNT1 0.63

DFFA 0.63

SNRPG 0.63

RNASEH2A 0.63

G3BP 0.63

DDX51 0.63

UTP15 0.63

FLAD1 0.63

METTL5 0.63

PTDSS2 0.63

PTGES2 0.63

NDUFA2 0.63

SET 0.63

IPO4 0.63

PAXIP1 0.63

ASF1B 0.63

NIP7 0.62

PSMB5 0.62

NARG1 0.62

CSNK1E 0.62

PET112L 0.62

HNRPM 0.62

KIF4A 0.62

TOMM22 0.62

UQCRH 0.62

POLQ 0.62

DPM3 0.62

RPL12 0.62

PLA2G4A 0.62

GLRX2 0.62

NUDT9 0.62

NIFUN 0.62

PRKRA 0.62

COX11 0.62

MRPL47 0.62

FREQ 0.62

ORC6L 0.62

RIMBP2 0.62

AP1S1 0.62

NELF 0.62

HIST1H3G 0.62

PPID 0.62

THOC1 0.61

APPBP1 0.61

HIST1H3I 0.61

SFXN4 0.61

HIST1H2AI 0.61

SSSCA1 0.61

ARL5 0.61

YARS 0.61

SGTA 0.61

HERC2 0.61

AMH 0.61

CDC25C 0.61

TAF6L 0.61

RNASEH1 0.61

DDB1 0.61

AK3L2 0.61

ATP5F1 0.61

HIST1H3F 0.61

SIAHBP1 0.61

DHFR 0.61

UBQLN4 0.61

CCDC66 0.6

XYLT2 0.6

TMEM29 0.6

NAP1L1 0.6

CCDC72 0.6

RNASEN 0.6

GCDH 0.6

TYMS 0.6

FUSIP1 0.6

LDHA 0.6

RPL15 0.6

MRPL11 0.6

TESC 0.6

PTDSS1 0.6

AQR 0.6

POLA2 0.6

SAS10 0.6

GLRX2 0.6

RPL5 0.6

CSTF2 0.6

PDHX 0.6

DC2 0.6

UGCGL1 0.6

UBE2V2 0.6

eIF2A 0.6

NOL9 0.6

ASPN 0.6

GNB2L1 0.6

BRMS1 0.6

COX7B 0.59

CBX3 0.59

GAB1 0.59

PABPC1 0.59

CNIH 0.59

DPP3 0.59

USP5 0.59

PSMA6 0.59

ACAA2 0.59

HIST1H4H 0.59

PRPF19 0.59

NUCB2 0.59

TROAP 0.59

COPS6 0.59

MRPL54 0.59

TXNDC14 0.59

PDCD2 0.59

PWP2H 0.59

XBP1 0.59

TOMM7 0.59

TMPO 0.59

GK 0.59

ACYP1 0.59

CENPH 0.59

HIST1H4C 0.59

ZNRD1 0.59

ZNF573 0.59

CCNB1IP1 0.59

COQ2 0.59

RPP21 0.58

TUSC4 0.58

HNRPC 0.58

TSTA3 0.58

MAD2L2 0.58

GNL3L 0.58

PDCL3 0.58

DHX9 0.58

RANBP3 0.58

SLD5 0.58

SBNO1 0.58

WDR19 0.58

PSMA3 0.58

TMEM4 0.58

STMN3 0.58

TMED3 0.58

SGOL2 0.58

ABCA2 0.58

KCNQ2 0.58

CENPB 0.58

COMTD1 0.58

EXDL2 0.58

KIF2C 0.58

COQ3 0.58

TREX1 0.58

GSS 0.58

TMEM14B 0.58

ZFAND1 0.57

SFRS15 0.57

PFKM 0.57

MRPS7 0.57

SMYD3 0.57

ASTN2 0.57

PPP2R5D 0.57

MRPL52 0.57

SGOL1 0.57

TCEB1 0.57

PKD1 0.57

RSRC1 0.57

HSP90B1 0.57

FIS1 0.57

MRPS21 0.57

MATR3 0.57

MYST1 0.57

TDG 0.57

RIF1 0.57

RPS8 0.57

LSM12 0.57

MTRR 0.57

MRPL35 0.57

RBM19 0.57

NY-SAR-48 0.57

PPIL5 0.57

RPN1 0.57

KPNA3 0.57

HIST1H2BE 0.57

WDR71 0.57

COMMD9 0.57

RPL26 0.57

PSTPIP2 0.56

CHAC2 0.56

MTA1 0.56

MRPL9 0.56

ILF3 0.56

COX4I1 0.56

LARP2 0.56

AGPAT7 0.56

SCO2 0.56

SERPINF1 0.56

SRP68 0.56

NOP5/NOP58 0.56

CBX6 0.56

ALDH3A2 0.56

FLAD1 0.56

DYM 0.56

WDR66 0.56

HIST1H2AB 0.56

MFNG 0.56

ANP32C 0.56

MRE11A 0.56

MRRF 0.56

NDUFA11 0.56

FRAT2 0.56

ZNF30 0.56

DDT 0.56

DNAJA2 0.56

PRIM1 0.56

MRPS35 0.56

HTATSF1 0.56

HABP4 0.56

NOMO3 0.56

ADCK2 0.56

LEO1 0.56

SKIV2L2 0.56

UQCRC2 0.56

PHOSPHO2 0.56

CTSC 0.55

HARS 0.55

ZC3HC1 0.55

NDUFA1 0.55

CCDC25 0.55

MTX2 0.55

ARS2 0.55

CINP 0.55

SLC25A5 0.55

WBSCR18 0.55

HIST2H2AB 0.55

PSMA7 0.55

PSMA2 0.55

HOMER1 0.55

DDX24 0.55

YWHAQ 0.55

MRPL41 0.55

SF3B2 0.55

CRSP8 0.55

OBFC2B 0.55

WEE1 0.55

USP14 0.55

DRG2 0.55

PSMC4 0.55

PRCC 0.55

NPIP 0.55

UBTF 0.55

SUCLA2 0.55

XPO6 0.55

DONSON 0.55

DDX54 0.55

ATP2A2 0.54

SRP46 0.54

ATP5O 0.54

CYCS 0.54

MBD3 0.54

TPRKB 0.54

MRPS12 0.54

WDR61 0.54

SDAD1 0.54

TXNL5 0.54

ABHD11 0.54

CHCHD3 0.54

CDC5L 0.54

GUF1 0.54

PRPSAP1 0.54

AFF3 0.54

SFRS9 0.54

VPS13A 0.53

NDUFA9 0.53

CSTF3 0.53

GTF2H3 0.53

ZNF696 0.53

RCN1 0.53

PACAP 0.53

FAM51A1 0.53

PGAP1 0.53

KDELR2 0.53

PGAM1 0.53

SSR3 0.53

AMMECR1 0.53

CDC23 0.53

CHERP 0.53

NANS 0.53

CAMSAP1 0.53

IPO9 0.53

LRFN4 0.53

NHP2L1 0.53

SF3B5 0.53

SLC25A28 0.53

TIMELESS 0.53

TUBA3 0.53

HP1BP3 0.53

ERAL1 0.53

GPSN2 0.53

SACS 0.53

CNAP1 0.52

GPS1 0.52

EIF3S6IP 0.52

CDR2 0.52

B3GALNT2 0.52

SNX27 0.52

MRPL44 0.52

ADRM1 0.52

TCF19 0.52

SLC39A14 0.52

EIF3S12 0.52

ERGIC3 0.52

ME1 0.52

CSRP2 0.52

TINP1 0.52

SLC9A3R1 0.52

PSMA4 0.52

SOCS4 0.52

TMEM119 0.52

POLD3 0.52

TAF5 0.52

ZNF714 0.52

ALG1 0.52

CMTM8 0.52

HNRPA2B1 0.52

HPS4 0.52

POLR2D 0.52

GTPBP3 0.52

ACTR6 0.52

RAB40B 0.52

STARD7 0.52

SBDS 0.52

FBXO4 0.52

TPI1 0.51

SEC23A 0.51

TBL2 0.51

ZNF354B 0.51

NDUFA6 0.51

WDR36 0.51

PGM1 0.51

RNPS1 0.51

POR 0.51

TNPO1 0.51

MRPL35 0.51

HSD11B2 0.51

AOF2 0.51

RNUXA 0.51

CPSF4 0.51

UBE2T 0.51

UBA2 0.51

PNMA1 0.51

MRFAP1 0.51

PNMA2 0.51

XRCC1 0.51

ENY2 0.51

MORF4L2 0.51

SCARB1 0.51

RFXAP 0.51

EXOSC4 0.51

DNAJB6 0.51

DHRS4 0.51

TRAPPC3 0.51

REXO4 0.5

CDK5RAP1 0.5

NUP93 0.5

NUBP2 0.5

ZNF37A 0.5

CYP51A1 0.5

SLC25A6 0.5

UBADC1 0.5

BRD8 0.5

GRHPR 0.5

ZNF607 0.5

ESD 0.5

SLK 0.5

TNFRSF10A 0.5

RAD51C 0.5

ZAK 0.5

WBSCR1 0.5

TTLL4 0.5

HADHA 0.5

LARS2 0.5

THRAP3 0.5

NSUN4 0.5

SNFT 0.5

HSDL1 0.5

UBE2O 0.5

ENPP2 0.5

ATP8B2 0.5

NDUFA4 0.5

RBMX 0.5

TBC1D14 0.5

SLC35A4 0.5

TRAPPC4 0.5
