# Supplementary material for: Time-Dependent c-Myc Transactomes Mapped by Array-Based Nuclear Run-On Reveal Transcriptional Modules in Human B Cells
Source: PLoS One. 2010 Mar 15;5(3):e9691. doi: 10.1371/journal.pone.0009691 (PMC2837740; doi:10.1371/journal.pone.0009691)
Supplement: Table S3 — (0.17 MB DOC) [file pone.0009691.s004.doc]

**TABLE S3. Nuclear Run-on Down-regulated Genes – Time Course**

**15 minutes**

Symbol NRO 15'

KLF10 -0.82

HIST2H2BF -0.68

HIST1H2AI -0.66

CLK1 -0.57

TXNIP -0.56

HIST1H3J -0.53

COL5A3 -0.52

PARP10 -0.52

ZFP36L1 -0.51

**30 minutes**

Symbol NRO 30'

MAP3K8 -0.97

ZNF91 -0.83

HIST1H4L -0.74

HIST1H2AK -0.74

ATPIF1 -0.72

UGDH -0.72

SLC25A14 -0.71

CCNB1 -0.67

CNIH4 -0.66

ST8SIA4 -0.63

PLEKHF2 -0.63

NUDT4 -0.63

HIST1H4I -0.62

PROSC -0.58

SERBP1 -0.58

TIFA -0.58

SLC35B3 -0.58

HIST1H4A -0.58

CCR2 -0.57

PSMA2 -0.56

MKKS -0.55

PYGO2 -0.55

PSME1 -0.55

ARPC5 -0.55

TMEM70 -0.54

HIST1H4K -0.54

PCF11 -0.53

DRB1 -0.53

HIST1H1B -0.52

EVI2A -0.52

KLHDC5 -0.52

HIST1H1C -0.51

SLC39A1 -0.51

EVI2A -0.5

CRI1 -0.5

**1 hour**

Symbol NRO 1hr

HIST1H2BD -2.06

ZNF658 -1.98

CUL3 -1.84

H3F3B -1.69

AHCTF1 -1.67

IFIT2 -1.65

EIF4A2 -1.65

HIST1H2AM -1.56

HIST1H2AJ -1.53

HIST1H3G -1.46

RAP1B -1.44

HIST2H2BE -1.43

HIST1H2AD -1.43

RFXDC2 -1.43

SEL1L -1.42

UBE2J1 -1.38

SLC38A2 -1.37

PTPRO -1.36

RPS6KB1 -1.35

PRPF38B -1.35

ZNF507 -1.35

NIN -1.34

HIST1H2BG -1.34

KLF2 -1.31

HIST1H2AG -1.3

TXNL5 -1.3

ARL6IP5 -1.3

HIST1H1D -1.3

AGPAT5 -1.27

HNRPF -1.26

HIST1H2AH -1.26

ZNF658B -1.24

HBLD1 -1.23

SART2 -1.22

COMMD3 -1.21

ENPP4 -1.21

CCDC6 -1.21

ITGB1BP1 -1.21

NFKBIZ -1.2

YOD1 -1.17

MGEA5 -1.17

SMNDC1 -1.16

SACM1L -1.15

HTLF -1.15

KRT10 -1.15

CFL2 -1.15

DENR -1.14

IFNA2 -1.14

HIST1H2BF -1.14

KCTD9 -1.14

EIF3S6 -1.14

WASPIP -1.14

HIST1H3I -1.13

HIST1H2BD -1.12

FBXO5 -1.12

GRSF1 -1.11

YPEL2 -1.11

RICTOR -1.1

SFRS3 -1.1

DNAJC15 -1.09

05-Mar -1.09

IL4R -1.09

SELT -1.08

JOSD3 -1.08

SS18L2 -1.08

RSBN1 -1.08

BHLHB3 -1.07

RAB14 -1.06

HIST1H2AB -1.06

ACTG1 -1.06

IFIT1 -1.05

TLOC1 -1.05

ATP5G3 -1.04

ZNF273 -1.04

HADHSC -1.04

HIST1H3H -1.03

CBFB -1.03

LEMD3 -1.02

HMGCR -1.02

SFRS10 -1.02

HIST1H2BI -1.01

HIST1H2AC -1

C1D -1

ZNF613 -1

UBLCP1 -1

SLTM -1

RPSA -1

SMARCE1 -1

RTF1 -0.99

HSBP1 -0.99

RAB33B -0.99

VAPA -0.99

GCNT1 -0.99

FXR1 -0.99

SPRED1 -0.98

USP47 -0.98

VCPIP1 -0.98

SAP18 -0.97

GPIAP1 -0.97

OSBPL8 -0.96

PSMD9 -0.96

ZNF679 -0.96

REEP5 -0.95

IFNGR1 -0.95

PLAA -0.95

RNGTT -0.95

RAD23B -0.94

CLK4 -0.94

FAM49A -0.94

SUZ12 -0.94

HIST1H4E -0.94

HIST1H3F -0.93

TIMM17A -0.92

SLC2A1 -0.92

HDDC2 -0.91

JMJD1C -0.91

CCT5 -0.9

YWHAB -0.9

SNX10 -0.9

SC4MOL -0.9

CCDC23 -0.9

FAM102B -0.9

ZNF287 -0.9

SEC10L1 -0.9

EIF2S2 -0.9

CHD1 -0.89

HIST1H2AE -0.89

ZNF222 -0.89

CASC1 -0.89

ANKFY1 -0.89

MTFR1 -0.89

LCMT2 -0.89

IFIT5 -0.89

PPT1 -0.89

KDELR3 -0.88

FAM29A -0.88

CUL5 -0.88

AGA -0.87

DCP2 -0.87

TMEM77 -0.86

METTL7A -0.86

LSM8 -0.86

RALBP1 -0.85

ZCCHC6 -0.85

SUB1 -0.85

PPCS -0.85

PHTF2 -0.85

HIST1H2BH -0.84

GPR15 -0.84

ZMPSTE24 -0.84

TBC1D9 -0.84

FCRLM2 -0.84

CCDC65 -0.84

DDX26 -0.83

KLHL2 -0.83

DNAJA5 -0.83

ZNF189 -0.82

LRRC37B -0.82

STIL -0.82

SNAP23 -0.82

AFTIPHILIN -0.82

PNRC2 -0.81

RAP1A -0.81

PDCD10 -0.81

SNX2 -0.81

ZNF420 -0.81

IFIT3 -0.8

RANBP6 -0.8

bA16L21.2.1 -0.8

HIST1H2BE -0.8

ZFPM1 -0.79

PTGES3 -0.79

CD69 -0.79

RAB10 -0.79

TXNDC9 -0.79

ZNF532 -0.79

RPA3 -0.79

ZBTB33 -0.78

TRIT1 -0.78

HIST2H3C -0.78

PIK3R1 -0.78

UBE2E1 -0.78

DYRK1A -0.78

TRIM2 -0.78

ATP5E -0.78

MTMR9 -0.78

ZCCHC7 -0.78

DCAL1 -0.77

AZIN1 -0.76

CCND3 -0.76

DEK -0.76

DAAM1 -0.76

RPLP1 -0.76

SLC25A17 -0.76

BIRC3 -0.76

HIST1H2BM -0.76

TFRC -0.76

RPS19 -0.75

TLR1 -0.75

TFAM -0.75

RG9MTD1 -0.75

TEX2 -0.75

AUH -0.75

DAPP1 -0.75

CDC40 -0.75

HSPH1 -0.75

BRWD1 -0.75

HIST1H2BL -0.75

DNAJB9 -0.75

PECI -0.74

FBXW7 -0.74

ABCA10 -0.74

WASL -0.74

SOCS4 -0.74

TPD52 -0.74

CEBPG -0.74

PGBD4 -0.74

RNPC2 -0.74

PRMT6 -0.74

07-Mar -0.74

MRPL50 -0.74

HIST1H1E -0.74

ORC4L -0.73

IFIT3 -0.73

H2AFZ -0.73

PDIK1L -0.73

SESN1 -0.73

ZNF721 -0.73

GLUL -0.73

P18SRP -0.73

FANCF -0.73

NACA -0.73

RIOK3 -0.73

CRSP9 -0.73

ANKRD12 -0.73

HIST1H3C -0.73

H2AFJ -0.72

AEBP2 -0.72

PPAPDC2 -0.72

CCT4 -0.72

BET1 -0.71

PRO0149 -0.71

NIPBL -0.71

HIST1H4D -0.71

HBS1L -0.71

TOMM7 -0.7

GEMIN7 -0.7

GMFB -0.7

ANXA4 -0.7

DNTTIP2 -0.7

GLE1L -0.7

ZBTB41 -0.7

HIST2H2AA -0.7

AZI2 -0.7

HIST2H2AB -0.7

TBPL1 -0.7

ZNF197 -0.69

PIGY -0.69

STRAP -0.69

SGPP1 -0.69

OAS2 -0.69

ATP6V1A -0.69

ID2 -0.68

IGJ -0.68

BMP2K -0.68

RPL30 -0.68

ZNF607 -0.68

TM2D3 -0.68

KAZALD1 -0.68

RASGRP1 -0.68

RAB18 -0.68

GRINL1A -0.67

KPNA2 -0.67

ZNF100 -0.67

PFDN4 -0.67

ADD1 -0.67

KPNA3 -0.67

TEAD2 -0.67

PTP4A1 -0.67

SIRT1 -0.67

PIK3AP1 -0.67

CCNT2 -0.67

ANP32B -0.67

PAFAH1B1 -0.66

CALM2 -0.66

SP4 -0.66

ACBD3 -0.66

CCNG2 -0.66

SERTAD3 -0.66

PPP1R3D -0.66

CD47 -0.66

LINS1 -0.66

KMO -0.66

MALT1 -0.66

HIST2H2AC -0.66

SMC6L1 -0.66

METTL5 -0.66

SFRS7 -0.66

TLR4 -0.65

APRIN -0.65

WDR40A -0.65

TMSL3 -0.65

RWDD2 -0.64

TBCA -0.64

HIST1H3D -0.64

GBP3 -0.64

LRMP -0.64

PDCD4 -0.64

DNAJC19 -0.64

CBR4 -0.64

B3GNT1 -0.64

ZNF271 -0.64

MEF2C -0.64

RNF113A -0.64

LSM6 -0.64

DERL1 -0.64

HS2ST1 -0.64

BRD2 -0.64

ZNF570 -0.63

GHITM -0.63

PURA -0.63

TGFBR1 -0.63

MRCL3 -0.63

XPO1 -0.63

COMMD8 -0.63

ZCRB1 -0.63

ABCB10 -0.63

CDC23 -0.63

H2AFJ -0.63

TRIM59 -0.62

ZNF708 -0.62

ZNF596 -0.62

HIST1H2BC -0.62

CUGBP2 -0.62

CRSP2 -0.62

ITR -0.62

SMAP -0.62

ALS2 -0.62

MTERFD3 -0.62

ZNF85 -0.61

ZCSL2 -0.61

WBP4 -0.61

PYHIN1 -0.61

MBD4 -0.61

TRAF6 -0.61

RPS28 -0.61

HSPA5 -0.61

BRF2 -0.6

LY6E -0.6

PBK -0.6

ATP6V1G1 -0.6

GLT8D2 -0.6

TIMM10 -0.6

ABCA1 -0.6

CYB5-M -0.6

ZNF490 -0.6

RANBP2 -0.6

RBM22 -0.6

STARD7 -0.6

NPM1 -0.6

CLTC -0.59

SLC11A2 -0.59

MSL3L1 -0.59

EAF2 -0.59

NUDCD1 -0.59

RRAGB -0.59

GSTO1 -0.59

CCNG1 -0.59

MBTD1 -0.59

CETN2 -0.59

ALG5 -0.59

TRAF3IP3 -0.59

BCL2L1 -0.59

HSPA4 -0.59

G1P2 -0.59

BPGM -0.59

DPY19L4 -0.59

UBE1DC1 -0.59

IER3IP1 -0.59

MRPL49 -0.59

THUMPD2 -0.58

NT5C3 -0.58

ZNF383 -0.58

ID3 -0.58

AASDH -0.58

ATF4 -0.58

FBXL3 -0.58

MUT -0.58

MCOLN2 -0.58

PCGF4 -0.58

ZA20D2 -0.58

TMEM9B -0.58

ZNF555 -0.58

PPP1R8 -0.58

ZNF404 -0.58

WEE1 -0.58

UBE2B -0.58

JAZF1 -0.58

PPP1CC -0.58

YTHDC1 -0.58

CNOT1 -0.58

DLAT -0.57

PCAF -0.57

ARHGAP5 -0.57

WDR33 -0.57

BRWD1 -0.57

CNTF -0.57

PSMC1 -0.57

GALNT1 -0.57

CDCA7 -0.57

VBP1 -0.57

RPL6 -0.57

RPL37A -0.57

PPP1CB -0.57

MDM2 -0.57

PMAIP1 -0.57

UBE2D3 -0.57

TXNDC4 -0.57

PAPOLA -0.57

YIPF5 -0.57

OAS1 -0.57

ZNF181 -0.57

FNBP1 -0.57

ZNF580 -0.57

CHCHD2 -0.56

PSMD7 -0.56

ZNF547 -0.56

WDR75 -0.56

CCNL1 -0.56

ATG5 -0.56

HNRPDL -0.56

NAP1L1 -0.56

FBXO28 -0.56

PIGA -0.56

CITED2 -0.56

PPP1R2 -0.56

UBE2D2 -0.56

EHD1 -0.56

FXN -0.56

MXD4 -0.56

WWP1 -0.56

ITCH -0.55

ASTE1 -0.55

IRF1 -0.55

SWAP70 -0.55

CRNKL1 -0.55

NDUFAB1 -0.55

AIF1 -0.55

PLCL2 -0.55

CSTF2T -0.55

SUHW4 -0.55

RNF6 -0.55

LSM14A -0.55

RPL15 -0.55

PAQR8 -0.55

NIP30 -0.55

CEBPD -0.55

AKAP11 -0.55

MRPL35 -0.55

CAPZA2 -0.55

ZNF670 -0.55

TMEM87A -0.54

FBXW7 -0.54

BBS7 -0.54

HCP5 -0.54

VPS54 -0.54

ASXL2 -0.54

EXOC8 -0.54

FPGT -0.54

GLRX -0.54

MLR2 -0.54

TSN -0.54

ITGAE -0.54

ZNF313 -0.54

CAMK2D -0.54

FAM48A -0.53

UQCRH -0.53

ZNF546 -0.53

PCMTD1 -0.53

NARG2 -0.53

CARF -0.53

FAM49B -0.53

FH -0.53

SLC35A5 -0.53

TXNDC -0.53

ITGB1BP1 -0.53

SNAG1 -0.53

PXMP3 -0.53

ASF1A -0.53

VDP -0.53

RAB31 -0.53

HIST1H3A -0.53

MS4A7 -0.53

ZNF435 -0.52

ANKRD13C -0.52

RNF111 -0.52

HINT2 -0.52

FAM82B -0.52

SRRM1 -0.52

TRAPPC2 -0.52

ZNF17 -0.52

SPFH2 -0.52

RSBN1L -0.52

KLHL14 -0.52

TP53TG3 -0.52

RNF141 -0.52

ARMCX5 -0.52

KIF20A -0.52

PREI3 -0.52

PTK9 -0.52

TUBA6 -0.51

ZNF92 -0.51

ARHGAP25 -0.51

GPR160 -0.51

IFRD1 -0.51

RPS29 -0.51

EED -0.51

OSRF -0.51

ZNF709 -0.51

OXR1 -0.51

SLC25A28 -0.51

FAM44B -0.51

SART1 -0.51

WDR22 -0.5

MRPL44 -0.5

MIER1 -0.5

IPLA2(γ) -0.5

CD1D -0.5

RWDD4A -0.5

UBL3 -0.5

NDUFA4 -0.5

MRPS30 -0.5

TAS2R50 -0.5

ORC5L -0.5

MTRR -0.5

REV1L -0.5

**3 hour**

Symbol NRO 3hr

PHF21A -1.22

TNF -1.22

MX1 -1.19

DMXL1 -1.16

ABCG1 -1.11

CD2AP -1.07

IRF7 -1.03

STAT1 -1.01

BMP2K -0.99

GLCCI1 -0.98

CPEB4 -0.98

FTH1 -0.97

LMO4 -0.97

ANTXR2 -0.97

RALGPS2 -0.94

RSAD2 -0.94

ZNF318 -0.93

NCOA3 -0.91

IFI16 -0.91

TACC1 -0.91

AIM2 -0.91

WDR33 -0.89

STAT1 -0.89

MTSS1 -0.88

OAS2 -0.87

MMD -0.86

RCHY1 -0.85

LONRF1 -0.84

KLHL13 -0.84

CDC42EP3 -0.84

HDAC9 -0.83

EPSTI1 -0.83

FYB -0.82

ADD3 -0.82

CD55 -0.82

DDIT4 -0.81

CROP -0.8

PTEN -0.8

OSBPL10 -0.79

CAMK2D -0.78

JAK2 -0.78

ATAD1 -0.78

ALS2CR2 -0.78

CPEB3 -0.78

FAM43A -0.77

FUT8 -0.76

ZBP1 -0.76

PYHIN1 -0.76

NFIL3 -0.76

TMEM23 -0.75

DUSP5 -0.75

ZNF403 -0.75

FUT8 -0.75

CAPN2 -0.74

SLC38A1 -0.73

UGCG -0.73

GLS -0.73

EIF1 -0.73

ADAR -0.73

PSMB10 -0.72

NTNG2 -0.72

CD79B -0.72

SAMD9L -0.72

SLC16A6 -0.72

SUCLG2 -0.72

KLF9 -0.72

RALGPS2 -0.71

ST3GAL5 -0.71

CPNE8 -0.71

GPX1 -0.7

ITGAM -0.7

MRPL40 -0.7

VPS37C -0.69

ZNF141 -0.69

WSB1 -0.69

SLC44A2 -0.69

DDX3X -0.69

ITM2B -0.69

TPI1 -0.68

UNC93B1 -0.68

PSME4 -0.68

ITGA4 -0.68

RCOR3 -0.68

SCARB2 -0.68

SCML4 -0.68

YPEL5 -0.67

MTM1 -0.67

FAM76B -0.67

SCYL1BP1 -0.67

PACSIN2 -0.67

ROD1 -0.67

TNFSF9 -0.66

ANXA2 -0.66

TMEM62 -0.66

PPP1R15A -0.66

IFI44L -0.66

EBI2 -0.65

NKTR -0.65

STRN3 -0.64

ZNF682 -0.64

RBAK -0.64

SULF2 -0.63

ZNHIT3 -0.63

VCL -0.63

ARID5B -0.63

CDC14A -0.63

PKD2L2 -0.63

BAZ2B -0.63

SNX6 -0.63

LIMD2 -0.63

TOE1 -0.62

POLG -0.62

RAE1 -0.62

HNRPH3 -0.62

TDRD7 -0.62

DFNA5 -0.62

ZNF238 -0.62

PCMT1 -0.62

MANBA -0.62

SNRPG -0.62

ARRDC4 -0.61

TMEM71 -0.61

MYLIP -0.61

PACAP -0.61

BIRC4BP -0.6

MBNL2 -0.6

TANK -0.6

PPM1M -0.6

OAS1 -0.6

PIK3CA -0.6

SOCS1 -0.6

CERKL -0.6

EIF2AK2 -0.6

LYZ -0.59

HLA-DMB -0.59

IFI44 -0.59

PTPN22 -0.59

SYF2 -0.59

SLC30A1 -0.59

C1GALT1 -0.59

CHIA -0.58

HLA-DPB1 -0.58

AMZ2 -0.58

SPON1 -0.58

RPL23A -0.58

ZFP91 -0.58

FBXO33 -0.58

USP6 -0.58

ELF1 -0.58

TNFSF10 -0.57

ZNF347 -0.57

VPS4B -0.57

ATP11A -0.57

GNB2L1 -0.57

INPPL1 -0.57

PRKD2 -0.57

ZFHX1B -0.57

NOL4 -0.57

PRG4 -0.57

MSH6 -0.57

RBM15 -0.57

MID1IP1 -0.57

LRRC16 -0.57

FGFRL1 -0.57

NCK2 -0.57

UBXD8 -0.56

CRYZL1 -0.56

PPM1B -0.56

PAQR3 -0.56

PPP3CC -0.56

TAP1 -0.56

HLA-DOB -0.56

SYPL1 -0.56

RNF19 -0.55

TNFRSF21 -0.55

ZFR -0.55

ATP5O -0.55

TLK1 -0.55

PTPRB -0.55

STARD3NL -0.55

MTMR6 -0.55

CTDSP1 -0.55

SNX3 -0.55

PEX3 -0.55

CD200R1 -0.55

BAZ2A -0.54

B3GALT4 -0.54

ZFP36 -0.54

MYNN -0.54

IFIH1 -0.54

LAIR2 -0.54

OSBPL11 -0.54

HMGB1 -0.54

MAD2L1BP -0.54

ABI1 -0.54

PAG1 -0.54

PFKFB4 -0.54

TUBB6 -0.54

ZNF539 -0.54

MNDA -0.53

C1RL -0.53

ALG2 -0.53

TLK2 -0.53

WDR33 -0.53

PAQR4 -0.53

NUP160 -0.53

RBMXL1 -0.53

PKN2 -0.53

BAG5 -0.53

ZNF20 -0.53

NFKBIE -0.52

TM2D3 -0.52

SNAPAP -0.52

GALNT12 -0.52

SLA/LP -0.52

ACSL5 -0.52

ZNF75A -0.52

RABEP1 -0.52

IGF2BP3 -0.52

RPS8 -0.52

DERA -0.52

SP100 -0.52

K-ALPHA-1 -0.52

SFRS11 -0.52

ARHGEF3 -0.52

THAP9 -0.52

RPS3A -0.52

ZNF644 -0.51

IFITM1 -0.51

ACTR6 -0.51

RAPGEF3 -0.51

DYNC1LI1 -0.51

ZNF295 -0.51

ZNF6 -0.51

ERGIC2 -0.51

RFP2 -0.51

ZNF680 -0.51

FAM8A1 -0.51

CDKN2D -0.51

CD68 -0.51

HES1 -0.51

ANAPC13 -0.51

PRKACB -0.51

FCHSD2 -0.51

OAS3 -0.51

UBE1L2 -0.51

ZNF588 -0.51

ALDH9A1 -0.51

LAP3 -0.51

TNFRSF14 -0.51

ZNF608 -0.51

ICK -0.51

SERTAD3 -0.5

PRIM2A -0.5

NLK -0.5

RNPC2 -0.5

ARHGEF7 -0.5

DAZAP2 -0.5

LAMC1 -0.5

YAF2 -0.5

POLR2D -0.5

KRIT1 -0.5

CDC14B -0.5

SPIB -0.5

P4HA1 -0.5

IL4R -0.5

GDI2 -0.5

**6 hour**

Symbol NRO 6hr

PRIC285 -1.13

SCARA5 -0.96

ABCD1 -0.83

MYO1G -0.83

PARP15 -0.8

PQLC1 -0.74

PFTK1 -0.73

AP1S2 -0.7

FGR -0.69

EBF -0.68

FGD3 -0.68

CD37 -0.68

OR13A1 -0.68

OAS2 -0.66

ARHGEF18 -0.66

RGS19 -0.65

HK1 -0.65

FER1L3 -0.65

SLC2A6 -0.65

ELOVL5 -0.65

G1P3 -0.64

HERC6 -0.63

PPP4R1 -0.62

CHSY1 -0.61

TMEPAI -0.61

SPAST -0.61

EMP3 -0.6

PALM -0.6

PARP9 -0.6

BST2 -0.6

VRK2 -0.6

SYBL1 -0.59

CD1C -0.59

LILRB4 -0.58

UCP2 -0.58

E2F2 -0.57

IQSEC1 -0.57

GNG2 -0.57

ZNF385 -0.57

ARHGAP9 -0.56

IFI35 -0.56

TBC1D10C -0.56

APAF1 -0.56

RNF38 -0.55

ZNF161 -0.55

ARHGAP27 -0.55

PTPRCAP -0.55

MOBKL2B -0.54

RGL2 -0.54

ISG20 -0.54

LYL1 -0.54

ST3GAL2 -0.54

CHKB -0.54

SAT -0.53

RNF31 -0.53

RBMS3 -0.53

PSAP -0.53

APOBEC3F -0.53

PARP12 -0.52

GNAI2 -0.52

FBXO34 -0.52

CD81 -0.52

RAI17 -0.52

GPR18 -0.52

MRPS6 -0.51

DOCK11 -0.51

RING1 -0.51

FBXO11 -0.51

AOF1 -0.5

EPB41L5 -0.5

OAS1 -0.5

TMCO3 -0.5

TLR7 -0.5

BNIP3L -0.5

CEACAM1 -0.5

PTTG1IP -0.5

PI4KII -0.5

PELI1 -0.5

**48 hours**

Symbol NRO 48hr

IL4I1 -2.4

01-Mar -2.26

UPB1 -2.2

SLAMF1 -1.98

CCL17 -1.95

LMO2 -1.87

PSG11 -1.87

PSG9 -1.86

ACE -1.84

EBI3 -1.84

CEACAM1 -1.82

TIMP1 -1.81

CUEDC1 -1.74

CCR3 -1.74

CREB5 -1.67

SSPN -1.66

BASP1 -1.61

MGAT4A -1.57

FMNL3 -1.52

CD300C -1.52

HLA-E -1.48

RCBTB2 -1.48

IL13RA1 -1.48

UBE1L -1.47

GRIN1 -1.47

COL9A2 -1.47

FSTL3 -1.46

RASGRP3 -1.45

TMEM37 -1.45

CCR7 -1.45

ITGB2 -1.45

CD72 -1.43

FSCN1 -1.42

LYST -1.42

SYT17 -1.42

PSD3 -1.42

FCRL4 -1.41

FAIM3 -1.39

TRAF1 -1.38

CD82 -1.37

INPP1 -1.37

NCF2 -1.37

LIPC -1.37

GSTM2 -1.36

CD58 -1.36

CD74 -1.34

MMP11 -1.33

LRCH1 -1.33

TMEM2 -1.33

HLA-DOA -1.32

FCRL1 -1.31

PFC -1.3

MVP -1.3

HLA-DQB1 -1.3

FCGR2A -1.29

GBP1 -1.29

HLA-DRB3 -1.28

FCRL3 -1.28

CNR2 -1.28

SATB1 -1.28

NCF1 -1.26

HLA-DRB5 -1.26

LGALS3BP -1.26

DGKA -1.26

H-plk -1.25

DOCK10 -1.25

HCST -1.25

BIN2 -1.25

WDFY2 -1.24

VPREB3 -1.24

HAVCR2 -1.23

PTGS1 -1.23

APCDD1 -1.22

ST3GAL1 -1.22

LTB -1.21

CYLN2 -1.21

ENTPD1 -1.21

GPR114 -1.21

GLIPR1 -1.2

HLA-DRB4 -1.19

CASP1 -1.19

HAK -1.19

APOB48R -1.19

LCP2 -1.18

PLEK -1.18

LHX2 -1.18

TGM5 -1.18

ST3GAL1 -1.18

PDE1B -1.17

GALNT10 -1.17

P2RY5 -1.17

GPR137B -1.17

CD40 -1.16

FCRL5 -1.16

LKAP -1.16

SLC2A3 -1.16

CCDC50 -1.16

CIITA -1.16

LY96 -1.15

SKIL -1.15

PCDHGA7 -1.15

MS4A1 -1.15

TM6SF1 -1.15

PARVG -1.14

DGKA -1.14

ACTA2 -1.14

IFI27 -1.14

NFKBIZ -1.13

ERO1LB -1.13

SERPINI1 -1.13

TTLL3 -1.13

SLAMF6 -1.13

UTRN -1.12

ABCA7 -1.11

HCK -1.11

CERK -1.11

MOXD1 -1.11

EVI2B -1.1

NICN1 -1.1

CD84 -1.1

HLA-DRB1 -1.1

PYCARD -1.1

MYO1F -1.09

VCL -1.09

YPEL3 -1.09

FCER1G -1.09

UBE2A -1.09

SLC2A14 -1.09

BLK -1.08

STK17B -1.08

ADAM8 -1.08

CD180 -1.08

SUSD1 -1.08

RNASE6 -1.08

ALOX5 -1.08

HSPA6 -1.07

CALCOCO2 -1.07

TAPBP -1.07

ARHGEF6 -1.07

FCRL3 -1.06

CD3G -1.06

CD44 -1.06

JUP -1.06

PTPN6 -1.06

DHRSX -1.06

EGR1 -1.06

ZDHHC21 -1.05

GNG3 -1.05

ALOX5AP -1.05

PTPRC -1.05

PDE4B -1.04

DPEP2 -1.04

CAPN12 -1.04

NPAL2 -1.04

XYLT1 -1.04

HLA-DRA -1.04

TRIM22 -1.04

PCDHGA6 -1.03

MOXD1 -1.03

TNFAIP2 -1.03

FGFR1 -1.03

CASP4 -1.03

CD53 -1.02

IL16 -1.02

SEMA4D -1.02

LMBRD1 -1.02

PRKCB1 -1.02

JAK3 -1.01

SEMA7A -1.01

IFITM2 -1.01

PHF1 -1.01

HLA-DMA -1.01

LILRA2 -1

TRIM34 -1

CTTNBP2NL -1

RRAS -1

PRKCB1 -1

CYBB -1

POLD4 -1

RAFTLIN -0.99

HRK -0.99

AKNA -0.99

GSN -0.99

SYTL1 -0.99

KLHL22 -0.99

LAPTM5 -0.99

MR1 -0.98

ANKRD44 -0.98

TEP1 -0.98

FCER2 -0.98

AK1 -0.98

CD24 -0.98

TMC6 -0.97

SLA -0.97

LGALS1 -0.97

RP3-473B4.1 -0.97

LBH -0.97

HLA-A -0.96

NT5E -0.96

TRIM5 -0.96

TAP2 -0.96

HLA-DPA1 -0.96

PRG1 -0.96

ADAMTSL5 -0.96

CTNND1 -0.96

ZNF549 -0.96

SLITL2 -0.96

PTPRC -0.96

IL10RB -0.95

CAPG -0.95

SPPL2A -0.95

SIRPB2 -0.95

BHLHB2 -0.95

TP53I3 -0.95

ABCA7 -0.94

DTX3L -0.94

H6PD -0.94

CD96 -0.94

GPSM3 -0.93

PREX1 -0.93

KNS2 -0.93

RAC2 -0.93

SP110 -0.93

ZMYM6 -0.93

ACY3 -0.93

TAGLN -0.93

HLA-B -0.93

UNQ5783 -0.93

GDPD5 -0.92

NEK6 -0.92

JUP -0.92

ANGPTL6 -0.92

SORL1 -0.92

PTGS1 -0.92

TSPAN32 -0.92

PDE7A -0.92

SMAD7 -0.91

KYNU -0.91

CD44 -0.91

TTLL3 -0.91

RARRES3 -0.91

UNC119 -0.91

APOL5 -0.91

SLC15A2 -0.91

DBNL -0.91

ERV3 -0.91

NFKBIA -0.91

TOR1AIP1 -0.91

TINF2 -0.9

AHNAK -0.9

ZPBP2 -0.9

CNN2 -0.9

ZNF81 -0.9

TGFBR2 -0.9

GPR174 -0.9

ITGAL -0.9

NFKB2 -0.9

ATP2B4 -0.89

UBE2H -0.89

RASGRP4 -0.89

BTNL9 -0.89

VASP -0.89

SESN3 -0.89

LIMK1 -0.89

TCIRG1 -0.89

RNF36 -0.89

TLR10 -0.89

CD2 -0.89

NYD-SP21 -0.88

PIP5K2A -0.88

BFSP2 -0.88

TMBIM4 -0.88

CTSZ -0.88

SLC25A20 -0.88

TPP1 -0.87

USP53 -0.87

DGAT1 -0.87

OSTF1 -0.87

TNFAIP3 -0.87

ZNF585A -0.87

UBE2H -0.87

P2RY10 -0.86

CASP10 -0.86

LY86 -0.86

SNX22 -0.86

RFFL -0.86

TTN -0.86

MOBKL2A -0.86

NOXA1 -0.85

GSDML -0.85

BTN2A2 -0.85

NOD27 -0.84

RAB27A -0.84

OR5B21 -0.84

PNOC -0.84

CCDC24 -0.84

WIPI1 -0.84

AMPD3 -0.84

SP110 -0.83

ARHGAP25 -0.83

NALP1 -0.83

INPP5D -0.83

TNR -0.83

IRF7 -0.83

ZFAND3 -0.83

GGT1 -0.83

CG018 -0.83

SLIC1 -0.83

PCDHGB3 -0.83

PHF20L1 -0.83

MST1 -0.83

CTSB -0.83

STAT2 -0.83

CECR1 -0.83

PTK2B -0.83

CENTB2 -0.83

ILK -0.82

COP1 -0.82

ATP7A -0.82

TMC8 -0.82

CFLAR -0.82

MSN -0.82

RGS1 -0.82

LRCH4 -0.82

TLE3 -0.82

CD80 -0.82

ZNF274 -0.82

FRRS1 -0.82

MORC3 -0.82

ZNF610 -0.82

PCDHGB5 -0.82

CYSLTR1 -0.82

CYBA -0.82

IL24 -0.82

MICAL1 -0.82

ARSA -0.82

ERBB2IP -0.82

LST1 -0.81

TCP11L2 -0.81

SIDT2 -0.81

GTF2IRD2 -0.81

TAPBP -0.81

ARSG -0.81

GPR162 -0.81

DNASE1 -0.81

PILRA -0.81

RAB7L1 -0.81

PPM1K -0.81

TTYH3 -0.81

STK17A -0.81

TRIM38 -0.81

ATP6V1G2 -0.8

NOD3 -0.8

ZNF397 -0.8

TNFSF4 -0.8

BCL3 -0.8

NRCAM -0.8

GIMAP8 -0.8

ZNF224 -0.8

BLR1 -0.8

WIG1 -0.8

HSPC049 -0.8

ALDH2 -0.8

CCM2 -0.8

DRCTNNB1A -0.8

ARHGEF1 -0.8

PIK3R1 -0.8

PKD1L2 -0.8

TCIRG1 -0.8

DOPEY2 -0.8

TLR6 -0.8

NYREN18 -0.79

KLK1 -0.79

SLC44A5 -0.79

FOXO1A -0.79

CD79B -0.79

LPXN -0.79

CD79A -0.79

ZNF447 -0.79

RNASET2 -0.79

HIP1R -0.79

IRF5 -0.79

CMTM6 -0.79

ALS2CR15 -0.78

TNFRSF13C -0.78

TAGAP -0.78

SS18 -0.78

CD82 -0.78

KCNN3 -0.77

LILRB3 -0.77

ILDR1 -0.77

MTMR1 -0.77

ICOSLG -0.77

EFEMP2 -0.77

NAPSA -0.77

PCDHGA11 -0.77

GALNAC4S -0.77

CENTD1 -0.77

SIPA1 -0.77

CPLX3 -0.77

MSRB2 -0.77

CALML4 -0.77

ATG16L2 -0.76

TA-NFKBH -0.76

PHF11 -0.76

NOTCH2NL -0.76

GTF2IRD2B -0.76

IL12RB1 -0.76

EVC2 -0.76

ACP5 -0.76

RASSF5 -0.76

OASL -0.76

POU2F2 -0.76

LAX1 -0.76

RNASEL -0.76

DDX26B -0.76

PLCB2 -0.76

GSDMDC1 -0.76

HDAC4 -0.76

DNAJC4 -0.76

CDC42SE1 -0.75

RAB11FIP1 -0.75

SP140 -0.75

CENTB1 -0.75

SORBS2 -0.75

TP53INP1 -0.75

TMEPAI -0.75

CCDC25 -0.75

KCNC3 -0.75

CUGBP2 -0.74

FES -0.74

DENND1C -0.74

GDAP2 -0.74

CDA08 -0.74

CBFA2T3 -0.74

ARPC4 -0.74

ALS2CR8 -0.74

OSBPL2 -0.74

PHOX2B -0.74

CCR6 -0.74

IL10RA -0.74

VNN2 -0.74

ATP2B1 -0.73

ANKDD1A -0.73

AP1G2 -0.73

CAPN3 -0.73

NPR2 -0.73

STIM1 -0.73

REL -0.73

CCNDBP1 -0.73

U2AF1L4 -0.73

PCDHGA9 -0.73

GBGT1 -0.73

BTG1 -0.73

APH1B -0.73

ARHGEF11 -0.73

LYN -0.73

PRMT2 -0.73

AP1GBP1 -0.72

JMJD2C -0.72

ISGF3G -0.72

CDC14A -0.72

CCL22 -0.72

TTC21A -0.72

ZNF655 -0.72

BAIAP2L1 -0.72

LGP2 -0.72

B2M -0.72

CCPG1 -0.72

PRAM1 -0.72

IQGAP1 -0.72

EFHC1 -0.72

VAMP4 -0.71

RAB24 -0.71

MSL3L1 -0.71

NUMB -0.71

PPP1R16B -0.71

MIDN -0.71

MAGED2 -0.71

RABEP2 -0.71

RAPGEF1 -0.71

SYK -0.71

BIK -0.71

HECW2 -0.71

NR3C1 -0.71

NAGK -0.71

PSCDBP -0.71

PLEKHQ1 -0.71

MAPKAPK2 -0.71

TCL6 -0.71

SEMA4A -0.7

NR4A3 -0.7

OR1L8 -0.7

AICDA -0.7

LCP1 -0.7

CNN2 -0.7

LRRC25 -0.7

GAB3 -0.7

ARP10 -0.7

RALB -0.7

TRIM54 -0.7

STAG3 -0.7

FOXP4 -0.7

SOS1 -0.7

PIK3R5 -0.7

PECAM1 -0.7

IRAK4 -0.69

ZNF137 -0.69

GRAP2 -0.69

CALCOCO1 -0.69

NEDD9 -0.69

ZNF641 -0.69

TAPBP -0.69

ARL11 -0.69

PIK3CD -0.69

ZNF333 -0.69

ZNRF2 -0.69

OR10G9 -0.69

ELMO1 -0.68

CPNE5 -0.68

CCDC32 -0.68

ATM -0.68

CTSS -0.68

NPC2 -0.68

CX3CR1 -0.68

AKAP13 -0.68

PLEC1 -0.68

CTBS -0.68

CHFR -0.68

REPS2 -0.68

CCDC50 -0.68

ZNF345 -0.68

CDGAP -0.68

SMPD1 -0.68

CBL -0.67

PLA2G4B -0.67

DIP -0.67

JAK1 -0.67

NFIX -0.67

DDX17 -0.67

RUNX1 -0.67

ZNF585A -0.67

TRAM1 -0.67

SNTB1 -0.67

SYVN1 -0.67

CTNNA1 -0.67

ZNF700 -0.67

PICALM -0.67

FCRL2 -0.67

PLEKHO1 -0.67

GPR132 -0.67

CC2D1B -0.67

S100A11 -0.67

RASA1 -0.67

HMHA1 -0.66

FUT7 -0.66

ITGAX -0.66

RASGRP2 -0.66

PLEKHG2 -0.66

ZNF217 -0.66

EPS15 -0.66

RASSF2 -0.66

PLAC8 -0.66

SLC9A9 -0.66

CAP1 -0.66

PTPRE -0.66

GMIP -0.66

STAM2 -0.66

DUS2L -0.66

KRT17 -0.66

DIP2A -0.66

FAM98C -0.66

DIP2B -0.66

POU2AF1 -0.66

APOBEC3F -0.66

ZNF655 -0.66

ARHGAP26 -0.66

BTG2 -0.65

TMEM116 -0.65

FLNA -0.65

HYPE -0.65

TRAPPC6B -0.65

GRK6 -0.65

GH2 -0.65

ZFP106 -0.65

MAP4K4 -0.65

CTSH -0.65

OR10G3 -0.65

GCC2 -0.65

RABGAP1L -0.65

ELF4 -0.65

BTN2A1 -0.65

NCOA2 -0.65

IDS -0.65

LRAP -0.65

ARID5A -0.65

FCGR2B -0.65

LGALS9 -0.64

POLB -0.64

IDUA -0.64

ARRDC1 -0.64

HSH2D -0.64

IFNAR2 -0.64

MTMR3 -0.64

P4HA1 -0.64

MARCKS -0.64

NUDT18 -0.64

FURIN -0.64

DCUN1D3 -0.64

CGGBP1 -0.64

DEGS1 -0.64

SP140 -0.64

CSK -0.64

HPSE2 -0.64

USF1 -0.64

TMEM66 -0.64

TAPBPL -0.64

AKAP11 -0.64

NAV1 -0.64

GALC -0.64

RB1 -0.64

SCPEP1 -0.64

TMEM50B -0.64

AXUD1 -0.64

PML -0.64

CD19 -0.64

TAP2 -0.63

C1QL1 -0.63

FCGRT -0.63

DENND2D -0.63

INADL -0.63

DOCK8 -0.63

CCNDBP1 -0.63

ACADS -0.63

GGA2 -0.63

CHMP5 -0.63

GORASP1 -0.63

MYH9 -0.63

PBX3 -0.63

CNOT8 -0.63

STARD10 -0.63

DET1 -0.63

SH2D3C -0.63

CYB5R4 -0.63

GFRA1 -0.63

ARHGAP21 -0.62

ZNF429 -0.62

LRRC8C -0.62

JARID1B -0.62

TAX1BP1 -0.62

ALS2CR13 -0.62

CACNA1A -0.62

TAS2R48 -0.62

USP32 -0.62

WWP2 -0.62

NRBP2 -0.62

SNAP23 -0.62

MYO1E -0.62

MYO1C -0.62

TRIM5 -0.62

PHACTR1 -0.62

CLCN3 -0.62

SNCB -0.62

TAX1BP3 -0.62

CD86 -0.62

ARHGEF7 -0.62

PARP8 -0.62

LTK -0.62

ANK1 -0.62

CEBPA -0.62

FAM107B -0.62

MAP2K6 -0.62

SH3BGRL3 -0.62

ANXA1 -0.62

MTHFR -0.62

SGCA -0.62

SLC12A6 -0.62

PFN4 -0.61

PAN3 -0.61

MARK2 -0.61

PML -0.61

MEF2A -0.61

PML -0.61

CD22 -0.61

ITPR1 -0.61

CHRM2 -0.61

TRPV2 -0.61

DNAJC10 -0.61

TTN -0.61

DEPDC5 -0.61

COL1A1 -0.61

VIM -0.61

SEMA4B -0.61

TMEM8 -0.61

NCOA7 -0.61

BTN2A2 -0.61

MOBKL2C -0.61

MOCS2 -0.61

TGFB3 -0.61

OR2D3 -0.61

SNX19 -0.61

RREB1 -0.61

DDX58 -0.61

RASA1 -0.61

PLEKHA7 -0.6

ZNF568 -0.6

SERINC3 -0.6

PDLIM5 -0.6

NBR2 -0.6

ZNF226 -0.6

ITGB7 -0.6

PIK3CG -0.6

GPR65 -0.6

TMPRSS3 -0.6

SLC45A3 -0.6

UNC84B -0.6

USP3 -0.6

CASP8 -0.6

ZNF586 -0.6

LENG1 -0.6

FYTTD1 -0.6

ZNF211 -0.6

PRF1 -0.6

PCDHGB2 -0.6

RIN3 -0.6

LNK -0.6

SLC16A13 -0.6

TLE6 -0.6

ST6GAL1 -0.59

TTC14 -0.59

TMEM30A -0.59

BTN3A3 -0.59

RASA4 -0.59

CYBASC3 -0.59

ARSB -0.59

HLA-G -0.59

OBFC2A -0.59

BTN3A2 -0.59

APOL3 -0.59

ASAH3L -0.59

PSCD4 -0.59

AHSA2 -0.59

PTAFR -0.59

BIRC2 -0.59

PILRA -0.59

CLK1 -0.59

HERC4 -0.59

STAMBPL1 -0.59

RNF122 -0.59

ZHX2 -0.59

CD83 -0.59

RTN2 -0.59

GIT2 -0.59

H2AFY -0.59

KCNMB3 -0.59

DNAJB2 -0.59

MAP3K12 -0.59

OLFML2A -0.59

HDAC7A -0.58

PCDHGB6 -0.58

MT1F -0.58

RGS3 -0.58

IL2RG -0.58

SCAP1 -0.58

PSEN1 -0.58

RAI2 -0.58

OPTN -0.58

TRIM44 -0.58

GSTM4 -0.58

RORA -0.58

MXD3 -0.58

PCDHB16 -0.58

MYD88 -0.58

ZNF528 -0.58

AHNAK -0.58

ZBED2 -0.58

CLN5 -0.58

ARHGAP18 -0.58

PLEKHM1 -0.58

CYB561D2 -0.58

A4GALT -0.58

RAB4B -0.58

SEC61A2 -0.58

FNDC5 -0.58

ARID4B -0.57

ZNF274 -0.57

MT2A -0.57

LNPEP -0.57

IFITM3 -0.57

CPT1B -0.57

FBXW4 -0.57

CASP8 -0.57

HIST1H2BK -0.57

PTER -0.57

PSD4 -0.57

FCRL6 -0.57

TRIB2 -0.57

ZSCAN2 -0.57

CCR1 -0.57

ZDHHC24 -0.57

BRIP1 -0.57

WAS -0.57

MRPL39 -0.57

MYO9B -0.57

ITPKB -0.57

HERC3 -0.57

PTPN1 -0.57

TMEM63A -0.57

ARID4A -0.57

PJA2 -0.57

LY9 -0.57

PHF20L1 -0.57

HIPK1 -0.57

SLC41A2 -0.57

ZNF548 -0.57

BTN2A1 -0.57

MCEMP1 -0.57

SMAD3 -0.57

CNOT4 -0.56

ZPBP2 -0.56

TCF4 -0.56

ZNF720 -0.56

CNR1 -0.56

MBD6 -0.56

SCRN1 -0.56

REEP3 -0.56

BCL10 -0.56

GLT1D1 -0.56

RASSF5 -0.56

PRKCBP1 -0.56

ARHGEF2 -0.56

PTPRT -0.56

PLAU -0.56

RAB11FIP1 -0.56

MGLL -0.56

DTX2 -0.56

ERBB2 -0.56

WDR26 -0.56

LAT2 -0.56

TRIM8 -0.56

FAM111A -0.56

IER5 -0.56

DDX59 -0.56

ZNF256 -0.56

PCDHGC3 -0.56

SLC15A4 -0.56

RNF36 -0.56

TREML1 -0.56

NMI -0.56

PDCD6IP -0.56

CENTD1 -0.56

DRD3 -0.56

CLEC2D -0.56

FBXO32 -0.56

NCOA1 -0.56

USP52 -0.55

RABIF -0.55

TBC1D22A -0.55

DUSP18 -0.55

ZNF438 -0.55

CREM -0.55

EPM2AIP1 -0.55

RUNDC2A -0.55

PQLC3 -0.55

SIRPB2 -0.55

P2RX1 -0.55

OMA1 -0.55

ZNF264 -0.55

WDFY1 -0.55

CRB2 -0.55

ORAOV1 -0.55

YES1 -0.55

PCNXL2 -0.55

KALRN -0.55

ACADVL -0.55

PBXIP1 -0.55

TESK2 -0.55

ITGB3 -0.55

MPZ -0.55

ITM2C -0.55

MARVELD1 -0.55

DAZL -0.55

VASP -0.55

RIPK5 -0.55

ANKRD23 -0.55

ZNF83 -0.55

PGLYRP4 -0.55

PTP4A3 -0.54

IDS -0.54

CAST -0.54

LEPR -0.54

FOS -0.54

PARP14 -0.54

S100PBP -0.54

GIMAP1 -0.54

NR4A3 -0.54

AP1B1 -0.54

SNRP70 -0.54

TAS2R45 -0.54

DIP2A -0.54

CEPT1 -0.54

POLA -0.54

PIGV -0.54

SSH2 -0.54

HEM1 -0.54

RORA -0.54

ZBTB37 -0.54

PLAG1 -0.54

PDE7A -0.54

C1QL2 -0.54

HNRPUL1 -0.54

OGFRL1 -0.54

NECAP2 -0.54

KLHL9 -0.54

KCNV2 -0.54

RASGRP4 -0.54

CSH1 -0.54

FGFR1OP2 -0.53

IGF2BP1 -0.53

SS18 -0.53

ABI1 -0.53

ENTPD4 -0.53

KLHL5 -0.53

TRIM21 -0.53

RBM5 -0.53

LRRIQ2 -0.53

PTGES -0.53

TYROBP -0.53

SH3BGRL -0.53

KBTBD2 -0.53

OXER1 -0.53

FBXL19 -0.53

ZNF671 -0.53

ACTB -0.53

CGGBP1 -0.53

STX5A -0.53

PSMB9 -0.53

06-Sep -0.53

ZNF611 -0.53

CCDC33 -0.53

PIGA -0.53

CAMK1D -0.53

PPBP -0.53

TERF1 -0.53

STX17 -0.53

SLC19A2 -0.53

PSEN1 -0.53

FBLN5 -0.53

LY9 -0.53

CHST11 -0.53

OGG1 -0.53

SFTPA2 -0.53

EDG4 -0.53

ZNF431 -0.53

PIP3-E -0.52

SDCCAG8 -0.52

DHRS1 -0.52

STX12 -0.52

ANP32A -0.52

NOL3 -0.52

AER61 -0.52

YIPF1 -0.52

SERINC3 -0.52

MAN1A2 -0.52

NDE1 -0.52

TRERF1 -0.52

ARHGDIB -0.52

CELSR3 -0.52

CD248 -0.52

LYSMD2 -0.52

TAF11 -0.52

GYS1 -0.52

MCL1 -0.52

NPAL3 -0.52

LDHAL6A -0.52

SPATS2 -0.52

ENG -0.52

IMPACT -0.52

GIMAP2 -0.52

HERPUD1 -0.52

PRKCD -0.52

ADORA2A -0.52

TXNDC6 -0.51

ERGIC1 -0.51

NDUFC2 -0.51

KCNH3 -0.51

BATF2 -0.51

ANK2 -0.51

CORO1B -0.51

JARID1A -0.51

TPST1 -0.51

WDR44 -0.51

RFX5 -0.51

TBC1D3 -0.51

ATP2A3 -0.51

HTATIP -0.51

CAV3 -0.51

GLP1R -0.51

DDX17 -0.51

STRN -0.51

ZC3H12A -0.51

LRP10 -0.51

METTL7B -0.51

TRIM56 -0.51

GNAI3 -0.51

FFAR1 -0.51

CSNK1A1L -0.51

INPP4A -0.51

ZFYVE1 -0.51

7A5 -0.51

SKI -0.51

RAP2C -0.51

LST1 -0.51

KRTAP5-1 -0.51

RAB6IP1 -0.51

CYGB -0.5

CD99 -0.5

GNS -0.5

CRLF3 -0.5

UBE2W -0.5

ZMAT5 -0.5

JPH2 -0.5

BAT5 -0.5

SLC39A7 -0.5

CTDSP2 -0.5

FOXG1B -0.5

GCA -0.5

EVL -0.5

TBC1D2B -0.5

PDE4DIP -0.5

LIPA -0.5

MARVELD2 -0.5

MFGE8 -0.5

ADAM17 -0.5

LRRC15 -0.5

SLC1A2 -0.5

AFF4 -0.5

SH3KBP1 -0.5

PHKB -0.5

CLSTN3 -0.5

COQ4 -0.5

NY-REN-7 -0.5

MYL6 -0.5

LRCH3 -0.5

PNCK -0.5

TPCN1 -0.5

BTLA -0.5

TNFRSF19L -0.5

MYL4 -0.5

TRIP4 -0.5

FKSG30 -0.5

RTP4 -0.5

DLG5 -0.5

CHEK2 -0.5

DMPK -0.5

HHLA3 -0.5

P2RY8 -0.5

ODF2L -0.5

ATP6V0A1 -0.5

EML1 -0.5
