# Supplementary material for: Time-Dependent c-Myc Transactomes Mapped by Array-Based Nuclear Run-On Reveal Transcriptional Modules in Human B Cells
Source: PLoS One. 2010 Mar 15;5(3):e9691. doi: 10.1371/journal.pone.0009691 (PMC2837740; doi:10.1371/journal.pone.0009691)
Supplement: Table S4 — (0.40 MB DOC) [file pone.0009691.s005.doc]

**Table S4. Enrichment of transcription factor binding motifs at different timepoints determined by GSMA**

|  | Label | NRO 15' | NRO 30' | NRO 1hr | NRO 3hr | NRO 6hr | NRO 48hr |
| --- | --- | --- | --- | --- | --- | --- | --- |
| 15 mins | V-AP1FJ-Q2 | 2.535674 | 0.872239 | 2.16972 | 1.843443 | 0.576064 | -3.85648 |
| 15 mins | V-AP1-Q6 | 2.423475 | 0.833644 | 2.229099 | 2.286969 | -0.71817 | -3.91216 |
| 15 mins | V-CDP-02 | 2.349966 | 0.81754 | 0.031653 | -1.28269 | -0.61022 | -2.83931 |
| 15 mins | V-CEBPB-02 | 2.278853 | 0.783896 | 0.927176 | -0.07142 | -0.67532 | -3.6787 |
| 15 mins | V-CHOP-01 | 2.523454 | 0.291727 | 1.026695 | -0.62585 | -1.07807 | -4.70198 |
| 15 mins | V-COUP-DR1-Q6 | 2.292378 | 0.265013 | 0.638719 | 0.673188 | -0.07927 | -3.27237 |
| 15 mins | V-CREB-Q2 | 2.535674 | 2.319987 | 1.356825 | 0.05788 | 0.078256 | -3.93541 |
| 15 mins | V-CREB-Q3 | 3.20533 | 1.940594 | 2.229099 | 1.761874 | 1.184948 | -3.30863 |
| 15 mins | V-DR1-Q3 | 2.292378 | 0.788548 | 1.520598 | 1.418222 | 0.220762 | -3.41509 |
| 15 mins | V-E4F1-Q6 | 2.146999 | 3.219149 | 2.847023 | 1.583754 | 0.581576 | -3.33556 |
| 15 mins | V-FOXD3-01 | 2.547338 | 0.222656 | 0.907095 | -0.99929 | -2.33527 | -4.66789 |
| 15 mins | V-FOXM1-01 | 2.904412 | 0.755381 | 1.175045 | 0.050125 | 0.642591 | -4.36519 |
| 15 mins | V-FOXO1-01 | 2.450139 | 0.484827 | 1.670042 | -0.40897 | -2.14396 | -4.98206 |
| 15 mins | V-FOXO1-02 | 2.167622 | 0.745634 | 2.132748 | 0.049478 | -0.5005 | -4.84867 |
| 15 mins | V-FOXO3-01 | 2.004626 | -0.22607 | 1.072664 | -0.28 | 0.455418 | -5.1081 |
| 15 mins | V-FOXO4-01 | 2.167622 | 0.745634 | 2.410709 | -0.77242 | -0.64235 | -4.57877 |
| 15 mins | V-FOXO4-02 | 2.82896 | 1.224243 | 2.378778 | 0.396401 | 0.345954 | -5.25053 |
| 15 mins | V-FOX-Q2 | 2.278853 | 0.783896 | 1.657739 | 1.162975 | 0.21946 | -4.81373 |
| 15 mins | V-FREAC2-01 | 2.450139 | 0.725747 | 1.670042 | 0.391008 | -0.34909 | -4.45664 |
| 15 mins | V-GATA2-01 | 2.18141 | 1.509021 | 2.257269 | 1.556413 | 0.266765 | -3.02184 |
| 15 mins | V-GNCF-01 | 2.132779 | 1.610425 | 1.691787 | 1.727251 | 0.955476 | -1.68209 |
| 15 mins | V-HFH3-01 | 2.731716 | 0.710466 | 2.429434 | 1.277789 | -0.07142 | -4.61999 |
| 15 mins | V-HFH4-01 | 2.589446 | -0.22079 | 1.424205 | 1.105191 | -0.0677 | -5.17167 |
| 15 mins | V-HNF3B-01 | 2.416617 | 0.21123 | 2.266354 | 1.427314 | 0.295529 | -5.28152 |
| 15 mins | V-HNF3-Q6 | 2.160819 | 0.640048 | 1.114938 | 0.647201 | -0.55139 | -4.10416 |
| 15 mins | V-HNF4-01 | 2.345696 | 1.342601 | 2.458355 | 1.451208 | -0.08111 | -3.93264 |
| 15 mins | V-HSF-Q6 | 2.751439 | 0.478046 | 1.913448 | 2.188492 | 1.289425 | 2.470076 |
| 15 mins | V-MEF2-01 | 2.554682 | 0.534135 | 1.826471 | 0.119554 | -0.05369 | -4.34341 |
| 15 mins | V-NKX62-Q2 | 2.711848 | 0.705299 | 1.754452 | 1.490622 | 0.86834 | -4.07577 |
| 15 mins | V-PAX4-04 | 2.483935 | -0.7297 | -0.08974 | -0.53047 | -1.75362 | -4.51812 |
| 15 mins | V-PPAR-DR1-Q2 | 2.397829 | 0.824822 | 1.590546 | 1.483461 | -0.08291 | -4.02004 |
| 15 mins | V-PPARG-01 | 2.678891 | 1.523309 | -0.50313 | 0.189996 | 1.574674 | 4.536247 |
| 15 mins | V-SMAD4-Q6 | 2.223933 | 2.288715 | 3.186292 | 1.978201 | 0.650777 | -5.18232 |
| 15 mins | V-SOX5-01 | 3.014051 | -0.51722 | 0.342726 | -1.67614 | -2.01748 | -4.67185 |
| 15 mins | V-TATA-01 | 2.345829 | 0.694849 | 0.433309 | -0.06331 | 0.062341 | -2.94641 |
| 15 mins | V-TAXCREB-01 | 2.863536 | 2.1887 | 0.708143 | -0.80878 | -0.51578 | -3.35146 |
| 15 mins | V-TCF11MAFG-01 | 2.065386 | 0.710466 | 1.370028 | 0.159022 | -0.07142 | -3.07693 |
| 15 mins | V-ZF5-B | 2.769608 | 0.27571 | 3.111093 | 1.733832 | -0.23854 | -3.99837 |
| 30 mins | V-AP4-Q5 | 1.506454 | 2.034763 | 1.760379 | -0.0697 | -2.40547 | -4.9054 |
| 30 mins | V-ATF-01 | 0.877679 | 3.111041 | 0.689316 | -0.61357 | -0.40934 | -3.68562 |
| 30 mins | V-ATF1-Q6 | 1.525168 | 3.345573 | 2.648424 | 0.661201 | 0.806205 | -4.26545 |
| 30 mins | V-ATF3-Q6 | 1.561925 | 2.109687 | 2.268725 | -0.07227 | -0.38152 | -4.15291 |
| 30 mins | V-ATF-B | 1.347413 | 2.501371 | 0.171618 | 0.045405 | -0.45929 | -3.89216 |
| 30 mins | V-CREB-02 | 1.684267 | 2.274934 | 1.489898 | 0.864858 | 1.053017 | 3.26191 |
| 30 mins | V-CREBP1-Q2 | 1.388882 | 2.812487 | 2.017377 | 0.046802 | -0.0709 | -3.1822 |
| 30 mins | V-CREB-Q4 | 1.692667 | 2.000938 | 1.016678 | -0.07832 | -0.08641 | 2.967031 |
| 30 mins | V-LMO2COM-01 | 1.439038 | 2.671466 | 3.043701 | 2.004748 | 0.482631 | -4.48749 |
| 30 mins | V-PAX4-01 | -0.76434 | 2.107084 | 4.782369 | 1.342888 | 0.25341 | -4.2478 |
| 30 mins | V-SOX9-B1 | 1.543656 | 2.085012 | 0.634951 | 0.052017 | -1.86835 | -4.24621 |
| 30 mins | V-SPZ1-01 | 0.446077 | 2.636293 | 2.268725 | 1.676341 | -0.23063 | -4.5118 |
| 1 hour | V-AHRARNT-01 | 0.058739 | 1.254443 | 2.383504 | 0.635539 | -0.66103 | -3.46142 |
| 1 hour | V-AML1-01 | 0.074985 | 0.802343 | 2.743607 | 1.190343 | -1.30176 | -5.7257 |
| 1 hour | V-AML1-Q6 | 0.074985 | 0.802343 | 2.743607 | 1.190343 | -1.30176 | -5.7257 |
| 1 hour | V-AML-Q6 | 0.074985 | 0.802343 | 2.294954 | 1.443032 | -0.08065 | -5.50787 |
| 1 hour | V-AP1-Q4 | 1.758426 | 0.89296 | 2.054823 | 1.606009 | -0.08976 | -4.19052 |
| 1 hour | V-AP2ALPHA-01 | -0.71861 | 0.851013 | 2.910035 | 1.530567 | 0.23825 | -3.83964 |
| 1 hour | V-AP2GAMMA-01 | 0.903884 | 1.458299 | 3.323638 | 1.852285 | 0.245363 | -4.11291 |
| 1 hour | V-AP2-Q6-01 | -0.71861 | 1.41602 | 2.910035 | 2.870654 | 0.562046 | -4.30172 |
| 1 hour | V-AP4-01 | 0.066398 | 0.238771 | 3.223989 | 1.725296 | -0.34174 | -3.84846 |
| 1 hour | V-AREB6-01 | 0.06294 | 0.673464 | 2.051849 | 0.25679 | -0.45206 | -4.13559 |
| 1 hour | V-AREB6-03 | -2.91829 | 1.32743 | 2.430576 | 2.188558 | 0.223344 | -4.61015 |
| 1 hour | V-CACCCBINDINGFACTOR-Q6 | 0.072384 | 1.288721 | 2.070973 | 1.392969 | 0.658861 | -4.26545 |
| 1 hour | V-CDC5-01 | 0.808458 | 0.783896 | 2.82664 | 1.656734 | 0.21946 | -5.59406 |
| 1 hour | V-CHX10-01 | -0.55944 | 0.662513 | 2.265459 | 1.713173 | 1.697925 | -3.10907 |
| 1 hour | V-COMP1-01 | 0.046609 | 1.326503 | 2.263124 | 1.368163 | 0.993519 | -2.34042 |
| 1 hour | V-COREBINDINGFACTOR-Q6 | 0.92935 | 1.499386 | 2.745435 | 1.620676 | -0.43344 | -6.6752 |
| 1 hour | V-CP2-01 | 0.070596 | -0.24765 | 2.723818 | 1.953317 | -0.07593 | -4.36519 |
| 1 hour | V-CREL-01 | 0.92935 | 1.499386 | 2.409512 | 1.053083 | -1.11916 | -6.6752 |
| 1 hour | V-DBP-Q6 | -1.43001 | -0.26304 | 2.145403 | 1.190343 | 0.224624 | -4.56396 |
| 1 hour | V-E12-Q6 | -1.31134 | 0.247271 | 2.515918 | 1.786714 | -0.07396 | -4.65128 |
| 1 hour | V-E2A-Q2 | -0.25692 | -0.22433 | 2.849906 | 2.092598 | -0.97998 | -4.016 |
| 1 hour | V-E2F-03 | 1.7904 | 0.30556 | 2.092187 | 1.921553 | -0.09139 | 7.58138 |
| 1 hour | V-E2F1-Q4 | 1.742219 | 0.297338 | 2.035884 | 1.869843 | 0.584313 | 4.334917 |
| 1 hour | V-E2F1-Q6-01 | 0.086831 | 0.312247 | 2.137973 | 1.963605 | 1.320622 | 7.495057 |
| 1 hour | V-E47-01 | 0.822769 | 0.797771 | 3.025372 | 0.932311 | 0.223344 | -4.61015 |
| 1 hour | V-E47-02 | -0.62963 | 1.240677 | 2.54969 | 0.636551 | -1.06791 | -4.64624 |
| 1 hour | V-EGR1-01 | 0.873235 | 1.689924 | 3.842218 | 2.856119 | 2.331059 | -3.89683 |
| 1 hour | V-EGR2-01 | 0.069685 | -0.49197 | 3.105613 | 1.575868 | 1.05985 | -3.49914 |
| 1 hour | V-EGR3-01 | 0.725878 | -0.31924 | 2.466101 | 0.33686 | 0.871811 | -2.44568 |
| 1 hour | V-EGR-Q6 | -1.532 | -0.28181 | 2.618848 | 2.628794 | 2.202945 | -4.34496 |
| 1 hour | V-ELF1-Q6 | 0.958206 | 1.854364 | 2.137973 | 1.232085 | -0.4469 | -6.79838 |
| 1 hour | V-ER-Q6 | 0.07667 | 0.820375 | 2.19362 | 1.217096 | -0.08247 | -4.07261 |
| 1 hour | V-ER-Q6-01 | 1.667339 | 1.127777 | 2.106202 | 0.722836 | -1.05158 | -4.50981 |
| 1 hour | V-ER-Q6-02 | 1.552817 | 1.835618 | 2.696436 | 1.418222 | 0.220762 | -3.98597 |
| 1 hour | V-ERR1-Q2 | 0.903884 | 0.294546 | 2.016768 | 1.300246 | 0.578827 | -3.79566 |
| 1 hour | V-ETS1-B | 0.903884 | 1.458299 | 2.343485 | 2.128305 | -1.08849 | -5.69916 |
| 1 hour | V-ETS2-B | 0.950051 | 1.838582 | 3.493398 | 1.656775 | -0.79359 | -6.40707 |
| 1 hour | V-ETS-Q4 | 0.954138 | 0.925149 | 2.128894 | 1.372536 | -1.50101 | -6.85324 |
| 1 hour | V-FOXJ2-01 | 1.719623 | 0.705299 | 2.674691 | 2.157001 | 1.405048 | -4.71404 |
| 1 hour | V-FREAC3-01 | 1.973545 | 1.129593 | 2.827553 | 1.434774 | 0.964957 | -4.41455 |
| 1 hour | V-FXR-IR1-Q6 | -0.42113 | 0.995391 | 2.170166 | 1.28963 | 0.424255 | -2.43069 |
| 1 hour | V-GATA1-02 | 1.487504 | 1.256896 | 2.01983 | 0.288024 | -0.50705 | -5.45893 |
| 1 hour | V-GATA1-05 | -1.32017 | -0.24284 | 2.256733 | 1.098908 | -1.4836 | -4.54854 |
| 1 hour | V-GATA3-01 | 1.378632 | 0.235286 | 2.393966 | 1.700108 | 0.995119 | -4.55254 |
| 1 hour | V-GR-Q6-01 | 1.399057 | 0.238771 | 2.164583 | 0.830282 | -0.61206 | -3.84846 |
| 1 hour | V-HEB-Q6 | 0.065429 | 0.700094 | 2.393966 | 1.700108 | -0.60312 | -4.42583 |
| 1 hour | V-HEN1-01 | 0.700146 | -0.22257 | 3.080628 | 1.434774 | -0.06824 | -3.67733 |
| 1 hour | V-HEN1-02 | 0.39078 | 0.925513 | 2.635063 | 0.812032 | -0.06985 | -3.88963 |
| 1 hour | V-HFH1-01 | 1.817966 | 0.003069 | 2.827651 | 1.810698 | -0.7842 | -4.71372 |
| 1 hour | V-HFH8-01 | 1.66828 | 1.138523 | 2.722368 | 0.58414 | 0.19156 | -5.31634 |
| 1 hour | V-HIF1-Q3 | 0.07541 | 0.271177 | 2.759152 | 2.467692 | 2.374947 | 2.93124 |
| 1 hour | V-HIF1-Q5 | 0.822769 | 1.857088 | 2.430576 | 1.937308 | 0.83042 | -3.45503 |
| 1 hour | V-HMX1-01 | -1.41193 | 0.796403 | 2.126409 | 0.076576 | 0.09578 | -1.63648 |
| 1 hour | V-HNF4ALPHA-Q6 | 1.632957 | 1.104522 | 2.062771 | 1.491415 | 0.389912 | -3.59134 |
| 1 hour | V-IK1-01 | 0.813256 | 0.788548 | 2.990395 | 1.666566 | 0.520791 | -4.84229 |
| 1 hour | V-IK2-01 | 1.477938 | 1.248813 | 2.286625 | 1.586202 | -0.93213 | -5.01631 |
| 1 hour | V-IK3-01 | 0.665971 | 1.074458 | 2.68954 | 0.55127 | -0.06491 | -3.84846 |
| 1 hour | V-LFA1-Q6 | -1.39713 | 0.26345 | 3.118865 | 2.273933 | -0.67532 | -5.23936 |
| 1 hour | V-MAZ-Q6 | 0.378753 | 1.791285 | 3.809254 | 2.059642 | -0.96454 | -3.40424 |
| 1 hour | V-MAZR-01 | -2.49993 | 0.783896 | 2.388302 | 2.273933 | -0.37706 | -4.9556 |
| 1 hour | V-MEIS1AHOXA9-01 | -1.08922 | 0.001991 | 2.646466 | 0.184483 | -0.23273 | -3.84679 |
| 1 hour | V-MSX1-01 | -1.70967 | -0.83198 | 3.555011 | 2.120118 | -0.66103 | -4.31459 |
| 1 hour | V-MTF1-Q4 | 0.827484 | 1.601384 | 3.491362 | 2.706478 | 2.056289 | -3.91049 |
| 1 hour | V-MYB-Q3 | 0.846081 | -0.26896 | 2.040708 | 0.054438 | -1.48709 | -4.36957 |
| 1 hour | V-MYB-Q6 | 0.461376 | 1.365041 | 2.040708 | 2.508937 | 1.946435 | -4.5923 |
| 1 hour | V-MYCMAX-01 | 0.083454 | 0.300104 | 2.054823 | 2.449693 | 4.327062 | 6.961133 |
| 1 hour | V-MYCMAX-B | 0.890878 | 1.150563 | 2.953797 | 2.641776 | 2.542488 | 5.092306 |
| 1 hour | V-MYOD-01 | -1.28448 | -0.71476 | 2.733054 | -0.06566 | -1.16928 | -4.55603 |
| 1 hour | V-MZF1-02 | 0.07541 | -0.80025 | 2.458355 | -0.07352 | -2.53717 | -4.80888 |
| 1 hour | V-NERF-Q2 | -1.60605 | 0.302844 | 2.073589 | 1.620676 | -1.11916 | -6.34901 |
| 1 hour | V-NF1-Q6 | 0.070142 | 0.750523 | 2.006841 | 1.586202 | -0.36101 | -4.47295 |
| 1 hour | V-NFAT-Q4-01 | 0.481017 | 0.287447 | 2.446429 | 1.134226 | 0.564877 | -5.25221 |
| 1 hour | V-NFE2-01 | 0.077086 | 0.277204 | 3.127955 | 2.002998 | 0.544748 | -4.4679 |
| 1 hour | V-NFKAPPAB65-01 | 0.855228 | 1.930353 | 2.217336 | 1.230254 | -0.39887 | -5.84263 |
| 1 hour | V-NFKB-C | -0.74358 | -0.2887 | 2.026348 | 1.583754 | -0.42357 | -6.68251 |
| 1 hour | V-NFKB-Q6 | 0.081517 | 1.451338 | 2.820036 | 1.568741 | 2.235422 | -5.59302 |
| 1 hour | V-NFKB-Q6-01 | 0.075832 | 1.350122 | 4.287018 | 1.970427 | -0.08156 | -5.7904 |
| 1 hour | V-NGFIC-01 | 0.8821 | 0.287447 | 3.402961 | 1.942329 | 1.215731 | -3.93639 |
| 1 hour | V-NKX61-01 | -0.61283 | 0.243907 | 2.752234 | 1.076706 | 0.893515 | -4.06258 |
| 1 hour | V-NMYC-01 | 0.941826 | -0.29939 | 2.101424 | 1.642431 | 2.687889 | 4.226542 |
| 1 hour | V-NRF2-Q4 | 1.606688 | 0.274207 | 2.181666 | 2.495264 | 1.159733 | 2.81632 |
| 1 hour | V-OLF1-01 | 0.073261 | 0.26345 | 3.118865 | 2.150493 | 0.517718 | -4.45903 |
| 1 hour | V-OSF2-Q6 | -0.70764 | 0.003449 | 2.2408 | 0.451497 | -0.40309 | -5.3736 |
| 1 hour | V-P300-01 | -1.56204 | 0.294546 | 2.670203 | 0.472187 | -1.75542 | -5.38191 |
| 1 hour | V-P53-DECAMER-Q2 | -0.66587 | 0.788548 | 2.108517 | 1.418222 | -0.3793 | -5.5559 |
| 1 hour | V-PAX2-02 | 0.065429 | 1.62971 | 2.654951 | 0.818161 | -0.07038 | -5.69294 |
| 1 hour | V-PAX4-02 | 0.77905 | 0.755381 | 2.01983 | 0.525923 | 0.498886 | -5.73236 |
| 1 hour | V-PAX5-01 | -1.06708 | -0.19628 | 2.047286 | 1.265352 | 0.395414 | -3.45982 |
| 1 hour | V-PAX-Q6 | 1.841731 | 1.006138 | 2.01983 | 0.406974 | -0.36334 | -4.98042 |
| 1 hour | V-POU1F1-Q6 | 1.357899 | 0.689565 | 2.357964 | 1.023028 | -0.59405 | -3.61044 |
| 1 hour | V-POU6F1-01 | 1.719623 | 1.407695 | 2.674691 | 1.04637 | 0.331633 | -4.39491 |
| 1 hour | V-PXR-Q2 | 0.43558 | 0.260294 | 2.648424 | 2.002776 | 0.511518 | -4.6159 |
| 1 hour | V-RP58-01 | 0.665971 | 0.645738 | 2.448818 | 1.974846 | 1.409241 | -3.73158 |
| 1 hour | V-SP1-01 | -1.36346 | 1.018956 | 3.328883 | 2.098666 | -0.0769 | -4.4208 |
| 1 hour | V-SP1-Q6-01 | 0.073696 | -0.78206 | 2.696436 | 0.673188 | -1.27938 | -4.98502 |
| 1 hour | V-SP3-Q3 | 0.080333 | 0.859566 | 2.939282 | 2.087372 | 0.567694 | -4.81168 |
| 1 hour | V-SREBP1-Q6 | 0.077086 | -0.81803 | 2.820474 | 1.74323 | -1.9659 | -5.36362 |
| 1 hour | V-SRF-C | 0.073261 | 0.783896 | 2.680527 | 1.409855 | -1.71922 | -5.59406 |
| 1 hour | V-SRF-Q4 | 0.072824 | 1.813894 | 2.955004 | 1.401437 | -1.56072 | -5.34911 |
| 1 hour | V-SRF-Q5-01 | 0.07791 | 1.387119 | 3.472181 | 1.499327 | -1.98692 | -5.57186 |
| 1 hour | V-SRF-Q6 | 1.667339 | 0.846704 | 2.421841 | 1.522817 | -2.50128 | -5.8124 |
| 1 hour | V-STAT5A-01 | 0.073261 | 0.26345 | 3.41109 | 0.916096 | 0.666848 | -3.96246 |
| 1 hour | V-STAT5A-02 | 0.665971 | 0.217018 | 2.930262 | 0.754638 | -0.06491 | -3.38096 |
| 1 hour | V-STAT5B-01 | 0.074558 | 0.797771 | 3.025372 | 0.429812 | -0.08019 | -4.89893 |
| 1 hour | V-STAT6-02 | 1.217335 | 1.080766 | 2.472127 | 1.076021 | -1.16211 | -4.83582 |
| 1 hour | V-STAT-Q6 | 1.65881 | 0.283102 | 2.88049 | 1.780324 | 1.517864 | -4.41051 |
| 1 hour | V-T3R-Q6 | -2.04155 | 0.252234 | 2.846194 | 1.586202 | 0.495678 | -4.06544 |
| 1 hour | V-TAL1ALPHAE47-01 | -0.29081 | 0.774508 | 2.070973 | 0.53924 | -0.37254 | -5.73733 |
| 1 hour | V-TATA-C | 0.43558 | 0.774508 | 3.370237 | 2.978467 | 0.806205 | -4.75607 |
| 1 hour | V-TEF-Q6 | 1.933874 | 1.056476 | 3.155776 | 1.676341 | 1.127422 | -5.01425 |
| 1 hour | V-TEL2-Q6 | 0.084216 | 0.302844 | 2.073589 | 1.336879 | -0.7763 | -5.85973 |
| 1 hour | V-TFIIA-Q6 | -0.30467 | -0.26602 | 2.472127 | 1.970427 | -0.08156 | -4.54211 |
| 1 hour | V-TGIF-01 | 0.430235 | 1.272908 | 2.188153 | 0.050764 | 0.505241 | -4.14388 |
| 1 hour | V-TTF1-Q6 | -0.60856 | 1.199171 | 2.195729 | 1.750122 | -0.34666 | -4.03427 |
| 1 hour | V-USF-02 | -0.79117 | 0.936935 | 3.029205 | 1.980176 | 0.618792 | -3.88815 |
| 1 hour | V-VDR-Q3 | 0.071047 | 1.264927 | 2.88292 | 1.846089 | 0.212828 | -4.80585 |
| 1 hour | V-ZIC2-01 | -0.66194 | 0.523673 | 3.264977 | 2.273933 | 0.815977 | -4.24621 |
| 1 hour | V-ZIC3-01 | -1.47796 | 0.829245 | 3.453858 | 1.230254 | 0.389912 | -4.86707 |
| 3 hours | V-AP2REP-01 | -0.236 | 1.671728 | 1.797751 | 2.120118 | 0.893376 | -3.17703 |
| 3 hours | V-PEA3-Q6 | -0.73299 | 0.291727 | 1.997468 | 2.107938 | -0.41753 | -6.74437 |
| 3 hours | V-USF2-Q6 | -1.11203 | -0.55866 | 1.474924 | 2.056138 | 1.847825 | 4.30185 |
| 6 hours | V-MYC-Q2 | -1.32017 | -0.24284 | 1.70448 | 1.798746 | 3.307478 | 5.506148 |
| 6 hours | V-NRSF-01 | 1.025183 | 0.586781 | 0.942957 | 1.189564 | 2.111382 | -2.16554 |
| 6 hours | V-USF-C | 0.962258 | 0.313568 | 1.799196 | 1.971908 | 2.746201 | 4.571539 |
| 48 hours | V-AHR-01 | -0.38893 | -0.7626 | -0.31373 | -0.47714 | -1.62353 | 2.006683 |
| 48 hours | V-AHR-Q5 | 0.072824 | 0.779216 | 0.921641 | 0.419815 | 0.218149 | 2.689679 |
| 48 hours | V-AR-02 | 0.722386 | -0.68727 | -0.69884 | -1.42667 | -0.6498 | 2.068397 |
| 48 hours | V-ARNT-01 | 0.082685 | 0.297338 | 1.046441 | 0.198026 | 1.257562 | 3.053889 |
| 48 hours | V-ARNT-02 | 0.92094 | 0.89296 | 0.723292 | 0.199869 | -0.08976 | 3.082298 |
| 48 hours | V-CREB-01 | 0.877679 | 1.41602 | 0.689316 | 0.458497 | -0.08555 | 2.937507 |
| 48 hours | V-CREB-Q2-01 | 1.588934 | 1.342601 | 0.653575 | -0.07352 | 0.225897 | 3.07728 |
| 48 hours | V-E2F-01 | -0.19797 | 0.527243 | 1.016709 | -0.04804 | -0.053 | 5.875547 |
| 48 hours | V-E2F-02 | 0.92935 | -0.29543 | 0.729898 | 1.336879 | 0.252276 | 9.308024 |
| 48 hours | V-E2F1DP1-01 | 0.925155 | -0.29409 | 0.893806 | 1.330844 | 0.251137 | 9.266001 |
| 48 hours | V-E2F1DP1RB-01 | 0.490543 | -0.28596 | 1.031667 | 1.43139 | 0.410128 | 9.246551 |
| 48 hours | V-E2F1DP2-01 | 0.925155 | -0.29409 | 0.893806 | 1.330844 | 0.251137 | 9.266001 |
| 48 hours | V-E2F1-Q3 | -0.72583 | -0.28181 | 0.696244 | 1.275239 | -0.08641 | 8.87885 |
| 48 hours | V-E2F1-Q3-01 | 0.078726 | -0.83544 | 0.682318 | 0.984435 | -0.72569 | 3.669996 |
| 48 hours | V-E2F1-Q4-01 | 0.941826 | 0.913211 | 1.080128 | 1.354825 | 0.081932 | 7.119002 |
| 48 hours | V-E2F1-Q6 | 0.937686 | -0.29808 | 0.736444 | 1.34887 | 0.254539 | 9.062392 |
| 48 hours | V-E2F4DP1-01 | 1.774485 | -0.29543 | 0.729898 | 1.336879 | 0.595135 | 9.471119 |
| 48 hours | V-E2F4DP2-01 | 0.925155 | -0.29409 | 0.893806 | 1.330844 | 0.251137 | 9.266001 |
| 48 hours | V-E2F-Q3 | 0.079934 | -0.28041 | 1.011632 | 1.403594 | -0.4114 | 8.44778 |
| 48 hours | V-E2F-Q3-01 | 0.92935 | 0.901115 | 1.065821 | 1.336879 | -0.09058 | 7.187801 |
| 48 hours | V-E2F-Q4 | 0.084971 | -0.29808 | 1.07538 | 1.34887 | 0.600473 | 9.062392 |
| 48 hours | V-E2F-Q4-01 | 0.082685 | 0.88473 | 1.70607 | 1.312571 | -0.08894 | 7.377359 |
| 48 hours | V-E2F-Q6 | 0.086092 | -0.30201 | 1.089561 | 1.366658 | 0.433143 | 9.348628 |
| 48 hours | V-E2F-Q6-01 | 0.080333 | 0.859566 | 1.657546 | 1.816661 | -0.08641 | 8.412127 |
| 48 hours | V-ELK1-02 | 0.086462 | -2.14599 | -0.63016 | 0.207073 | -0.797 | 3.360851 |
| 48 hours | V-GABP-B | 0.08572 | -1.51861 | 0.059089 | 0.783025 | 1.303726 | 3.830022 |
| 48 hours | V-HTF-01 | 1.355924 | 0.586781 | 1.337345 | 1.855942 | 1.843028 | 2.238492 |
| 48 hours | V-MAX-01 | -0.78454 | 0.929094 | 1.79162 | 1.378389 | 1.320622 | 3.879657 |
| 48 hours | V-MIF1-01 | -0.21781 | -0.57532 | 0.794248 | 0.677923 | 0.714191 | 2.527311 |
| 48 hours | V-MYCMAX-03 | 1.750341 | 0.888854 | 1.051319 | 1.038755 | 1.432521 | 4.113814 |
| 48 hours | V-NFMUE1-Q6 | 0.916706 | -0.88154 | -0.27409 | -0.08099 | -0.59664 | 3.952939 |
| 48 hours | V-NRF1-Q6 | 0.846081 | 0.003377 | 1.42906 | 0.958727 | 0.385742 | 3.202955 |
| 48 hours | V-SF1-Q6 | -0.71132 | -0.27617 | 1.624392 | 1.515028 | 1.197356 | 3.517534 |
| 48 hours | V-STAT1-02 | -1.5471 | -1.4372 | 1.673877 | 0.741046 | 0.243015 | 3.15337 |
| 48 hours | V-USF-01 | 0.087928 | -0.30845 | 0.411339 | -0.38203 | -0.09457 | 3.417819 |
| 48 hours | V-USF-Q6 | 0.079534 | -0.84401 | 1.006561 | 1.262549 | 0.562046 | 3.399586 |
| 48 hours | V-USF-Q6-01 | 0.08073 | 1.150563 | 1.182707 | 0.329368 | 0.0775 | 3.059854 |
| 48 hours | V-YY1-02 | 0.8821 | -1.13219 | 1.011632 | -0.07793 | 0.076737 | 3.26191 |
| 48 hours | V-YY1-Q6 | 0.864278 | -1.10931 | 0.36639 | -0.07635 | -0.56252 | 2.968494 |
