# Supplementary material for: Time-Dependent c-Myc Transactomes Mapped by Array-Based Nuclear Run-On Reveal Transcriptional Modules in Human B Cells
Source: PLoS One. 2010 Mar 15;5(3):e9691. doi: 10.1371/journal.pone.0009691 (PMC2837740; doi:10.1371/journal.pone.0009691)
Supplement: Table S5 — (0.04 MB DOC) [file pone.0009691.s006.doc]

**Table S5. Transcriptionally regulated direct Myc target genes determined by ChIP-PET and ANRO (48 hour)**

Up-regulated Down-regulated

ACAT1 ABCA7

ACTR3B ABCA7

AHCY ACADVL

AHR ACBD3

AK2 ADAM8

AK3L2 AHDC1

ALDOA AHNAK

ANAPC1 AKNA

ANKRD41 ALOX5

ANP32B ALOX5AP

ANP32C AMPD3

APEX1 AP1G2

ASS APOB48R

ATAD3B APOBEC3F

ATP5B APOBEC3F

ATP5D APOL3

ATP5J ARHGAP30

ATP5J ARHGEF1

BCS1L ARHGEF2

BMF ARPC4

BOP1 ARRDC1

BXDC2 ARSA

C12orf45 ATG16L2

C14orf156 B2M

C15orf23 BASP1

C17orf45 BAX

C1QBP BCL2L1

C21orf55 BHLHB2

C22orf18 BIK

C3orf32 BLK

C9orf140 BMP2K

CACYBP BMP2K

CAMKV BTG1

CAMSAP1 BTN2A2

CBX6 BTN3A2

CCT7 C12orf35

CDC20 C16orf44

CGI-96 C17orf38

CKAP5 C1orf38

CKS1B C20orf94

CKS2 C22orf13

CYorf15A C6orf32

D15Wsu75e C9orf77

DDX21 CAPN12

DDX39 CBFA2T3

DHCR7 CCDC50

DKFZp434I1020 CCL17

DSCR2 CCM2

DSCR2 CCNDBP1

DTYMK CD180

EBNA1BP2 CD24

EEF1B2 CD300C

EEF2 CD37

EIF4A1 CD40

EIF4B CD6

ENO1 CD72

EPRS CD74

EXOSC2 CD74

EXOSC5 CD79A

FABP5 CD79B

FBL CD79B

FJX1 CD82

FKBP11 CD82

GAL CD84

GAPDH CDC42EP3

GARS CEACAM1

GEMIN5 CENTB1

GLDC CHIA

GLO1 CHKB

GPR30 CNN2

H2AFZ COL9A2

HCAP-G CORO1A

hfl-B5 CREB5

HIST1H1E CSRP1

HIST1H2AB CST3

HIST1H2BE CTDSP1

HIST1H2BH CTNND1

HIST1H3D CTSZ

HIST1H3F CUGBP2

HIST1H3H CYBA

HIST1H4B DBNL

HIST1H4C DENND1C

HIST1H4H DGKZ

HIST1H4I DIP2A

HIST1H4K DKFZP434B0335

HIST2H2AC DNM2

HMG1L1 DOCK10

HNRPA0 DOCK11

HNRPA2B1 DOCK2

HNRPA3 DOK3

HNRPM DPEP2

HPRT1 DTX2

HSP90AB1 DTX3L

HSPD1 DUS2L

HSPE1 EIF2AK2

IARS EMB

IARS2 EMP3

IFRD2 ENTPD1

IL18 EPSTI1

IL7R EVI2B

ILF3 FAIM3

IMPDH2 FBXW4

JAG2 FCER2

K-ALPHA-1 FCGR2A

KIAA0020 FCRL2

KIAA1804 FCRL3

LDHA FCRL5

LDHB FGD3

LEF1 FGFR1

LETM1 FKSG30

LOC197336 FLJ11000

LYAR FLJ20035

MAGOH FLJ20245

MAT2A FLJ22386

MATK FLJ22709

MATR3 FLJ33641

MCM4 FLJ35530

MCM8 FLJ40869

MDH2 FLNA

MGC14289 FMNL3

MRPL21 FRRS1

MRPL23 FSCN1

MRPL24 FSCN2

MRPS15 G1P3

MRPS2 GABARAPL2

MRPS21 GALNT10

NCL GARNL4

NDUFS5 GAS7

NME1 GGT1

NME2 GNAI2

NOC2L GORASP1

NOL5A GPSM3

NOLC1 GPX1

NPDC1 GRIPAP1

NPM1 GRK6

ODC1 GSDMDC1

PA2G4 GSDML

PAICS GSN

PCCB GTF2IRD2

PDCD11 H2AFY

PGAM4 HCK

PGK1 HCST

PHB2 HDAC7A

PHGDH HIP1R

POLD2 HLA-A

POLR1C HLA-B

PPA1 HLA-C

PPARGC1B HLA-DMA

PPIB HLA-DOA

PPP2R3B HLA-DOB

PPP2R3B HLA-DPA1

PRDX1 HLA-DPB1

PRDX3 HLA-DQB1

PRIM1 HLA-DRA

PRR5 HLA-DRB1

PTPLAD2 HLA-DRB3

PUS1 HLA-DRB4

QP-C HLA-E

RANBP1 HSH2D

RCC1 HSPA6

RNU3IP2 HYPE

RPL13A ICOSLG

RPL14 IDUA

RPL15 IER5

RPL18 IFI30

RPL27 IFI35

RPL31 IFI44

RPL38 IFI44L

RPL4 IL10RA

RPL6 IL10RB

RPL7L1 IL4I1

RPLP0 IL4R

RPS10 ILK

RPS14 INPP5D

RPS2 IRF7

RPS25 ISGF3G

RPS5 ITGA4

RPUSD4 ITGAM

RUVBL2 ITGB2

SCD ITM2B

SET ITM2C

SFRS1 JUP

SFRS2 JUP

SLC16A11 KCNK5

SLC25A5 KIAA1370

SNRPF KLF2

SOD1 LAIR2

SRM LBH

SSBP1 LFNG

STRA13 LGALS1

TBCA LGALS3BP

TCEA3 LGALS9

TFDP1 LIMK1

TIGA1 LIMK1

TINP1 LKAP

TKT LMBRD1

TNFRSF8 LOC196913

TOMM40 LOC440836

TOP1MT LOC93349

TPI1 LTB

TRAP1 LY6E

TSC22D1 LYL1

TSR1 LYN

TUFM LYZ

TXN MAN2B1

TXNDC5 MGAT3

UBE2C MGC14376

UCK2 MGC15875

UHRF1 MGC19764

USP13 MGC3123

VDAC2 MGC33926

WDR12 MGC39497

WDR4 MGC50559

WDR54 MMP11

WHSC1 MNDA

XRCC6 MOV10

XTP3TPA MR1

XYLB MS4A1

YBX1 MT2A

MTMR1

MVP

MX1

MYL6

MYO1F

MYO1G

NALP1

NAPSA

NBR2

NCF1

NCF2

NDE1

NECAP2

NEK6

NFKBIA

NFKBIZ

NICN1

NOD3

NPAL2

NTE

OAS1

OAS2

P2RX5

P2RY5

PALM

PARP10

PARP12

PARP15

PARVG

PCDHGB5

PDE1B

PDE4B

PHF11

PIK3CD

PIK3R5

PLEK

POLD4

PPIL5

PPP1R9B

PPP3CC

PQLC1

PREX1

PRF1

PRIC285

PRKCB1

PRKCBP1

PRKD2

PSEN1

PSG11

PTGS1

PTPN6

PTPRC

PTPRCAP

PTTG1IP

PYCARD

RAB11B

RAB24

RAC2

RALGPS2

RARRES3

RASA1

RASSF5

RBM5

REPS2

RGS14

RGS19

RNASET2

S100PBP

SAT

SCAMP2

SCARA5

SCRN1

SEMA4D

SEMA7A

SESN3

SETX

SH2D3C

SH2D3C

SH3BGRL3

SIDT2

SLAMF6

SNAP23

SNX22

SOCS1

SOS1

SP100

SPIB

SPPL2A

SPRR1B

SRPK2

ST3GAL1

ST3GAL2

ST8SIA4

STAT1

STK17B

STX5A

SYK

TAF10

TAGAP

TAGLN

TAP2

TAPBP

TAZ

TCF4

TCIRG1

TCIRG1

TGFBR2

TIMP1

TLE3

TLR4

TLR9

TMBIM4

TMC6

TMC8

TMEM2

TMEM37

TMEM63A

TMEM8

TMEPAI

TNFRSF13B

TNFRSF13C

TNFRSF14

TNIP2

TOR1AIP1

TRPV2

TSPAN32

TTLL3

UBE1L

UBE2H

UCP2

UNC119

UPB1

URP2

USF1

VCL

VPREB3

WAS

WBP2

WDFY2

WIG1

YPEL3

ZBP1

ZNF613

ZNF655
